# Short- and long-term outcomes following COVID-19 or Influenza hospitalization in adults: results of the AUTCOV study

**DOI:** 10.3389/fpubh.2025.1716163

**Published:** 2026-01-12

**Authors:** Christine Wagenlechner, Ralph Wendt, Berthold Reichardt, Michael Mildner, Julia Mascherbauer, Clemens Aigner, Johann Auer, Hendrik Jan Ankersmit, Alexandra Christine Graf

**Affiliations:** 1 Center for Medical Data Science, Medical University of Vienna, Vienna, Austria; 2 Clinic of Thoracic Surgery, Medical University of Vienna, Vienna, Austria; 3 Department of Nephrology, Hospital St. Georg Leipzig, Leipzig, Germany; 4 Austrian Social Health Insurance Fund, Eisenstadt, Austria; 5 Clinic of Dermatology, Medical University of Vienna, Vienna, Austria; 6 Department of Internal Medicine 3, University Hospital St. Poelten, St. Poelten, Austria; 7 Department of Internal Medicine 1 with Cardiology and Intensive Care, St. Josef Hospital Braunau, Braunau am Inn, Austria; 8 Laboratory for Cardiac and Thoracic Diagnosis, Regeneration and Applied Immunology, Vienna, Austria

**Keywords:** COVID-19 hospitalization, Influenza hospitalization, long-term outcomes, medication profile, registry-based observational study

## Abstract

**Introduction:**

Large-scale registry-based studies on patients hospitalized with COVID-19 as compared to Influenza are scant, yet they are needed to re-evaluate the pandemic and the characteristics of patients at risk of severe outcomes.

**Methods:**

In this registry-based study from Austria, we examined short- and long-term outcomes after hospital admission due to COVID-19 or Influenza, also focusing on outcomes conditional on hospital survival. Data were provided on adults hospitalized with COVID-19 in the years 2020 and 2021 or with Influenza in 2016–2021, as well as on matched controls from the Austrian population. Analyses were performed separately for the four age groups (19–40, 41–64, 65–74, and ≥75 years).

**Results:**

Hospitalized COVID-19 and Influenza patients had a larger medication load as compared to the general population. Across all investigated age groups, polypharmacy was more frequent in the Influenza group. The risk for all-cause death in the entire follow-up period and death during hospital stay was higher in the COVID-19 group as compared to the Influenza group for all age groups ≥41 years. Furthermore, the duration of hospitalization was longer in patients with COVID-19. Notably, readmission rates were higher in Influenza patients, and mortality of hospital survivors was increased in younger Influenza patients aged 41-64 compared to COVID.

**Conclusion:**

In the first 2 years of the pandemic, COVID-19 had devastating effects on a non-immunized population, mainly in older patients and in patients with pre-existing serious comorbidities, but the health consequences of Influenza should not be underestimated.

## 1 Introduction

The outbreak of the coronavirus disease 2019, attributed to the zoonotic virus Severe Acute Respiratory Syndrome Coronavirus-Type 2 (SARS-CoV-2), marked the start of an unprecedented pandemic, leading to enormous consequences in public life. Age, gender, and pre-existing comorbidities are well-established predictors of severe COVID-19 outcomes (1–6). Patients hospitalized for COVID-19 were described as more often having a higher comorbidity burden, more severe initial illness, increased rates of polypharmacy, and the risk of short- and long-term outcomes may be additionally associated with underlying comorbidities and health conditions (7–16).

The preventive measures for SARS-CoV-2 infection in the first pandemic years were initially based on a comparison with Influenza viruses because the viruses have similar modes of transmission and cause respiratory diseases. Some studies have directly compared COVID-19 and Influenza hospitalized patients; however, existing literature generally focuses on comparing patient characteristics between cohorts and in-hospital or short-term outcomes up to 30 days (17–24). Large-scale registry-based studies comparing long-term outcomes are scant (25–28).

This nationwide study aims to evaluate and compare short-term outcomes (in-hospital mortality and time to hospital discharge) and long-term outcomes (all-cause mortality and readmission) in hospitalized COVID-19 and Influenza patients and controls from the Austrian population.

## 2 Methods

### 2.1 Study design and cohorts

This retrospective, nationwide, registry-based Austrian COVID-19 (AUTCOV) study was conducted in accordance with the Declaration of Helsinki and approved by the Ethics Committee of Lower Austria (GS1-EK-4/747-2021). Data were obtained from the Austrian Health Insurance Funds, which cover approximately 98% of the Austrian population, through the public health insurance system. Austria's national healthcare system provides broad access to medical services, is characterized by a low incidence of malpractice litigation, and shows limited tendencies toward the overuse of medical resources. Access to specific health services is governed by social insurance legislation.

The study included all adult patients older than 18 years of age hospitalized in Austria due to the main diagnosis of COVID-19 (ICD-10 Codes U071, U072, U049) from 1 January 2020 to 31 December 2021 (i.e., including patients from the first four pandemic waves) and patients older than 18 years hospitalized in Austria due to the main diagnosis of Influenza (ICD-10 codes: J09, J100, J101, J108, J110, J111, J118, J10) from 1 January 2016 to 31 December 2021. Patient characteristics (age, gender, region, and medication profiles) were obtained from 1 year before index hospitalization until the study cut-off. For both patient groups, random age-, gender-, and region-matched control groups (approximately 10 controls for each patient) consisting of individuals who were not hospitalized due to COVID-19 (in the years 2020 and 2021) or Influenza (in the years 2016–2021) were chosen from the population registered in the Austrian Health Insurance Fund. Data on the control groups were available from 1 year before the first patient was hospitalized until the study cut-off. Death dates were available until the study cut-off. Note that this study followed similar approaches to previously published AUTCOV studies (16, 29).

### 2.2 Study outcomes

The primary outcome was the time from hospital admission to death due to any reason (all-cause death). Secondary long-term outcomes were time from index hospital discharge to all-cause death, as well as time to readmission for any reason in survivors of the index hospitalization. Secondary short-term outcomes were time from hospital admission to hospital discharge, as well as to death during the index hospital stay (in-hospital death). For a detailed description of the outcomes, see Supplementary Table S1.

### 2.3 Statistical analysis

Since age is an important risk factor for severe outcomes, the cohort was split into four independent cohorts based on age. All analyses were performed separately for the age cohorts 19–40, 41–64, 65–74, and ≥75 years (Supplementary Table S2). For each COVID-19 or Influenza patient, characteristics such as age, gender, and Anatomical Therapeutic Chemical (ATC) codes describing the patients' medication profile were available from the Austrian Health Insurance Funds from 1 year before the index hospitalization. For statistical analyses, ATC codes before hospitalization were summarized in 30 medication groups (Supplementary Table S3) using a binary variable defined as 1 for the medication group if a drug of the corresponding ATC codes was prescribed at least once a year before the index hospitalization, and 0 if not. Data were evaluated for the following 30 medication groups: All medication groups were summed up for the number of medication groups, categorized into 0–1, 2–5, 6–10, and ≥11. In this study, polypharmacy refers to ≥6 medication groups. The medication groups rhinological and throat antiseptics (MG25), hormonal contraceptives and similar hormone preparations (MG18), and cold and cough preparations (MG29) were excluded because they were not assumed to be associated with severe underlying diseases. For controls, a similar medication profile was generated using the drugs prescribed 1 year before the index hospital stay of the matched patient. Numbers and percentages were used to summarize categorical variables, and medians and interquartile ranges were used for continuous variables. Medication groups were compared between patients and controls, as well as between patients with Influenza and COVID-19, using chi-squared tests or Fisher's exact tests, as appropriate.

To compare outcomes of COVID-19 and Influenza hospitalized patients, propensity score matching (PSM) was performed to account for bias due to potentially unbalanced cohorts. The propensity score matching was performed separately for the age groups and was estimated with logistic regression based on age, gender, and all medication groups, with a *p*-value of < 0.05 in the comparison between cohorts using the chi-squared test and Fisher's exact test. To evaluate the association between the patient groups (COVID/Influenza) and time to all-cause death, for each medication group, first, simple Cox regression models were calculated accounting for patient group, gender, age, polypharmacy, wave (i.e., half year of COVID-19/Influenza infection), and the respective medication group with the clustering variable region. For the secondary outcomes of in-hospital all-cause death and post-discharge all-cause death, the regression models followed the same approach, with the addition of the confounder length of hospital stay for post-discharge all-cause death. Further, a multivariable Cox regression model was performed, including gender, age, polypharmacy, wave, and all medication groups with a *p*-value smaller than 0.1 in the simple models. To evaluate the association between group (COVID-19, Influenza) and time to hospital discharge, first, for each medication group, simple Fine and Gray regression models (accounting for the competing risk of death) were calculated, accounting for group, gender, age, polypharmacy, wave, and the respective medication group with clustering variable region. A multivariable model was performed, including gender, age, polypharmacy, wave, and all medication groups with a *p*-value smaller than 0.1 in the simple models. Time to readmission was analyzed in a manner similar to that of hospital discharge. Schönfeld residuals were used to evaluate the proportional hazard assumption, and variance inflation factors were used to evaluate multicollinearity.

For all-cause death, death conditional on hospital survival, readmission, and in-hospital death, a hazard ratio smaller than one indicates a larger probability of the event in the COVID-19 group. For time to discharge, a hazard ratio larger than one indicates a smaller probability of hospital discharge in the COVID-19 group, i.e., patients in the COVID-19 group are more likely to have a longer hospital stay. Due to the retrospective and exploratory nature of the study, a *p*-value of < 0.05 was considered statistically significant, and no correction for multiplicity was applied. All analyses were performed using R version 4.2.2 (30).

## 3 Results

**Figure 1:**
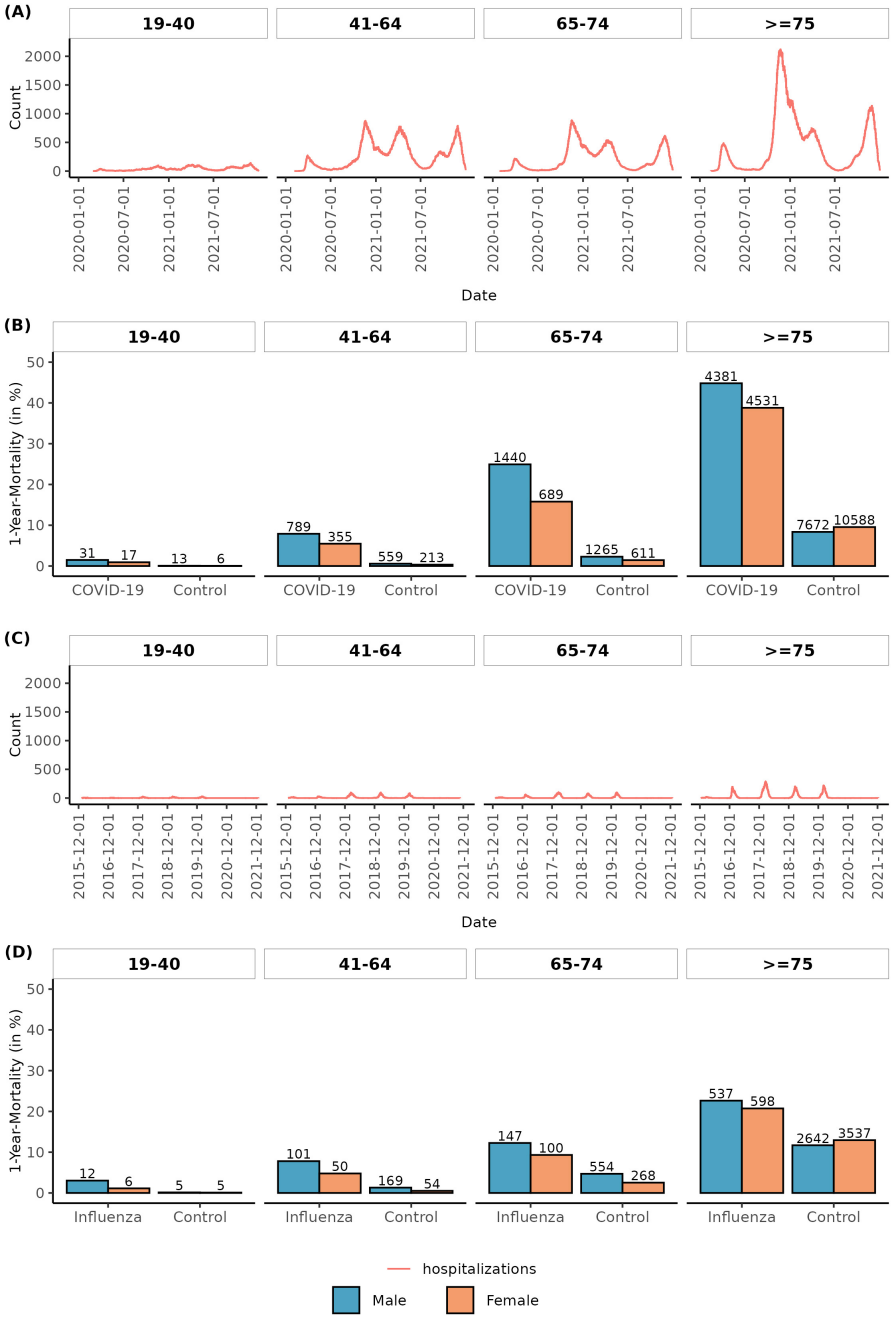
Timeline and 1-year mortality by age group. Number of COVID-19 **(A)** and Influenza **(C)** patients in the hospital over time in the hospitalization observation period in age groups. One-year mortality in age groups and by gender for COVID-19 **(B)** and Influenza **(D)** patients and corresponding age-, gender-, and region-matched control groups.

The study included 52,015 patients hospitalized with COVID-19 and 10,796 patients hospitalized with Influenza, who were divided into four age groups (19–40, 41–64, 65–74, and ≥75 years; Table 1, Figures 1A, C). Furthermore, data on 501,516 age-, gender-, and region-matched COVID-19 and 104,872 Influenza controls with a follow-up in the timeline, as the matched patients, were randomly chosen from the Austrian population. The median follow-up time for all age groups was >400 days in the COVID group and >1200 days in the Influenza group (Table 1). Among the COVID-19 hospitalized patients, 7.6% were aged 19–41, 31.7% were aged 41–64, 19.5% were aged 65–74, and 41.2% were aged > 74 years. The numbers for Influenza patients were 8.6% (19–41), 21.7% (41–64), 21.0% (65–74), and 48.7% (≥75).

**Table 1:** Demographic data for patients hospitalized with COVID-19 and Influenza in Austria.

**Patients with COVID-19**									
**Age**		**19–40**		**41–64**		**65–74**		≥**75**	
		***n*** = **3,941**		***n*** = **16,481**		***n*** = **10,140**		***n*** = **21,453**	
		***n***	**%**	***n***	**%**	***n***	**%**	***n***	**%**
Gender	M	2,092	53.08	9,992	60.63	5,776	56.96	9,774	45.56
	W	1,849	46.92	6,489	39.37	4,364	43.04	11,679	54.44
Number of prescribed medication groups	0–1	2,382	60.44	5,996	36.38	1,623	16.01	2,475	11.54
	2–5	1,386	35.17	7,859	47.69	4,955	48.87	10,297	48.00
	6–10	160	4.06	2,374	14.40	3,052	30.10	7,852	36.60
	≥11	13	0.33	252	1.53	510	5.03	829	3.86
Follow–up in days	Median (IQR)	448 (280–581)		470 (298–587)		513 (377.5–593)		539 (316–592)	
Time to death	Median (IQR)	19.5 (8.25–50.25)		23 (11–55)		19.5 (9–52)		15 (7–52)	
Length of hospital stay	Median (IQR)	6 (3–9)		9 (5–14)		11 (7–19)		12 (7–20)	
**Influenza patients**									
**Age**		**19–40**		**41–64**		**65–74**		≥**75**	
		***n*** = **925**		***n*** = **2,342**		***n*** = **2,272**		***n*** = **5,257**	
		***n***	**%**	***n***	**%**	***n***	**%**	***n***	**%**
Gender	M	396	42.81	1,295	55.29	1,199	52.77	2,372	45.12
	W	529	57.19	1,047	44.71	1,073	47.23	2,885	54.88
Number of prescribed medication groups	0–1	503	54.38	591	25.23	301	13.25	565	10.75
	2–5	359	38.81	1,063	45.39	983	43.27	2,604	49.53
	6–10	60	6.49	587	25.06	830	36.53	1,859	35.36
	≥11	3	0.32	101	4.31	158	6.95	229	4.36
Follow-up in days	Median (IQR)	1,240 (896–1,608)		1,257 (925.25–1,608)		1,259 (918–1,614)		1,263 (901–1,612)	
Time to death	Median (IQR)	302.5 (105.5–674.5)		410 (95–848)		539 (167.5–990)		470 (92–967)	
Length of hospital stay	Median (IQR)	4 (3–6)		6 (4–8)		7 (5–9)		8 (6–11)	

### 3.1 Medication profile of COVID-19 and Influenza hospitalized patients

Overall, age groups had a larger medication load for patients hospitalized due to COVID-19, as compared to the general population. For most medication groups, a significant difference from the general population was observed (Supplementary Tables S4–S7). Whereas, for the youngest age group (19–41 years), 4.4 % patients had prescriptions in at least six medication groups (polypharmacy), all other age groups showed much larger percentages, increasing to more than 30% in the older age groups (Table 1). Similar results were found for the Influenza group as compared to the general population (Supplementary Tables S8–S11). Polypharmacy was present in 61.2% of patients hospitalized with Influenza aged 19–41 years, increasing to 89.3% in the oldest age group. Generally, over the investigated age groups, a larger percentage of polypharmacy was observed for Influenza (Table 1). A more detailed comparison of medication groups between COVID-19 and Influenza patients is available in the supplement (Supplementary Tables S12–S15).

### 3.2 Comparison between PSM-matched COVID-19 and Influenza outcomes

**Figure 2:**
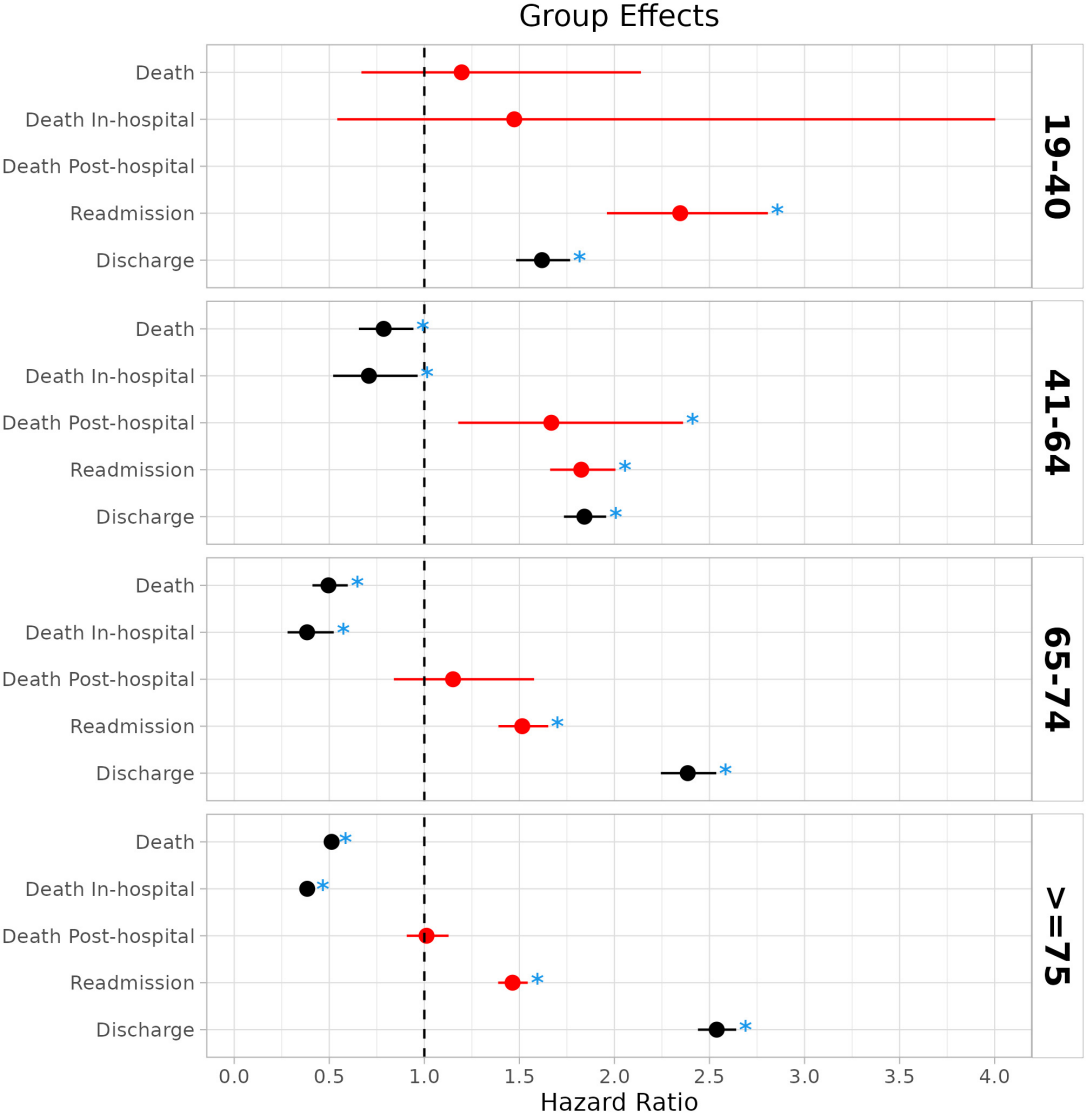
Hazard ratios and 95% confidence intervals for group effects comparing COVID-19 and Influenza patients for primary and secondary outcomes in the age groups. A hazard ratio larger than one indicates a higher risk for the event in the Influenza group. For hospital discharge, a hazard ratio larger than one indicates that patients with COVID-19 are more likely to have a longer hospital stay. For illustration, adverse outcomes for Influenza patients in reference to patients with COVID-19 are marked in red. Significant comparisons (*p* < 0.05) are marked with blue stars.

*All-cause mortality*: Whereas no statistically significant difference in all-cause death between patients hospitalized with COVID-19 and Influenza was observed for the age group 19–40, a significantly lower risk for all-cause death for Influenza patients was found in the group of patients aged 41–64 (HR: 0.79, 95% CI:0.66–0.94, *p* = 0.009, aged 65–74 (HR: 0.50, 95% CI:0.41–0.60, *p* < 0.001), and patients older than 74 (HR: 0.51, 95% CI: 0.49–0.53, *p* < 0.001, Figure 2, Supplementary Tables S16–S19, Supplementary Figures S1, S7). One-year mortality was 1.25% (19–40), 7.06% (41–64), 21.34% (65–74), and 42.16% (≥75) in the COVID-19 cohort as compared to 1.95% (19–40), 6.45% (41–64), 10.87% (65–74), and 21.59% (≥75) in the Influenza cohort (Figures 1B, D).

Within the COVID-19 cohort over all age groups, men showed a significantly larger risk for all-cause death as compared to women (all *p* < 0.001, Supplementary Tables S20–S23). In simple regression models, a trend toward a higher risk of all-cause death was observed for patients with a larger number of prescribed medications. However, this trend only remained significant in the multivariable analyses (additionally accounting for individual medication groups) in the age group 41–64 (Supplementary Tables S20–S23, Supplementary Figures S2, S8), indicating that polypharmacy may not be a reliable indicator for the underlying health status of a patient and the association with all-cause death. Within the Influenza cohort, except for the youngest age group, a significantly worse survival probability was observed in men compared to women (Supplementary Tables S24–S27). As in the COVID-19 cohort, a trend toward a higher risk of all-cause death was observed for patients with a larger number of prescribed medications. This trend remained significant in the multivariable models for patients aged >40 years (Supplementary Tables S24–S27, Supplementary Figures S2, S8).

*In-hospital mortality*: No statistically significant difference in in-hospital mortality between COVID-19 and Influenza patients was observed for the youngest age group (Figure 2 and Supplementary Figures S3, S7, S9, Supplementary Table S28); however, a lower risk for in-hospital death in Influenza patients was observed in the groups of patients aged 41–64 (HR: 0.71, 95% CI: 0.52–0.96, *p* = 0.029, Figure 2 and Supplementary Figures S3, S7, Supplementary Table S29), 65–74 (HR: 0.38, 95% CI:0.28–0.52, *p* < 0.001, Figure 2 and Supplementary Figures S3, S7, Supplementary Table S30), and patients older than 74 years (HR: 0.38, 95% CI: 0.35–0.42, *p* < 0.001, Figure 2 and Supplementary Figures S3, S7, Supplementary Table S31). In-hospital mortality was 0.86% (19–40), 5% (41–64), 15.32% (65–74), and 28.97% (≥75) in the COVID-19 cohort and 0.54% (19–40), 2.31% (41–64), 3.12% (65–74), and 6.62% (≥75) in the Influenza cohort.

*All-cause mortality in hospital survivors*: All-cause mortality in hospital survivors was not significantly different in the age groups 65–74 and ≥75 years (Supplementary Tables S32–S35, Figure 2, and Supplementary Figures S4, S7). The regression models could not be calculated for the age group 19–40 due to the small number of out-of-hospital deaths. For the age group 41–64, a larger risk of death after hospital discharge was observed in the Influenza group (HR: 1.67, 95% CI: 1.18–2.36, *p* = 0.004, Supplementary Table S34, Figure 2, and Supplementary Figures S4, S7). However, the majority of deaths in the COVID-19 group occurred in-hospital and not after hospital discharge.

*Readmission*: A significantly larger probability of readmission for Influenza patients was observed over all age groups (19–40: HR: 2.35, 95% CI: 1.96–2.81, *p* < 0.001; 41–64: HR: 1.83, 95% CI: 1.66–2.01, *p* < 0.001, 65–74: HR: 1.51, 95% CI: 1.39–1.65, *p* < 0.001, ≥75: HR: 1.46, 95% CI: 1.39–1.54, *p* < 0.001, Figure 2 and Supplementary Figures S5, S10, Supplementary Tables S36–S39), observing that this effect decreases with increasing age. However, in-hospital mortality was higher in the COVID-19 population in the older age groups.

*Hospital discharge*: Over all age groups, a significantly longer time to hospital discharge was found for patients with COVID-19 (19–40: HR: 1.62, 95% CI: 1.48–1.77, *p* < 0.001; 41–64: HR: 1.84, 95% CI: 1.73–1.96, *p* < 0.001, 65–74: HR: 2.39, 95% CI: 2.24–2.54, *p* < 0.001, ≥75: HR: 2.54, 95% CI: 2.44–2.64, *p* < 0.001, Figure 2 and Supplementary Figures S6, S10, and Supplementary Tables S40–S43).

## 4 Discussion

Our study represents one of the largest data collections to date comparing short- and long-term outcomes of COVID-19 and Influenza patients. In this retrospective, nationwide study based on registry data available from the Austrian Insurance Funds, the risk for in-hospital death and all-cause death (in a long-term follow-up) was observed to be higher in patients with COVID-19 compared to patients with Influenza aged ≥41 years. One-year mortality was substantially higher in the COVID cohort compared to the Influenza cohort. Furthermore, a longer time to hospital discharge was found for COVID-19 patients across all age groups.

The short-term results on in-hospital mortality and time to hospital discharge are in line with other studies from Austria, Switzerland, France, Canada, the US, and the UK, which also found substantially higher in-hospital mortality for COVID-19 as compared to Influenza (18–23). Also, looking at critically ill patients only in comparing ICU patients with COVID-19 and Influenza, patients with COVID-19 had higher in-hospital mortality and longer ICU stays (24). A significantly higher mortality rate was particularly noticeable in a study of immunocompromised patients. Kidney transplant recipients with COVID-19 had a significantly higher in-hospital mortality rate in 2020 than recipients with Influenza (14.09% vs. 2.61%) (39). When comparing short-term outcomes between patients hospitalized with COVID-19 with those of Influenza patients from previous Influenza seasons at the same hospital at a Tertiary Care Center in the US, COVID-19 resulted in more weekly hospitalizations, higher morbidity, longer length of hospital stay, and higher mortality than Influenza (20% vs. 3%) (23). Furthermore, our study observed, in accordance with the literature (16, 18, 19), over all age groups, a larger medication load for patients hospitalized due to COVID-19 or Influenza as compared to the general population. Polypharmacy was observed more often in the Influenza group.

However, results of nationwide studies for long-term outcomes and the post-acute sequelae of patients hospitalized with COVID-19 as compared to Influenza are scant. Increased mortality of survivors of COVID-19 hospitalization in younger patients < 65 years compared to COVID-19 negative controls has been reported (15). Xie et al. (28) published US data from 81,280 patients admitted to hospital for COVID-19 between March 2020 and June 2022 and 10,985 participants admitted to hospital for seasonal Influenza between 2015 and 2019. Patients were followed up for up to 18 months. Hospital admission for COVID-19 was associated with higher long-term risks of death and adverse health outcomes in nearly every organ system than hospital admission for seasonal Influenza. Oseran et al. (26) investigated 883,394 patients aged ≥65 years discharged alive after an index hospital admission with COVID-19 between 2020 and 2022, compared with 56,409 historical controls discharged alive after a hospital admission with Influenza between 2018 and 2019. After weighting, the COVID-19 cohort had a higher risk of all-cause death after discharge from the hospital at 30 days (10.9% vs. 3.9%), 90 days (15.5% vs. 7.1%), and 180 days (19.1% vs. 10.5%) compared with the Influenza cohort. The COVID-19 cohort also experienced a higher risk of hospital readmission at 30 days (16.0% vs. 11.2%) and 90 days (24.1% vs. 21.3%), but a similar risk at 180 days. The results on readmission are in contradiction to the findings of our study, where hospital survivors had a higher probability of readmission in the Influenza group as compared to the COVID-19 group. In our study, we even observed a higher mortality in the Influenza cohort of hospital survivors compared to COVID-19 in the age group 41–64 years. There was no significant difference in mortality among hospital survivors in the older age groups. A similar observation was also reported in (23). In this study, a higher COVID-19 mortality was observed, but in survivors of hospitalization, readmission within 30 days was higher in Influenza patients. However, the literature on outcomes after hospital survival describes inconsistent results. In the UK, a study of patients discharged from a COVID-19 hospital admission showed higher risks of all-cause mortality, readmission, or death due to the initial infection than Influenza patients (25). Another study from the UK comparing patients hospitalized with COVID-19 during the UK's first pandemic wave in 2020 and Influenza during 2018 and 2019 (27) observed that patients in the COVID-19 cohort were more likely to die in the hospital and within 90 days of discharge. However, for those who survived, rates of emergency readmission were comparable between the COVID-19 and Influenza cohorts.

The main strength of this study is the use of a large, representative, real-world national database from the Austrian Insurance Fund, which provides detailed information on demographics and medication with long-term follow-up. Age-, gender-, and region-matched controls from the general Austrian population were made available at a 1:10 ratio. Another strength is the long observation period; however, long-term results on COVID-19 were only available from the first two pandemic years. Together with our presented data, there is increasing evidence that the short- and long-term mortality of older hospitalized patients with SARS-CoV-2 infection is higher than that of Influenza patients. However, this applies primarily to comparisons between the early stages of the pandemic and historical Influenza seasons. Timely comparisons were not possible since there were very few Influenza infections during the first years of the pandemic, probably due to all the preemptive and exposure-avoidance measures. It should be noted, however, that despite all statistical adjustments, these comparisons should be taken with caution when conclusions are drawn that may underestimate Influenza. In 2020 and 2021, in particular, the new pandemic virus encountered a population that was practically immunologically naive. This is not true for Influenza to the same extent, as a certain basic immunity was mostly present and, in particular, the most vulnerable were able to make use of vaccination options. Furthermore, there is an available treatment for Influenza that reduces mortality (31–33). In the first waves of the COVID-19 pandemic, many hospitals were at least partially overwhelmed, and excess mortality due to structural and logistic shortcomings cannot be ruled out and is even likely. All these factors potentially put the presented COVID-19 group at a disadvantage compared to Influenza. These assumptions are supported by data showing smaller or no mortality differences when comparing COVID-19 and Influenza outcomes in the early vs. later years of the pandemic and no mortality differences when comparing vaccinated patients only (34). On the contrary, there are studies from Australia, Sweden, and Switzerland showing higher mortality even in later stages of the SARS-CoV-2 pandemic with a predominance of omicron variants compared to Influenza (35–37), which strengthens the association of the worse outcomes and higher mortality with COVID-19 compared to Influenza.

While our data offer broad population coverage and real-world insight, they also have important limitations. Our study relies on retrospective observational data derived from insurance registries, which are also dependent on the accuracy of hospital visit documentation. A further limitation is the lack of detailed clinical information in the registry-based data. Variables such as comorbidities, vaccination status, and ICU admission details may have been incompletely recorded. To mitigate potential confounding between the examined patient groups, PSM was employed to balance the patient groups. Furthermore, analyses were performed separately for age groups and different outcomes to provide a detailed overview of the data. Nevertheless, unknown confounding factors remain a concern. Furthermore, medication history information was used as a proxy for patients' comorbidity burden because prescriptions may be documented more precisely and may reflect underlying health conditions more reliably. However, if medication costs are low, patients may pay from their private budgets, resulting in undocumented medication use. While this approach might improve the validity of comorbidity adjustment to a certain degree, it still falls short of the granularity provided in clinical trials or prospective cohort studies where comorbidities are actively recorded and validated. Regarding vaccination status, COVID-19 vaccination in Austria began in December 2020; therefore, COVID-19 vaccination may not be a relevant factor for patients hospitalized in 2020; however, it may have influenced the comparison to Influenza outcomes in later pandemic years. However, vaccine coverage for both COVID-19 and Influenza is generally assumed to be low in Austria, although it may be higher among patients at increased risk of hospitalization or severe outcomes (38).

## 5 Conclusion

In this nationwide study, we observed that patients hospitalized with COVID-19 and Influenza had a larger medication load as compared to the general population. Overall, polypharmacy was more frequent in the Influenza group. The probability of in-hospital death and long-term all-cause death was substantially higher in the COVID-19 cohort compared to the Influenza cohort for patients older than 40 years. A longer time to hospital discharge was observed in the COVID-19 cohort for all age groups. Notably, readmission rates were higher in Influenza patients, and the mortality rate of hospital survivors was higher in younger Influenza patients aged 41–64 compared to COVID-19 patients. These results evaluated that COVID-19 had devastating effects in a non-immunized population, mainly in older patients and patients with pre-existing serious comorbidities, but the health consequences of Influenza should not be underestimated.

## Data Availability

The data analyzed in this study is subject to the following licenses/restrictions: Due to data protection, the datasets presented in this article are not readily available. Data that support the findings of this study are available upon reasonable request from the corresponding author. Requests to access these datasets should be directed to hendrik.ankersmit@meduniwien.ac.at.
