# Supplementary material for: Short- and long-term outcomes following COVID-19 or Influenza hospitalization in adults: results of the AUTCOV study
Source: Front Public Health. 2026 Jan 12;13:1716163. doi: 10.3389/fpubh.2025.1716163 (PMC12832539; doi:10.3389/fpubh.2025.1716163)

Definitions of primary and secondary outcomes

All definitions are according to previous published work of the AUTCOV-Study:

- Graf A., Reichardt B., Wagenlechner C. et al. (2025) Baseline Medication Load and Long-Term Outcomes in COVID-19 Hospitalized Patients: Results of the 2020 AUTCOV Study, Frontiers in Public Health 13: 1565677
- Wagenlechner C., Wendt R., Reichardt B., et al. (2025) Short- and long-term outcomes of children and adolescents hospitalized with COVID-19 or influenza: results of the AUTCOV study, Scientific Reports, 15(1): 22692.

This study includes all patients aged >18 years who were hospitalized in Austria due to the main diagnosis COVID-19 (ICD-10 Codes U071, U072, U049) from 1st January 2020 to 31th December 2021 and Influenza (ICD-10 Codes J09, J100, J101, J108, J110, J111, J118, J10) from 1st January 2016 to 31th December 2021. The patient data were available from the Austrian Health Insurance Funds. The data set includes 52015 COVID-19 hospitalized patients from the first four pandemic waves. 10796 Influenza hospitalized patients were included. For each patient, medication prescriptions (based on ATC codes) were available from 1 year before the index COVID or Influenza hospital stay to study cut-off.

A control group (age-, sex- and region-matched, approximately 1:10) not hospitalized due to the main diagnosis COVID-19 in the year 2020 and 2021 was randomly chosen from the population registered in the Austrian Health Insurance Funds to represent the general Austrian population. It should be noted, that the control population can (but does not necessarily have to) be hospitalized in the follow-up time due to any reason with the exception of COVID-19. ATC codes for the control groups were available from 1 year before the first COVID patient was hospitalized in 2020 up to the study cut-off. The control group for the COVID-19 patients included 501516 cases. Death dates were available at the time of study cut-off.
Similarly, a second control group was randomly chosen from the population not hospitalized due to the main diagnosis Influenza in the years 2016 to 2021 including 104842.

Primary parameter was all-cause death defined as the time from hospital admission to death due to any reason (based on death-date available at study cut-off).
Secondary outcomes were all-cause death and readmission conditioned on hospital survival as well as in-hospital death and time to hospital discharge (as short-term outcomes). To evaluate the outcome readmission due to any reason for each patient, data were scanned from the index COVID- and Influenza-hospital stay to the study cut-off date. Readmissions due to COVID-19 and Influenza within 2 weeks after the index hospital stay were considered to belong to the index hospital stay. All outcome definitions are summarized in **Table S1**.

Table S1. Definitions of primary and secondary outcomes

Outcome	Definition
**All-cause mortality (primary outcome)**	all-cause mortality based on death date: time from COVID-19/Influenza hospital admission to death or last follow-up
**All-cause mortality after hospital survival (secondary outcome)**	all-cause mortality based on death date for the subgroup of patients surviving hospital stay: time from (alive) COVID-19/Influenza hospital discharge to death or last follow-up
**Readmission due to any reason (secondary outcome)**	based on billing information (MEL-Code): time from (alive) COVID-19/Influenza hospital discharge to the first hospital admission (due to any reason) after index hospital stay
**Hospital discharge (secondary outcome)**	based on date of hospital discharge: time from COVID-19/Influenza hospital admission to hospital discharge or death
**In-hospital mortality**	based on death date: time from index COVID-19/Influenza hospital admission to death before hospital discharge or hospital discharge.

Definitions of confounders

The statistical models were calculated separately for four age groups: 19-40 years, 41-64 years, 65-74 years, ≥75 years. Within age groups, the variable age was categorized into the categories described in **Table S2**.

Table S2: Categorization of age groups

Age groups	Categorization within age groups
**19-40 years**	AG1: 19-25, AG2: 26-30, AG3: 31-35, AG-R: 36-40 (reference)
**41-64 years**	AG1: 41-45, AG2: 46-50, AG3: 51-55, AG4: 56-60, AG-R: 61-64 (reference)
**65-74 years**	AG1: 65-70, AG-R: 71-74 (reference)
**≥75 years**	AG1: 75-80, AG2: 81-85, AG3: 86-90, AG-R: >90 (reference)

For COVID-19 patients, the confounder 'wave' indicates the period during which patients were hospitalized for COVID between January 2020 to December 2020, January 2021 to June 2021 or July 2021 to December 2021.
For influenza patients, the confounder 'wave' was specified for the hospitalization periods January 2016 to June 2016, July 2016 to June 2017, July 2017 to June 2018, July 2018 to June 2019, July 2019 to June 2020 and July 2020 to December 2021.

ATC codes describing prescribed medication were available from the Austrian Health Insurance Funds 1 year before the index-hospitalization due to COVID-19 and influenza. ATC codes for medications before hospitalization were summarized into medication groups (Table S3) using binary variables, which were set to 1 if the patient received at least one medicament from the corresponding medication group at least once in the year before COVID-19 and influenza hospitalization.
All medication groups except “Hormonal contraceptives and similar hormone preparations”, “rhinological and throat antiseptics”, “Cold and cough preparations” were used to define polypharmacy as the sum of medication groups with at least one pre-scribed medication group, categorized into 0-1, 2-5, 6-10 and ≥11 medication groups for individuals aged 41 and older, and categories 0-1, 2-5 ≥6 for age group 19-40 years. The three excluded medication groups from the summation were assumed to not to be associated with severe underlying diseases potentially influencing outcomes.

Table S3: Definitions of medication groups

Medication group	ATC codes
**Anticoagulants**	B01AA01, B01AA02, B01AA03, B01AA04, B01AA07, B01AA08, B01AA09, B01AA10, B01AA11, B01AA12, B01AB01, B01AB02, B01AB04, B01AB05, B01AB06, B01AB07, B01AB08, B01AB09, B01AB10, B01AB11, B01AB12, B01AB51, B01AC01, B01AC02, B01AC03, B01AC04, B01AC05, B01AC06, B01AC07, B01AC08, B01AC09, B01AC10, B01AC11, B01AC13, B01AC14, B01AC15, B01AC16, B01AC17, B01AC18, B01AC19, B01AC21, B01AC22, B01AC23, B01AC24, B01AC25, B01AC27, B01AC30, B01AC56, B01AD01, B01AD02, B01AD03, B01AD04, B01AD05, B01AD06, B01AD07, B01AD08, B01AD09, B01AD10, B01AD11, B01AD12, B01AE01, B01AE02, B01AE03, B01AE04, B01AE05, B01AE06, B01AE07, B01AF01, B01AF02, B01AF03, B01AF04, B01AX01, B01AX04, B01AX05, B01AX06
**Antibiotics, antivirals, antiprotozoals, or anthelmintics**	A07AA01, A07AA02, A07AA03, A07AA04, A07AA05, A07AA06, A07AA07, A07AA08, A07AA09, A07AA10, A07AA11, A07AA12, A07AA13, A07AA51, A07AA54, A07AB02, A07AB03, A07AB04, A07AC01, J01AA01, J01AA02, J01AA03, J01AA04, J01AA05, J01AA06, J01AA07, J01AA08, J01AA09, J01AA10, J01AA11, J01AA12, J01AA13, J01AA14, J01AA15, J01AA20, J01AA56, J01BA01, J01BA02, J01BA52, J01CA01, J01CA02, J01CA03, J01CA04, J01CA05, J01CA06, J01CA07, J01CA08, J01CA09, J01CA10, J01CA11, J01CA12, J01CA13, J01CA14, J01CA15, J01CA16, J01CA17, J01CA18, J01CA19, J01CA20, J01CA51, J01CE01, J01CE02, J01CE03, J01CE04, J01CE05, J01CE06, J01CE07, J01CE08, J01CE09, J01CE10, J01CE30, J01CF01, J01CF02, J01CF03, J01CF04, J01CF05, J01CG01, J01CG02, J01CR01, J01CR02, J01CR03, J01CR04, J01CR05, J01CR50, J01DA01, J01DA02, J01DA03, J01DA04, J01DA05, J01DA06, J01DA07, J01DA08, J01DA09, J01DA10, J01DA11, J01DA12, J01DA13, J01DA14, J01DA15, J01DA16, J01DA17, J01DA18, J01DA19, J01DA21, J01DA22, J01DA23, J01DA24, J01DA25, J01DA26, J01DA27, J01DA30, J01DA31, J01DA32, J01DA33, J01DA34, J01DA35, J01DA36, J01DA37, J01DA38, J01DA39, J01DA40, J01DA41, J01DA42, J01DA63, J01DB01, J01DB02, J01DB03, J01DB04, J01DB05, J01DB06, J01DB07, J01DB08, J01DB09, J01DB10, J01DB11, J01DB12, J01DC01, J01DC02, J01DC03, J01DC04, J01DC05, J01DC06, J01DC07, J01DC08, J01DC09, J01DC10, J01DC11, J01DC12, J01DC13, J01DC14, J01DD01, J01DD02, J01DD03, J01DD04, J01DD05, J01DD06, J01DD07, J01DD08, J01DD09, J01DD10, J01DD11, J01DD12, J01DD13, J01DD14, J01DD15, J01DD16, J01DD18, J01DD52, J01DD54, J01DD62, J01DD63, J01DD64, J01DE01, J01DE02, J01DF01, J01DF02, J01DH02, J01DH03, J01DH04, J01DH06, J01DH51, J01DH52, J01DH55, J01DH56, J01DI01, J01DI02, J01DI04, J01DI54, J01EA01, J01EA02, J01EB01, J01EB02, J01EB03, J01EB04, J01EB05, J01EB06, J01EB07, J01EB08, J01EB20, J01EC01, J01EC02, J01EC03, J01EC20, J01ED01, J01ED02, J01ED03, J01ED04, J01ED05, J01ED06, J01ED07, J01ED08, J01ED09, J01ED20, J01EE01, J01EE02, J01EE03, J01EE04, J01EE05, J01EE06, J01EE07, J01FA01, J01FA02, J01FA03, J01FA05, J01FA06, J01FA07, J01FA08, J01FA09, J01FA10, J01FA11, J01FA12, J01FA13, J01FA14, J01FA15, J01FF01, J01FF02, J01FG01, J01FG02, J01GA01, J01GA02, J01GB01, J01GB03, J01GB04, J01GB05, J01GB06, J01GB07, J01GB08, J01GB09, J01GB10, J01GB11, J01GB12, J01GB13, J01GB14, J01MA01, J01MA02, J01MA03, J01MA04, J01MA05, J01MA06, J01MA07, J01MA08, J01MA09, J01MA10, J01MA11, J01MA12, J01MA13, J01MA14, J01MA15, J01MA16, J01MA17, J01MA18, J01MA19, J01MA21, J01MA22, J01MA23, J01MA24, J01MB01, J01MB02, J01MB03, J01MB04, J01MB05, J01MB06, J01MB07, J01RA01, J01RA02, J01RA03, J01RA04, J01XA01, J01XA02, J01XA03, J01XA04, J01XA05, J01XB01, J01XB02, J01XC01, J01XD01, J01XD02, J01XD03, J01XE01, J01XE02, J01XX01, J01XX02, J01XX03, J01XX04, J01XX05, J01XX06, J01XX07, J01XX08, J01XX09, J01XX11, J01XX12, J02AA01, J02AA02, J02AB01, J02AB02, J02AC01, J02AC02, J02AC03, J02AC04, J02AC05, J02AX01, J02AX04, J02AX05, J02AX06, J04AA01, J04AA02, J04AA03, J04AB01, J04AB02, J04AB03, J04AB04, J04AB05, J04AB06, J04AB30, J04AC01, J04AC51, J04AD01, J04AD02, J04AD03, J04AK01, J04AK02, J04AK03, J04AK04, J04AK05, J04AK06,

	J04AK07, J04AM01, J04AM02, J04AM03, J04AM04, J04AM05, J04AM06, J04AM07, J04AM08, J04BA01, J04BA02, J04BA03, P01AA01, P01AA02, P01AA04, P01AA05, P01AA52, P01AB01, P01AB02, P01AB03, P01AB04, P01AB05, P01AB06, P01AB07, P01AC01, P01AC02, P01AC03, P01AC04, P01AR01, P01AR02, P01AR03, P01AR53, P01AX01, P01AX02, P01AX04, P01AX05, P01AX06, P01AX07, P01AX08, P01AX09, P01AX10, P01AX11, P01AX52, P01BA01, P01BA02, P01BA03, P01BA06, P01BA07, P01BB01, P01BB02, P01BB51, P01BC01, P01BC02, P01BD01, P01BD51, P01BE01, P01BE02, P01BE03, P01BE04, P01BE05, P01BE52, P01BF01, P01BF02, P01BF03, P01BF04, P01BF05, P01BF06, P01BX01, P01CA02, P01CA03, P01CB01, P01CB02, P01CC01, P01CC02, P01CD01, P01CD02, P01CX01, P01CX02, P01CX03, P01CX04, P02BA01, P02BA02, P02BB01, P02BX01, P02BX02, P02BX03, P02BX04, P02CA01, P02CA02, P02CA03, P02CA04, P02CA05, P02CA06, P02CA51, P02CB01, P02CB02, P02CC01, P02CC02, P02CE01, P02CF01, P02CX01, P02CX02, P02CX03, P02DA01, P02DX01, P02DX02, P03AA01, P03AA02, P03AA03, P03AA04, P03AA05, P03AA54, P03AB01, P03AB02, P03AB51, P03AC01, P03AC02, P03AC03, P03AC04, P03AC51, P03AC52, P03AC53, P03AC54, P03AX01, P03AX02, P03AX03, P03AX04, P03AX05, P03BA01, P03BA02, P03BA03, P03BA04, P03BX01, P03BX02, P03BX03, P03BX04, P03BX05, P03BX06
**Insulin and other antidiabetics**	A10AB01, A10AB02, A10AB03, A10AB04, A10AB05, A10AB06, A10AB30, A10AC01, A10AC02, A10AC03, A10AC04, A10AC30, A10AD01, A10AD02, A10AD03, A10AD04, A10AD05, A10AD06, A10AD30, A10AE01, A10AE02, A10AE03, A10AE04, A10AE05, A10AE06, A10AE30, A10AE54, A10AE56, A10AF01, A10BA01, A10BA02, A10BA03, A10BB01, A10BB02, A10BB03, A10BB04, A10BB05, A10BB06, A10BB07, A10BB08, A10BB09, A10BB10, A10BB11, A10BB12, A10BB31, A10BC01, A10BD01, A10BD02, A10BD03, A10BD04, A10BD05, A10BD06, A10BD07, A10BD08, A10BD09, A10BD10, A10BD11, A10BD12, A10BD13, A10BD14, A10BD15, A10BD16, A10BD17, A10BD18,A10BD19, A10BD20, A10BD21, A10BD22, A10BD23, A10BD24, A10BD25, A10BD26, A10BF01, A10BF02, A10BF03, A10BG01, A10BG02, A10BG03, A10BG04, A10BH01, A10BH02, A10BH03, A10BH04, A10BH05, A10BH06, A10BH07, A10BH08, A10BH52, A10BJ01, A10BJ02, A10BJ03, A10BJ04, A10BJ05, A10BJ06, A10BK01, A10BK02, A10BK03, A10BK04, A10BK05, A10BK06, A10BK07, A10BX01, A10BX02, A10BX03, A10BX04, A10BX05, A10BX06, A10BX07, A10BX08, A10BX09, A10BX10, A10BX11, A10BX12, A10BX13, A10BX14, A10XA01
**Heart drugs**	C01AA01, C01AA02, C01AA03, C01AA04, C01AA05, C01AA06, C01AA07, C01AA08,C01AA09, C01AA52, C01AB01, C01AB51, C01AC01, C01AC03, C01AX02, C01BA01, C01BA02, C01BA03, C01BA04, C01BA05, C01BA08, C01BA12, C01BA51, C01BA71, C01BB01, C01BB02, C01BB03, C01BB04, C01BC03, C01BC04, C01BC07, C01BC08, C01BD01, C01BD02, C01BD03, C01BD04, C01BD05, C01BD06, C01BD07, C01BG01, C01BG07, C01BG11, C01CA01, C01CA02, C01CA03, C01CA04, C01CA05, C01CA06, C01CA07, C01CA08, C01CA09, C01CA10, C01CA11, C01CA12, C01CA13, C01CA14, C01CA15, C01CA16, C01CA17, C01CA18, C01CA19, C01CA21, C01CA22, C01CA23, C01CA24, C01CA25, C01CA26, C01CA30, C01CA51, C01CE01, C01CE02, C01CE03, C01CE04, C01CX06, C01CX07, C01CX08, C01CX09, C01DA02, C01DA04, C01DA05, C01DA07, C01DA08, C01DA09, C01DA13, C01DA14, C01DA20, C01DA38, C01DA52, C01DA54, C01DA55, C01DA57, C01DA58, C01DA59, C01DA63, C01DA70, C01DB01, C01DX01, C01DX02, C01DX03, C01DX04, C01DX05, C01DX06, C01DX07, C01DX08, C01DX09, C01DX10, C01DX11, C01DX12, C01DX13, C01DX14, C01DX15, C01DX16, C01DX18, C01DX19, C01DX51, C01DX52, C01DX53, C01DX54, C01EA01, C01EB02, C01EB03, C01EB04, C01EB05, C01EB06, C01EB07, C01EB09, C01EB10, C01EB11, C01EB12, C01EB13, C01EB15, C01EB16, C01EB17, C01EB18, C01EB19, C01EB21
**Antihypertensives, including diuretics and renin-angiotensin-**	C02AA01, C02AA02, C02AA03, C02AA04, C02AA05, C02AA06, C02AA07, C02AA52, C02AA53, C02AA57, C02AB01, C02AB02, C02AC01, C02AC02, C02AC04, C02AC05,

**aldosterone system inhibitors**	C02AC06, C02BA01, C02BB01, C02CA01, C02CA02, C02CA03, C02CA04, C02CA06, C02CA08, C02CC01, C02CC02, C02CC03, C02CC04, C02CC05, C02CC06, C02CC07, C02DA01, C02DB01, C02DB02, C02DB03, C02DB04, C02DC01, C02DD01, C02DG01, C02KA01, C02KB01, C02KC01, C02KD01, C02KX01, C02KX02, C02KX03, C02KX04, C02KX05, C02KX52, C02LA01, C02LA02, C02LA03, C02LA04, C02LA07, C02LA08, C02LA09, C02LA50, C02LA51, C02LA52, C02LA71, C02LB01, C02LC01, C02LC05, C02LC51, C02LE01, C02LF01, C02LG01, C02LG02, C02LG03, C02LG51, C02LG73, C02LK01, C02LL01, C02LX01, C03AA01, C03AA02, C03AA03, C03AA04, C03AA05, C03AA06, C03AA07, C03AA08, C03AA09, C03AA13, C03AB01, C03AB02, C03AB03, C03AB04, C03AB05, C03AB06, C03AB07, C03AB08, C03AB09, C03AH01, C03AH02, C03AX01, C03BA02, C03BA03, C03BA04, C03BA05, C03BA07, C03BA08, C03BA09, C03BA10, C03BA11, C03BA12, C03BA13, C03BA82, C03BB02, C03BB03, C03BB04, C03BB05, C03BB07, C03BC01, C03BD01, C03BX03, C03CA01, C03CA02, C03CA03, C03CA04, C03CB01, C03CB02, C03CC01, C03CC02, C03CD01, C03CX01, C03DA01, C03DA02, C03DA03, C03DA04, C03DB01, C03DB02, C03EA01, C03EA02, C03EA03, C03EA04, C03EA05, C03EA06, C03EA07, C03EA12, C03EA13, C03EA14, C03EB01, C03EB02, C08CA01, C08CA02, C08CA03, C08CA04, C08CA05, C08CA06, C08CA07, C08CA08, C08CA09, C08CA10, C08CA11, C08CA12, C08CA13, C08CA14, C08CA15, C08CA16, C08CA51, C08CA55, C08CX01, C08DA01, C08DA02, C08DA51, C08DB01, C08EA01, C08EA02, C08EX01, C08EX02, C08GA01, C08GA02, C09AA01, C09AA02, C09AA03, C09AA04, C09AA05, C09AA06, C09AA07, C09AA08, C09AA09, C09AA10, C09AA11, C09AA12, C09AA13, C09AA14, C09AA15, C09AA16, C09BA01, C09BA02, C09BA03, C09BA04, C09BA05, C09BA06, C09BA07, C09BA08, C09BA09, C09BA12, C09BA13, C09BA15, C09BB02, C09BB03, C09BB04, C09BB05, C09BB06, C09BB07, C09BB10, C09BB12, C09BX01, C09BX03, C09BX04, C09BX05, C09CA01, C09CA02, C09CA03, C09CA04, C09CA05, C09CA06, C09CA07, C09CA08, C09CA09, C09DA01, C09DA02, C09DA03, C09DA04, C09DA06, C09DA07, C09DA08, C09DA10, C09DB01, C09DB02, C09DB04, C09DB05, C09DB06, C09DB07, C09DB09, C09DX01, C09DX02, C09DX03, C09DX04, C09DX05, C09DX06, C09DX07, C09XA01, C09XA02, C09XA52, C09XA53, C09XA54
**Beta-blockers**	C07AA01, C07AA02, C07AA03, C07AA05, C07AA06, C07AA07, C07AA12, C07AA14, C07AA15, C07AA16, C07AA17, C07AA19, C07AA23, C07AA27, C07AA57, C07AB01, C07AB02, C07AB03, C07AB04, C07AB05, C07AB06, C07AB07, C07AB08, C07AB09, C07AB10, C07AB11, C07AB12, C07AB13, C07AB14, C07AB52, C07AG01, C07AG02, C07BA02, C07BA05, C07BA06, C07BA07, C07BA12, C07BA68, C07BB02, C07BB03, C07BB04, C07BB06, C07BB07, C07BB12, C07BB52, C07BG01, C07CA02, C07CA03, C07CA17, C07CA23, C07CB02, C07CB03, C07CB53, C07CG01, C07DA06, C07DB01, C07FA05, C07FB02, C07FB03, C07FB07, C07FB13, C07FX01, C07FX02, C07FX03, C07FX04, C07FX05, C07FX06
**Statins, fibrates, including proprotein convertase subtilisin/kexin type 9 inhibitors and inclisiran**	C10AA01, C10AA02, C10AA03, C10AA04, C10AA05, C10AA06, C10AA07, C10AA08, C10AA51, C10AA52, C10AA53, C10AA55, C10AB01, C10AB02, C10AB03, C10AB04, C10AB05, C10AB06, C10AB07, C10AB08, C10AB09, C10AB10, C10AB11, C10AX06, C10AX07, C10AX08, C10AX09, C10AX10, C10AX11, C10AX12, C10AX13, C10AX14, C10AX15, C10AX16, C10BA01, C10BA02, C10BA03, C10BA04, C10BA05, C10BA06, C10BA07, C10BA08, C10BA09, C10BA10, C10BX01, C10BX02, C10BX03, C10BX04, C10BX05, C10BX06, C10BX07, C10BX08, C10BX09, C10BX10, C10BX11, C10BX12, C10BX13, C10BX14, C10BX15, C10BX16, C10BX17, C10BX18
**Immunosuppressants and immunomodulators**	D11AH05, L04AA01, L04AA02, L04AA03, L04AA04, L04AA05, L04AA06, L04AA08, L04AA09, L04AA10, L04AA11, L04AA12, L04AA13, L04AA14, L04AA15, L04AA16, L04AA17, L04AA18, L04AA19, L04AA21, L04AA22, L04AA23, L04AA24, L04AA25, L04AA26, L04AA27, L04AA28, L04AA29, L04AA31, L04AA32, L04AA33, L04AA34, L04AA36, L04AA37, L04AA38, L04AA39, L04AA40, L04AA41, L04AA42, L04AA43,

	L04AA44, L04AA45, L04AA46, L04AB01, L04AB02, L04AB03, L04AB04, L04AB05, L04AB06, L04AB07, L04AC01, L04AC02, L04AC03, L04AC04, L04AC05, L04AC06, L04AC07, L04AC08, L04AC09, L04AC10, L04AC11, L04AC12, L04AC13, L04AC14, L04AC15, L04AC16, L04AC17, L04AC18, L04AC19, L04AD01, L04AD02, L04AD03, L04AX01, L04AX02, L04AX03, L04AX04, L04AX05, L04AX06, L04AX07, L04AX08
**Systemic steroids**	H02AA01, H02AA02, H02AA03, H02AB01, H02AB02, H02AB03, H02AB04, H02AB05, H02AB06, H02AB07, H02AB08, H02AB09, H02AB10, H02AB11, H02AB12, H02AB13, H02AB14, H02AB15, H02AB17, H02BX01, H02CA01, H02CA02, H02CA03
**Chemotherapy**	L01AA01, L01AA02, L01AA03, L01AA05, L01AA06, L01AA07, L01AA08, L01AA09, L01AB01, L01AB02, L01AB03, L01AC01, L01AC02, L01AC03, L01AD01, L01AD02, L01AD03, L01AD04, L01AD05, L01AD06, L01AD07, L01AD08, L01AG01, L01AX01, L01AX02, L01AX03, L01AX04, L01BA01, L01BA03, L01BA04, L01BB02, L01BB03, L01BB04, L01BB05, L01BB06, L01BB07, L01BC01, L01BC02, L01BC03, L01BC04, L01BC05, L01BC06, L01BC07, L01BC08, L01BC09, L01BC52, L01BC53, L01BC59, L01CA01, L01CA02, L01CA03, L01CA04, L01CA05, L01CB01, L01CB02, L01CC01, L01CD01, L01CD02, L01CD03, L01CD04, L01CE01, L01CE02, L01CE03, L01CE04, L01CX01, L01DA01, L01DB01, L01DB02, L01DB03, L01DB04, L01DB05, L01DB06, L01DB07, L01DB08, L01DB09, L01DB10, L01DB11, L01DC01, L01DC02, L01DC03, L01DC04, L01EA01, L01EA02, L01EA03, L01EA04, L01EA05, L01EB01, L01EB02, L01EB03, L01EB04, L01EB05, L01EB07, L01EB08, L01EC01, L01EC02, L01EC03, L01ED01, L01ED02, L01ED03, L01ED04, L01ED05, L01EE01, L01EE02, L01EE03, L01EF01, L01EF02, L01EF03, L01EG01, L01EG02, L01EH01, L01EH02, L01EJ01, L01EJ02, L01EK01, L01EK03, L01EL01, L01EL02, L01EM01, L01EM02, L01EM03, L01EX01, L01EX02, L01EX03, L01EX04, L01EX05, L01EX07, L01EX08, L01EX09, L01EX10, L01EX11, L01EX12, L01EX13, L01EX14, L01XA01, L01XA02, L01XA03, L01XA04, L01XA05, L01XB01, L01XC01, L01XC02, L01XC03, L01XC04, L01XC05, L01XC06, L01XC07, L01XC08, L01XC09, L01XC10, L01XC11, L01XC12, L01XC13, L01XC14, L01XC15, L01XC16, L01XC17, L01XC18, L01XC19, L01XC21, L01XC22, L01XC23, L01XC24, L01XC25, L01XC26, L01XC27, L01XC28, L01XC29, L01XC31, L01XC32, L01XC33, L01XC34, L01XC35, L01XC36, L01XC37, L01XC38, L01XC39, L01XC40, L01XC41, L01XD01, L01XD02, L01XD03, L01XD04, L01XD05, L01XD06, L01XD07, L01XE01, L01XE02, L01XE03, L01XE04, L01XE05, L01XE06, L01XE07, L01XE08, L01XE09, L01XE10, L01XE11, L01XE12, L01XE13, L01XE14, L01XE15, L01XE16, L01XE17, L01XE18, L01XE21, L01XE23, L01XE24, L01XE25, L01XE26, L01XE27, L01XE28, L01XE29, L01XE31, L01XE33, L01XE34, L01XE35, L01XE36, L01XE37, L01XE38, L01XE39, L01XE41, L01XE42, L01XE43, L01XE44, L01XE45, L01XE46, L01XE47, L01XE48, L01XE50, L01XE51, L01XE52, L01XE53, L01XE54, L01XE56, L01XE57, L01XF01, L01XF02, L01XF03, L01XG01, L01XG02, L01XG03, L01XH01, L01XH02, L01XH03, L01XH05, L01XJ01, L01XJ02, L01XJ03, L01XK01, L01XK02, L01XK03, L01XK04, L01XX01, L01XX02, L01XX03, L01XX05, L01XX07, L01XX08, L01XX09, L01XX10, L01XX11, L01XX14, L01XX16, L01XX17, L01XX18, L01XX19, L01XX22, L01XX23, L01XX24, L01XX25, L01XX27, L01XX28, L01XX29, L01XX31, L01XX32, L01XX33, L01XX34, L01XX35, L01XX36, L01XX37, L01XX38, L01XX39, L01XX40, L01XX41, L01XX42, L01XX43, L01XX44, L01XX45, L01XX46, L01XX47, L01XX48, L01XX50, L01XX51, L01XX52, L01XX53, L01XX54, L01XX55, L01XX56, L01XX57, L01XX58, L01XX59, L01XX60, L01XX61, L01XX62, L01XX63, L01XX64, L01XX65, L01XX66, L01XX67, L01XX68, L01XX70, L01XX71, L01XY01, L01XY02, L02AA01, L02AA02, L02AA03, L02AA04, L02AB01, L02AB02, L02AB03, L02AE01, L02AE02, L02AE03, L02AE04, L02AE05, L02AE51, L02BA01, L02BA02, L02BA03, L02BB01, L02BB02, L02BB03, L02BB04, L02BB05, L02BB06, L02BG01, L02BG02, L02BG03, L02BG04, L02BG05, L02BG06, L02BX01, L02BX02, L02BX03
**Iron supplements,**	B03AA01, B03AA02, B03AA03, B03AA04, B03AA05, B03AA06, B03AA07, B03AA08,

**erythropoietic stimulating agents, vitamin B12, folic acid**	B03AA09, B03AA10, B03AA11, B03AA12, B03AB01, B03AB02, B03AB03, B03AB04, B03AB05, B03AB06, B03AB07, B03AB08, B03AB09, B03AB10, B03AC01, B03AC02, B03AC03, B03AC05, B03AC06, B03AC07, B03AD01, B03AD02, B03AD03, B03AD04, B03AD05, B03AE01, B03AE02, B03AE03, B03AE04, B03AE10, B03BA01, B03BA02, B03BA03, B03BA04, B03BA05, B03BA51, B03BA53, B03BB01, B03BB51, B03XA01, B03XA02, B03XA03, B03XA05, B03XA06
**Antacids, including antihistamines**	A02BB01, A02BB02, A02BC01, A02BC02, A02BC03, A02BC04, A02BC05, A02BC06, A02BC08
**Vitamin D and other vitamin supplements**	A11CA01, A11CA02, A11CC01, A11CC02, A11CC03, A11CC04, A11CC05, A11CC06, A11CC07, A11CC20, A11CC55, A11DA01, A11DA02, A11DA03, A11GA01, A11GB01, A11HA01, A11HA02, A11HA03, A11HA04, A11HA05, A11HA06, A11HA07, A11HA08, A11HA30, A11HA31, A11HA32
**Caplacizumab**	B01AX07
**Systemic hemostatics**	B02BX01, B02BX02, B02BX03, B02BX04, B02BX05, B02BX06, B02BX07, B02BX08, B02BX09
**Hereditary angioedema therapeutics**	B06AC01, B06AC02, B06AC03, B06AC04, B06AC05
**Peripheral vasodilators**	C04AA01, C04AA02, C04AA31, C04AB01, C04AB02, C04AC01, C04AC02, C04AC03, C04AC07, C04AD01, C04AD02, C04AD03, C04AD04, C04AE01, C04AE02, C04AE04, C04AE51, C04AE54, C04AF01, C04AX01, C04AX02, C04AX07, C04AX10, C04AX11, C04AX13, C04AX17, C04AX19, C04AX20, C04AX21, C04AX23, C04AX24, C04AX26, C04AX27, C04AX28, C04AX30, C04AX32
**Hormonal contraceptives and similar hormone preparations**	G03AA01, G03AA02, G03AA03, G03AA04, G03AA05, G03AA06, G03AA07, G03AA08, G03AA09, G03AA10, G03AA11, G03AA12, G03AA13, G03AA14, G03AA15, G03AA16, G03AA17, G03AB01, G03AB02, G03AB03, G03AB04, G03AB05, G03AB06, G03AB07, G03AB08, G03AB09, G03AC01, G03AC02, G03AC03, G03AC04, G03AC05, G03AC06, G03AC07, G03AC08, G03AC09, G03AC10, G03AD01, G03AD02, G03BA01, G03BA02, G03BA03, G03BB01, G03BB02, G03CA01, G03CA03, G03CA04, G03CA06, G03CA07, G03CA09, G03CA53, G03CA57, G03CB01, G03CB02, G03CB03, G03CB04, G03CC02, G03CC03, G03CC04, G03CC05, G03CC06, G03CC07, G03CX01, G03DA01, G03DA02, G03DA03, G03DA04, G03DB01, G03DB02, G03DB03, G03DB04, G03DB05, G03DB06, G03DB07, G03DB08, G03DC01, G03DC02, G03DC03, G03DC04, G03DC05, G03DC06, G03DC31, G03EA01, G03EA02, G03EA03, G03EK01, G03FA01, G03FA02, G03FA03, G03FA04, G03FA05, G03FA06, G03FA07, G03FA08, G03FA09, G03FA10, G03FA11, G03FA12, G03FA13, G03FA14, G03FA15, G03FA16, G03FA17, G03FB01, G03FB02, G03FB03, G03FB04, G03FB05, G03FB06, G03FB07, G03FB08, G03FB09, G03FB10, G03FB11, G03GA01, G03GA02, G03GA03, G03GA04, G03GA05, G03GA06, G03GA07, G03GA08, G03GA09, G03GA10, G03GA30, G03GB01, G03GB02, G03GB03, G03HA01, G03HB01, G03XA01, G03XA02, G03XB01, G03XB02, G03XC01, G03XC02, G03XC03, G03XX01
**Immunoglobulins**	J06BA01, J06BA02
**Interferons and CSF**	L03AA02, L03AA03, L03AA09, L03AA10, L03AA12, L03AA13, L03AA14, L03AA16, L03AA17, L03AB01, L03AB02, L03AB03, L03AB04, L03AB05, L03AB06, L03AB07, L03AB08, L03AB09, L03AB10, L03AB11, L03AB12, L03AB13, L03AB15, L03AB60, L03AB61, L03AC01, L03AC02, L03AX01, L03AX02, L03AX03, L03AX04, L03AX05, L03AX07, L03AX08, L03AX09, L03AX10, L03AX11, L03AX12, L03AX13, L03AX14, L03AX15, L03AX16, L03AX17, L03AX21
**NSAID and other anti-inflammatory drugs**	M01AA01, M01AA02, M01AA03, M01AA05, M01AA06, M01AB01, M01AB02, M01AB03, M01AB04, M01AB05, M01AB06, M01AB07, M01AB08, M01AB09, M01AB10, M01AB11, M01AB12, M01AB13, M01AB14, M01AB15, M01AB16, M01AB17, M01AB51, M01AB55,

	M01AC01, M01AC02, M01AC04, M01AC05, M01AC06, M01AC56, M01AE01, M01AE02, M01AE03, M01AE04, M01AE05, M01AE06, M01AE07, M01AE08, M01AE09, M01AE10, M01AE11, M01AE12, M01AE13, M01AE14, M01AE15, M01AE16, M01AE17, M01AE18, M01AE51, M01AE52, M01AE53, M01AE56, M01AG01, M01AG02, M01AG03, M01AG04, M01AH01, M01AH02, M01AH03, M01AH04, M01AH05, M01AH06, M01AH07, M01AX01, M01AX02, M01AX04, M01AX05, M01AX07, M01AX12, M01AX13, M01AX14, M01AX17, M01AX18, M01AX21, M01AX22, M01AX23, M01AX24, M01AX25, M01AX26, M01AX68, M01BA01, M01BA02, M01BA03, M01CA03, M01CB01, M01CB02, M01CB03, M01CB04, M01CB05, M01CC01, M01CC02
**Gout medications**	M04AA01, M04AA02, M04AA03, M04AA51, M04AB01, M04AB02, M04AB03, M04AB04, M04AB05, M04AC01, M04AC02, M04AX01
**Antiepileptics**	N03AA01, N03AA02, N03AA03, N03AA04, N03AA30, N03AB01, N03AB02, N03AB03, N03AB04, N03AB05, N03AB52, N03AB54, N03AC01, N03AC02, N03AC03, N03AD01, N03AD02, N03AD03, N03AD51, N03AE01, N03AF01, N03AF02, N03AF03, N03AF04, N03AG01, N03AG02, N03AG03, N03AG04, N03AG05, N03AG06, N03AX03, N03AX07, N03AX09, N03AX10, N03AX11, N03AX12, N03AX13, N03AX14, N03AX15, N03AX16, N03AX17, N03AX18, N03AX21, N03AX22, N03AX23, N03AX24, N03AX30
**Antipsychotics/Antidepressive Drugs**	N05AA01, N05AA02, N05AA03, N05AA04, N05AA05, N05AA06, N05AA07, N05AB01, N05AB02, N05AB03, N05AB04, N05AB05, N05AB06, N05AB07, N05AB08, N05AB09, N05AB10, N05AC01, N05AC02, N05AC03, N05AC04, N05AD01, N05AD02, N05AD03, N05AD04, N05AD05, N05AD06, N05AD07, N05AD08, N05AD09, N05AE01, N05AE02, N05AE03, N05AE04, N05AF01, N05AF02, N05AF03, N05AF04, N05AF05, N05AG01, N05AG02, N05AG03, N05AH01, N05AH02, N05AH03, N05AH04, N05AH05, N05AH06, N05AK01, N05AL01, N05AL02, N05AL03, N05AL04, N05AL05, N05AL06, N05AL07, N05AN01, N05AX07, N05AX08, N05AX09, N05AX10, N05AX11, N05AX12, N05AX13, N05AX14, N05AX15, N05BA01, N05BA02, N05BA03, N05BA04, N05BA05, N05BA06, N05BA07, N05BA08, N05BA09, N05BA10, N05BA11, N05BA12, N05BA13, N05BA14, N05BA15, N05BA16, N05BA17, N05BA18, N05BA19, N05BA21, N05BA22, N05BA23, N05BA56, N05BB01, N05BB02, N05BB51, N05BC01, N05BC03, N05BC04, N05BC51, N05BD01, N05BE01, N05BX01, N05BX02, N05BX03, N05BX05, N05CA01, N05CA02, N05CA03, N05CA04, N05CA05, N05CA06, N05CA07, N05CA08, N05CA09, N05CA10, N05CA11, N05CA12, N05CA15, N05CA16, N05CA19, N05CA20, N05CA21, N05CA22, N05CB01, N05CB02, N05CC01, N05CC02, N05CC03, N05CC04, N05CC05, N05CD01, N05CD02, N05CD03, N05CD04, N05CD05, N05CD06, N05CD07, N05CD08, N05CD09, N05CD10, N05CD11, N05CD12, N05CD13, N05CD14, N05CD15, N05CE01, N05CE02, N05CE03, N05CF01, N05CF02, N05CF03, N05CF04, N05CH01, N05CH02, N05CM01, N05CM02, N05CM03, N05CM04, N05CM05, N05CM06, N05CM07, N05CM08, N05CM09, N05CM10, N05CM11, N05CM12, N05CM13, N05CM15, N05CM16, N05CM17, N05CM18, N05CM19, N05CX01, N05CX02, N05CX03, N05CX04, N05CX05, N05CX06, N06AA01, N06AA02, N06AA03, N06AA04, N06AA05, N06AA06, N06AA07, N06AA08, N06AA09, N06AA10, N06AA11, N06AA12, N06AA13, N06AA14, N06AA15, N06AA16, N06AA17, N06AA18, N06AA19, N06AA21, N06AA23, N06AB02, N06AB03, N06AB04, N06AB05, N06AB06, N06AB07, N06AB08, N06AB09, N06AB10, N06AF01, N06AF02, N06AF03, N06AF04, N06AF05, N06AF06, N06AG02, N06AG03, N06AX01, N06AX02, N06AX03, N06AX04, N06AX05, N06AX06, N06AX07, N06AX08, N06AX09, N06AX10, N06AX11, N06AX12, N06AX13, N06AX14, N06AX15, N06AX16, N06AX17, N06AX18, N06AX19, N06AX21, N06AX22, N06AX23, N06AX24, N06AX25, N06AX26, N06AX27, N06BA01, N06BA02, N06BA03, N06BA04, N06BA05, N06BA06, N06BA07, N06BA08, N06BA09, N06BA10, N06BA11, N06BA12, N06BA14, N06BC01, N06BC02, N06BX01, N06BX02, N06BX03, N06BX04, N06BX05, N06BX06, N06BX07, N06BX08, N06BX09, N06BX10, N06BX11, N06BX12, N06BX13, N06BX14, N06BX15, N06BX16, N06BX17, N06BX18, N06BX21, N06CA01, N06CA02, N06DA01, N06DA02, N06DA03, N06DA04, N06DA52,

	N06DX01, N06DX02
**Rhinological and throat antiseptics**	R01AA02, R01AA03, R01AA04, R01AA05, R01AA06, R01AA07, R01AA08, R01AA09, R01AA10, R01AA11, R01AA12, R01AA13, R01AA14, R01AB01, R01AB02, R01AB03, R01AB05, R01AB06, R01AB07, R01AB08, R01AC01, R01AC02, R01AC03, R01AC04, R01AC05, R01AC06, R01AC07, R01AC08, R01AC51, R01AD01, R01AD02, R01AD03, R01AD04, R01AD05, R01AD06, R01AD07, R01AD08, R01AD09, R01AD11, R01AD12, R01AD13, R01AD52, R01AD53, R01AD57, R01AD58, R01AD60, R01AX01, R01AX02, R01AX03, R01AX05, R01AX06, R01AX07, R01AX08, R01AX09, R01AX10, R01AX30, R01BA01, R01BA02, R01BA03, R01BA51, R01BA52, R01BA53, R02AA01, R02AA02, R02AA03, R02AA05, R02AA06, R02AA09, R02AA10, R02AA11, R02AA12, R02AA13, R02AA14, R02AA15, R02AA16, R02AA17, R02AA18, R02AA19, R02AA20, R02AB01, R02AB02, R02AB03, R02AB04, R02AB30, R02AD01, R02AD02, R02AD03, R02AD04, R02AX01, R02AX03
**Inhaled anti-obstructive drugs**	R03AA01, R03AB02, R03AB03, R03AC02, R03AC03, R03AC04, R03AC05, R03AC06, R03AC07, R03AC08, R03AC09, R03AC10, R03AC11, R03AC12, R03AC13, R03AC14, R03AC15, R03AC16, R03AC17, R03AC18, R03AC19, R03AK01, R03AK02, R03AK03, R03AK04, R03AK05, R03AK06, R03AK07, R03AK08, R03AK09, R03AK10, R03AK11, R03AK13, R03AK14, R03AL01, R03AL02, R03AL03, R03AL04, R03AL05, R03AL06, R03AL07, R03AL08, R03AL09, R03AL10, R03AL11, R03AL12, R03BB01, R03BB02, R03BB03, R03BB04, R03BB05, R03BB06, R03BB07, R03BB08, R03BC01, R03BC03, R03BX01, R03CA02, R03CB01, R03CB02, R03CB03, R03CB51, R03CB53, R03CC02, R03CC03, R03CC04, R03CC05, R03CC06, R03CC07, R03CC08, R03CC09, R03CC10, R03CC11, R03CC12, R03CC13, R03CC14, R03CC53, R03CC63, R03DA01, R03DA02, R03DA03, R03DA04, R03DA05, R03DA06, R03DA07, R03DA08, R03DA09, R03DA10, R03DA11, R03DA12, R03DA20, R03DA51, R03DA54, R03DA55, R03DA57, R03DA74, R03DB01, R03DB02, R03DB03, R03DB04, R03DB05, R03DB06
**Inhaled steroids**	R03BA01, R03BA02, R03BA03, R03BA04, R03BA05, R03BA06, R03BA07, R03BA08, R03BA09
**Other COPD drugs**	R03DC01, R03DC02, R03DC03, R03DC04, R03DX01, R03DX02, R03DX03, R03DX05, R03DX06, R03DX07, R03DX08, R03DX09, R03DX10
**Cold and cough preparations**	R05CA01, R05CA02, R05CA03, R05CA04, R05CA05, R05CA06, R05CA07, R05CA08, R05CA09, R05CA10, R05CA11, R05CA12, R05CA13, R05CB01, R05CB02, R05CB03, R05CB04, R05CB05, R05CB06, R05CB07, R05CB08, R05CB09, R05CB10, R05CB11, R05CB12, R05CB13, R05CB14, R05CB15, R05CB16, R05DA01, R05DA03, R05DA04, R05DA05, R05DA06, R05DA07, R05DA08, R05DA09, R05DA10, R05DA11, R05DA12, R05DA20, R05DB01, R05DB02, R05DB03, R05DB04, R05DB05, R05DB07, R05DB09, R05DB10, R05DB11, R05DB12, R05DB13, R05DB14, R05DB15, R05DB16, R05DB17, R05DB18, R05DB19, R05DB20, R05DB21, R05DB22, R05DB23, R05DB24, R05DB25, R05DB26, R05DB27, R05DB28, R05FA01, R05FA02, R05FB01, R05FB02
**Systemic antihistamines**	R06AA01, R06AA02, R06AA04, R06AA06, R06AA07, R06AA08, R06AA09, R06AA52, R06AA54, R06AA56, R06AA57, R06AA59, R06AB01, R06AB02, R06AB03, R06AB04, R06AB05, R06AB06, R06AB07, R06AB51, R06AB52, R06AB54, R06AB56, R06AC01, R06AC02, R06AC03, R06AC04, R06AC05, R06AC06, R06AC52, R06AC53, R06AD01, R06AD02, R06AD03, R06AD04, R06AD05, R06AD06, R06AD07, R06AD08, R06AD09, R06AD52, R06AD55, R06AE01, R06AE03, R06AE04, R06AE05, R06AE06, R06AE07, R06AE09, R06AE51, R06AE53, R06AE55, R06AX01, R06AX02, R06AX03, R06AX04, R06AX05, R06AX07, R06AX08, R06AX09, R06AX11, R06AX12, R06AX13, R06AX15, R06AX16, R06AX17, R06AX18, R06AX19, R06AX21, R06AX22, R06AX23, R06AX24, R06AX25, R06AX26, R06AX27, R06AX28, R06AX29, R06AX53, R06AX58

Medication Profiles

Tables S4 to S7 show the numbers and percentages of patients receiving at least one medication of the corresponding medication group in the year before index hospitalization, separately for COVID-hospitalized patients as compared to their respectively age-, sex- and region- matched controls for the four different age groups.

Tables S8 to S11 show the numbers and percentages of patients receiving at least one medication of the corresponding medication group in the year before index hospitalization, separately for Influenza-hospitalized patients as compared to their respectively age-, sex- and region- matched controls for the four different age groups.

Tables S12 to S15 show the numbers and percentages of patients receiving at least one medication of the corresponding medication group in the year before index hospitalization, separately for COVID-hospitalized and Influenza patients before and after propensity score matching.

Table S4: Medication Groups for COVID-19 hospitalized patients aged 19-40 as compared to controls

Age Group 19-40	COVID (n = 3941)		Control (n = 39214)		
**Medication Groups**	**No: n (%)**	**Yes: n (%)**	**No: n (%)**	**Yes: n (%)**	**p-value**
**MG 1: Anticoagulants**	3620 (91.85)	321 (8.15)	38469 (98.1)	745 (1.9)	<0.001
**MG 2: Antibiotics, Antivirals, Antiprotozoals or Anthelmintics**	2271 (57.62)	1670 (42.38)	32160 (82.01)	7054 (17.99)	<0.001
**MG 3: Insulins and other Antidiabetics**	3836 (97.34)	105 (2.66)	39042 (99.56)	172 (0.44)	<0.001
**MG 4: "heart" drugs**	3879 (98.43)	62 (1.57)	39018 (99.5)	196 (0.5)	<0.001
**MG 5: Antihypertensives incl. Diuretics and Renin-angiotensin-aldosterone system inhibitors**	3712 (94.19)	229 (5.81)	38893 (99.18)	321 (0.82)	<0.001
**MG 6: Beta Blockers**	3848 (97.64)	93 (2.36)	39101 (99.71)	113 (0.29)	<0.001
**MG 7: Statins, Fibrates incl. Proprotein convertase subtilisin/kexin type 9 inhibitors and Inclisiran**	3845 (97.56)	96 (2.44)	39033 (99.54)	181 (0.46)	<0.001
**MG 8: Immunosuppressants and Immunomodulators**	3837 (97.36)	104 (2.64)	39063 (99.61)	151 (0.39)	<0.001
**MG 9: Systemic Steroids**	3676 (93.28)	265 (6.72)	38639 (98.53)	575 (1.47)	<0.001
**MG 10: Chemotherapy**	3922 (99.52)	19 (0.48)	39191 (99.94)	23 (0.06)	<0.001
**MG 11: Iron supplements, Erythropoietic stimulating agents, Vitamin B12, folic acid**	3700 (93.88)	241 (6.12)	38653 (98.57)	561 (1.43)	<0.001
**MG 12: Antacids incl. Antihistamines**	3563 (90.41)	378 (9.59)	38483 (98.14)	731 (1.86)	<0.001
**MG 13: Vitamin D and other Vitamin supplements**	3664 (92.97)	277 (7.03)	38660 (98.59)	554 (1.41)	<0.001
**MG 14: Caplacizumab**	3940 (99.97)	1 (0.03)	39214 (100)	0 (0)	0.0913
**MG 15: Systemic Hemostatics**	3940 (99.97)	1 (0.03)	39212 (99.99)	2 (0.01)	0.2497
**MG 16: Hereditary angioedema Therapeutics**	3940 (99.97)	1 (0.03)	39214 (100)	0 (0)	0.0913
**MG 17: Peripheral Vasodilators**	3936 (99.87)	5 (0.13)	39197 (99.96)	17 (0.04)	0.0652
**MG 18: Hormonal contraceptives and similar hormone preparations**	3752 (95.2)	189 (4.8)	38565 (98.34)	649 (1.66)	<0.001
**MG 19: Immunoglobulins**	3937 (99.9)	4 (0.1)	39211 (99.99)	3 (0.01)	0.0019
**MG 20: Interferons and CSF**	3893 (98.78)	48 (1.22)	39051 (99.58)	163 (0.42)	<0.001
**MG 21: NSAR and other anti-inflammatory drugs**	3010 (76.38)	931 (23.62)	35716 (91.08)	3498 (8.92)	<0.001
**MG 22: Gout medications**	3922 (99.52)	19 (0.48)	39190 (99.94)	24 (0.06)	<0.001
**MG 23: Antiepileptics**	3770 (95.66)	171 (4.34)	38927 (99.27)	287 (0.73)	<0.001
**MG 24: Antipsychotics**	3446 (87.44)	495 (12.56)	37646 (96)	1568 (4)	<0.001
**MG 25: Rhinological and throat antiseptics**	3570 (90.59)	371 (9.41)	37719 (96.19)	1495 (3.81)	<0.001
**MG 26: inhaled anti-obstructive drugs**	3438 (87.24)	503 (12.76)	38206 (97.43)	1008 (2.57)	<0.001
**MG 27: inhaled steroids**	3746 (95.05)	195 (4.95)	38926 (99.27)	288 (0.73)	<0.001
**MG 28: other COPD drugs**	3904 (99.06)	37 (0.94)	39107 (99.73)	107 (0.27)	<0.001
**MG 29: Cold and Cough preparations**	3727 (94.57)	214 (5.43)	38829 (99.02)	385 (0.98)	<0.001
**MG 30: Systemic Antihistamines**	3762 (95.46)	179 (4.54)	38574 (98.37)	640 (1.63)	<0.001

Table S5: Medication Groups for COVID-19 hospitalized aged 41-64 patients as compared to controls

Age Group 41-64	COVID (n = 16481)		Control (n = 161718)		
**Medication Groups**	**No: n (%)**	**Yes: n (%)**	**No: n (%)**	**Yes: n (%)**	**p-value**
**MG 1: Anticoagulants**	13288 (80.63)	3193 (19.37)	153606 (94.98)	8112 (5.02)	<0.001
**MG 2: Antibiotics, Antivirals, Antiprotozoals or Anthelmintics**	9464 (57.42)	7017 (42.58)	133534 (82.57)	28184 (17.43)	<0.001
**MG 3: Insulins and other Antidiabetics**	14320 (86.89)	2161 (13.11)	157684 (97.51)	4034 (2.49)	<0.001
**MG 4: "heart" drugs**	15821 (96)	660 (4)	159506 (98.63)	2212 (1.37)	<0.001
**MG 5: Antihypertensives incl. Diuretics and Renin-angiotensin-aldosterone system inhibitors**	11493 (69.73)	4988 (30.27)	147550 (91.24)	14168 (8.76)	<0.001
**MG 6: Beta Blockers**	14397 (87.36)	2084 (12.64)	156399 (96.71)	5319 (3.29)	<0.001
**MG 7: Statins, Fibrates incl. Proprotein convertase subtilisin/kexin type 9 inhibitors and Inclisiran**	13112 (79.56)	3369 (20.44)	150911 (93.32)	10807 (6.68)	<0.001
**MG 8: Immunosuppressants and Immunomodulators**	15829 (96.04)	652 (3.96)	160476 (99.23)	1242 (0.77)	<0.001
**MG 9: Systemic Steroids**	14478 (87.85)	2003 (12.15)	155680 (96.27)	6038 (3.73)	<0.001
**MG 10: Chemotherapy**	16235 (98.51)	246 (1.49)	161092 (99.61)	626 (0.39)	<0.001
**MG 11: Iron supplements, Erythropoietic stimulating agents, Vitamin B12, folic acid**	15671 (95.09)	810 (4.91)	159994 (98.93)	1724 (1.07)	<0.001
**MG 12: Antacids incl. Antihistamines**	13462 (81.68)	3019 (18.32)	154696 (95.66)	7022 (4.34)	<0.001
**MG 13: Vitamin D and other Vitamin supplements**	14604 (88.61)	1877 (11.39)	156282 (96.64)	5436 (3.36)	<0.001
**MG 14: Caplacizumab**	16481 (100)	0 (0)	161717 (100)	1 (0)	1
**MG 15: Systemic Hemostatics**	16476 (99.97)	5 (0.03)	161710 (100)	8 (0)	0.0016
**MG 16: Hereditary angioedema Therapeutics**	16481 (100)	0 (0)	161717 (100)	1 (0)	1
**MG 17: Peripheral Vasodilators**	16412 (99.58)	69 (0.42)	161375 (99.79)	343 (0.21)	<0.001
**MG 18: Hormonal contraceptives and similar hormone preparations**	15922 (96.61)	559 (3.39)	157985 (97.69)	3733 (2.31)	<0.001
**MG 19: Immunoglobulins**	16471 (99.94)	10 (0.06)	161702 (99.99)	16 (0.01)	<0.001
**MG 20: Interferons and CSF**	16199 (98.29)	282 (1.71)	160833 (99.45)	885 (0.55)	<0.001
**MG 21: NSAR and other anti-inflammatory drugs**	10852 (65.85)	5629 (34.15)	138200 (85.46)	23518 (14.54)	<0.001
**MG 22: Gout medications**	16096 (97.66)	385 (2.34)	160762 (99.41)	956 (0.59)	<0.001
**MG 23: Antiepileptics**	15174 (92.07)	1307 (7.93)	158558 (98.05)	3160 (1.95)	<0.001
**MG 24: Antipsychotics**	12866 (78.07)	3615 (21.93)	149849 (92.66)	11869 (7.34)	<0.001
**MG 25: Rhinological and throat antiseptics**	15105 (91.65)	1376 (8.35)	156388 (96.7)	5330 (3.3)	<0.001
**MG 26: inhaled anti-obstructive drugs**	13354 (81.03)	3127 (18.97)	154577 (95.58)	7141 (4.42)	<0.001
**MG 27: inhaled steroids**	15490 (93.99)	991 (6.01)	159895 (98.87)	1823 (1.13)	<0.001
**MG 28: other COPD drugs**	16214 (98.38)	267 (1.62)	161117 (99.63)	601 (0.37)	<0.001
**MG 29: Cold and Cough preparations**	15222 (92.36)	1259 (7.64)	159398 (98.57)	2320 (1.43)	<0.001
**MG 30: Systemic Antihistamines**	15601 (94.66)	880 (5.34)	158944 (98.28)	2774 (1.72)	<0.001

Table S6: Medication Groups for COVID-19 hospitalized patients aged 65-74 as compared to controls

Age Group 65-74	COVID (n = 10140)		Control (n = 97946)		
**Medication Groups**	**No: n (%)**	**Yes: n (%)**	**No: n (%)**	**Yes: n (%)**	**p-value**
**MG 1: Anticoagulants**	6027 (59.44)	4113 (40.56)	84880 (86.66)	13066 (13.34)	<0.001
**MG 2: Antibiotics, Antivirals, Antiprotozoals or Anthelmintics**	5743 (56.64)	4397 (43.36)	76980 (78.59)	20966 (21.41)	<0.001
**MG 3: Insulins and other Antidiabetics**	7455 (73.52)	2685 (26.48)	90951 (92.86)	6995 (7.14)	<0.001
**MG 4: "heart" drugs**	9119 (89.93)	1021 (10.07)	93944 (95.91)	4002 (4.09)	<0.001
**MG 5: Antihypertensives incl. Diuretics and Renin-angiotensin-aldosterone system inhibitors**	4197 (41.39)	5943 (58.61)	75233 (76.81)	22713 (23.19)	<0.001
**MG 6: Beta Blockers**	7046 (69.49)	3094 (30.51)	87647 (89.49)	10299 (10.51)	<0.001
**MG 7: Statins, Fibrates incl. Proprotein convertase subtilisin/kexin type 9 inhibitors and Inclisiran**	5897 (58.16)	4243 (41.84)	79766 (81.44)	18180 (18.56)	<0.001
**MG 8: Immunosuppressants and Immunomodulators**	9655 (95.22)	485 (4.78)	96800 (98.83)	1146 (1.17)	<0.001
**MG 9: Systemic Steroids**	8410 (82.94)	1730 (17.06)	91510 (93.43)	6436 (6.57)	<0.001
**MG 10: Chemotherapy**	9770 (96.35)	370 (3.65)	96672 (98.7)	1274 (1.3)	<0.001
**MG 11: Iron supplements, Erythropoietic stimulating agents, Vitamin B12, folic acid**	9255 (91.27)	885 (8.73)	95951 (97.96)	1995 (2.04)	<0.001
**MG 12: Antacids incl. Antihistamines**	6916 (68.21)	3224 (31.79)	88833 (90.7)	9113 (9.3)	<0.001
**MG 13: Vitamin D and other Vitamin supplements**	8074 (79.63)	2066 (20.37)	91027 (92.94)	6919 (7.06)	<0.001
**MG 14: Caplacizumab**	10140 (100)	0 (0)	97946 (100)	0 (0)	1
**MG 15: Systemic Hemostatics**	10135 (99.95)	5 (0.05)	97940 (99.99)	6 (0.01)	<0.001
**MG 16: Hereditary angioedema Therapeutics**	10140 (100)	0 (0)	97946 (100)	0 (0)	1
**MG 17: Peripheral Vasodilators**	10040 (99.01)	100 (0.99)	97487 (99.53)	459 (0.47)	<0.001
**MG 18: Hormonal contraceptives and similar hormone preparations**	9812 (96.77)	328 (3.23)	94900 (96.89)	3046 (3.11)	0.5105
**MG 19: Immunoglobulins**	10131 (99.91)	9 (0.09)	97932 (99.99)	14 (0.01)	<0.001
**MG 20: Interferons and CSF**	9894 (97.57)	246 (2.43)	97025 (99.06)	921 (0.94)	<0.001
**MG 21: NSAR and other anti-inflammatory drugs**	6658 (65.66)	3482 (34.34)	79027 (80.68)	18919 (19.32)	<0.001
**MG 22: Gout medications**	9446 (93.16)	694 (6.84)	96224 (98.24)	1722 (1.76)	<0.001
**MG 23: Antiepileptics**	8823 (87.01)	1317 (12.99)	94420 (96.4)	3526 (3.6)	<0.001
**MG 24: Antipsychotics**	6607 (65.16)	3533 (34.84)	85526 (87.32)	12420 (12.68)	<0.001
**MG 25: Rhinological and throat antiseptics**	9430 (93)	710 (7)	94338 (96.32)	3608 (3.68)	<0.001
**MG 26: inhaled anti-obstructive drugs**	7613 (75.08)	2527 (24.92)	90583 (92.48)	7363 (7.52)	<0.001
**MG 27: inhaled steroids**	9589 (94.57)	551 (5.43)	96304 (98.32)	1642 (1.68)	<0.001
**MG 28: other COPD drugs**	9972 (98.34)	168 (1.66)	97451 (99.49)	495 (0.51)	<0.001
**MG 29: Cold and Cough preparations**	8904 (87.81)	1236 (12.19)	94904 (96.89)	3042 (3.11)	<0.001
**MG 30: Systemic Antihistamines**	9519 (93.88)	621 (6.12)	95484 (97.49)	2462 (2.51)	<0.001

Table S7: Medication Groups for COVID-19 hospitalized patients aged ≥75 as compared to controls

Age Group ≥75	COVID (n = 21453)		Control (n = 202638)		
**Medication Groups**	**No: n (%)**	**Yes: n (%)**	**No: n (%)**	**Yes: n (%)**	**p-value**
**MG 1: Anticoagulants**	9466 (44.12)	11987 (55.88)	143675 (70.9)	58963 (29.1)	<0.001
**MG 2: Antibiotics, Antivirals, Antiprotozoals or Anthelmintics**	12165 (56.71)	9288 (43.29)	143838 (70.98)	58800 (29.02)	<0.001
**MG 3: Insulins and other Antidiabetics**	16856 (78.57)	4597 (21.43)	183751 (90.68)	18887 (9.32)	<0.001
**MG 4: "heart" drugs**	17985 (83.83)	3468 (16.17)	181988 (89.81)	20650 (10.19)	<0.001
**MG 5: Antihypertensives incl. Diuretics and Renin-angiotensin-aldosterone system inhibitors**	6983 (32.55)	14470 (67.45)	122661 (60.53)	79977 (39.47)	<0.001
**MG 6: Beta Blockers**	13877 (64.69)	7576 (35.31)	162844 (80.36)	39794 (19.64)	<0.001
**MG 7: Statins, Fibrates incl. Proprotein convertase subtilisin/kexin type 9 inhibitors and Inclisiran**	13255 (61.79)	8198 (38.21)	151745 (74.88)	50893 (25.12)	<0.001
**MG 8: Immunosuppressants and Immunomodulators**	20957 (97.69)	496 (2.31)	200505 (98.95)	2133 (1.05)	<0.001
**MG 9: Systemic Steroids**	18378 (85.67)	3075 (14.33)	184006 (90.81)	18632 (9.19)	<0.001
**MG 10: Chemotherapy**	20577 (95.92)	876 (4.08)	197798 (97.61)	4840 (2.39)	<0.001
**MG 11: Iron supplements, Erythropoietic stimulating agents, Vitamin B12, folic acid**	18772 (87.5)	2681 (12.5)	191822 (94.66)	10816 (5.34)	<0.001
**MG 12: Antacids incl. Antihistamines**	14233 (66.35)	7220 (33.65)	169843 (83.82)	32795 (16.18)	<0.001
**MG 13: Vitamin D and other Vitamin supplements**	16389 (76.39)	5064 (23.61)	175430 (86.57)	27208 (13.43)	<0.001
**MG 14: Caplacizumab**	21453 (100)	0 (0)	202638 (100)	0 (0)	1
**MG 15: Systemic Hemostatics**	21441 (99.94)	12 (0.06)	202604 (99.98)	34 (0.02)	<0.001
**MG 16: Hereditary angioedema Therapeutics**	21453 (100)	0 (0)	202638 (100)	0 (0)	1
**MG 17: Peripheral Vasodilators**	21165 (98.66)	288 (1.34)	200408 (98.9)	2230 (1.1)	0.0016
**MG 18: Hormonal contraceptives and similar hormone preparations**	20955 (97.68)	498 (2.32)	196398 (96.92)	6240 (3.08)	<0.001
**MG 19: Immunoglobulins**	21442 (99.95)	11 (0.05)	202601 (99.98)	37 (0.02)	0.0038
**MG 20: Interferons and CSF**	21215 (98.89)	238 (1.11)	200889 (99.14)	1749 (0.86)	<0.001
**MG 21: NSAR and other anti-inflammatory drugs**	16162 (75.34)	5291 (24.66)	158159 (78.05)	44479 (21.95)	<0.001
**MG 22: Gout medications**	19756 (92.09)	1697 (7.91)	194955 (96.21)	7683 (3.79)	<0.001
**MG 23: Antiepileptics**	18228 (84.97)	3225 (15.03)	188384 (92.97)	14254 (7.03)	<0.001
**MG 24: Antipsychotics**	10254 (47.8)	11199 (52.2)	147484 (72.78)	55154 (27.22)	<0.001
**MG 25: Rhinological and throat antiseptics**	20627 (96.15)	826 (3.85)	195181 (96.32)	7457 (3.68)	0.2156
**MG 26: inhaled anti-obstructive drugs**	17210 (80.22)	4243 (19.78)	183270 (90.44)	19368 (9.56)	<0.001
**MG 27: inhaled steroids**	20787 (96.9)	666 (3.1)	199119 (98.26)	3519 (1.74)	<0.001
**MG 28: other COPD drugs**	21273 (99.16)	180 (0.84)	201758 (99.57)	880 (0.43)	<0.001
**MG 29: Cold and Cough preparations**	19077 (88.92)	2376 (11.08)	191183 (94.35)	11455 (5.65)	<0.001
**MG 30: Systemic Antihistamines**	20062 (93.52)	1391 (6.48)	194682 (96.07)	7956 (3.93)	<0.001

Table S8: Medication Groups for Influenza- hospitalized patients aged 19-40 as compared to controls

Age Group 19-40	Influenza (n = 925)		Control (n = 9283)		
**Medication Groups**	**No: n (%)**	**Yes: n (%)**	**No: n (%)**	**Yes: n (%)**	**p-value**
**MG 1: Anticoagulants**	863 (93.3)	62 (6.7)	9044 (97.43)	239 (2.57)	<0.001
**MG 2: Antibiotics, Antivirals, Antiprotozoals or Anthelmintics**	451 (48.76)	474 (51.24)	7157 (77.1)	2126 (22.9)	<0.001
**MG 3: Insulins and other Antidiabetics**	889 (96.11)	36 (3.89)	9240 (99.54)	43 (0.46)	<0.001
**MG 4: "heart" drugs**	911 (98.49)	14 (1.51)	9234 (99.47)	49 (0.53)	<0.001
**MG 5: Antihypertensives incl. Diuretics and Renin-angiotensin-aldosterone system inhibitors**	862 (93.19)	63 (6.81)	9209 (99.2)	74 (0.8)	<0.001
**MG 6: Beta Blockers**	889 (96.11)	36 (3.89)	9246 (99.6)	37 (0.4)	<0.001
**MG 7: Statins, Fibrates incl. Proprotein convertase subtilisin/kexin type 9 inhibitors and Inclisiran**	899 (97.19)	26 (2.81)	9252 (99.67)	31 (0.33)	<0.001
**MG 8: Immunosuppressants and Immunomodulators**	883 (95.46)	42 (4.54)	9257 (99.72)	26 (0.28)	<0.001
**MG 9: Systemic Steroids**	849 (91.78)	76 (8.22)	9146 (98.52)	137 (1.48)	<0.001
**MG 10: Chemotherapy**	909 (98.27)	16 (1.73)	9274 (99.9)	9 (0.1)	<0.001
**MG 11: Iron supplements, Erythropoietic stimulating agents, Vitamin B12, folic acid**	847 (91.57)	78 (8.43)	9059 (97.59)	224 (2.41)	<0.001
**MG 12: Antacids incl. Antihistamines**	811 (87.68)	114 (12.32)	8992 (96.87)	291 (3.13)	<0.001
**MG 13: Vitamin D and other Vitamin supplements**	852 (92.11)	73 (7.89)	9115 (98.19)	168 (1.81)	<0.001
**MG 14: Caplacizumab**	925 (100)	0 (0)	9283 (100)	0 (0)	1
**MG 15: Systemic Hemostatics**	924 (99.89)	1 (0.11)	9283 (100)	0 (0)	0.0906
**MG 16: Hereditary angioedema Therapeutics**	924 (99.89)	1 (0.11)	9283 (100)	0 (0)	0.0906
**MG 17: Peripheral Vasodilators**	925 (100)	0 (0)	9271 (99.87)	12 (0.13)	0.6173
**MG 18: Hormonal contraceptives and similar hormone preparations**	865 (93.51)	60 (6.49)	9075 (97.76)	208 (2.24)	<0.001
**MG 19: Immunoglobulins**	924 (99.89)	1 (0.11)	9283 (100)	0 (0)	0.0906
**MG 20: Interferons and CSF**	909 (98.27)	16 (1.73)	9219 (99.31)	64 (0.69)	0.0013
**MG 21: NSAR and other anti-inflammatory drugs**	722 (78.05)	203 (21.95)	8260 (88.98)	1023 (11.02)	<0.001
**MG 22: Gout medications**	924 (99.89)	1 (0.11)	9278 (99.95)	5 (0.05)	0.4345
**MG 23: Antiepileptics**	860 (92.97)	65 (7.03)	9200 (99.11)	83 (0.89)	<0.001
**MG 24: Antipsychotics**	783 (84.65)	142 (15.35)	8909 (95.97)	374 (4.03)	<0.001
**MG 25: Rhinological and throat antiseptics**	805 (87.03)	120 (12.97)	8816 (94.97)	467 (5.03)	<0.001
**MG 26: inhaled anti-obstructive drugs**	796 (86.05)	129 (13.95)	9004 (96.99)	279 (3.01)	<0.001
**MG 27: inhaled steroids**	899 (97.19)	26 (2.81)	9194 (99.04)	89 (0.96)	<0.001
**MG 28: other COPD drugs**	905 (97.84)	20 (2.16)	9241 (99.55)	42 (0.45)	<0.001
**MG 29: Cold and Cough preparations**	851 (92)	74 (8)	9134 (98.39)	149 (1.61)	<0.001
**MG 30: Systemic Antihistamines**	869 (93.95)	56 (6.05)	9030 (97.27)	253 (2.73)	<0.001

Table S9: Medication Groups for Influenza- hospitalized patients aged 41 – 64 as compared to controls

Age Group 41-64	Influenza (n = 2342)		Control (n = 23330)		
**Medication Groups**	**No: n (%)**	**Yes: n (%)**	**No: n (%)**	**Yes: n (%)**	**p-value**
**MG 1: Anticoagulants**	1790 (76.43)	552 (23.57)	21857 (93.69)	1473 (6.31)	<0.001
**MG 2: Antibiotics, Antivirals, Antiprotozoals or Anthelmintics**	1058 (45.18)	1284 (54.82)	18163 (77.85)	5167 (22.15)	<0.001
**MG 3: Insulins and other Antidiabetics**	1925 (82.19)	417 (17.81)	22635 (97.02)	695 (2.98)	<0.001
**MG 4: "heart" drugs**	2182 (93.17)	160 (6.83)	22979 (98.5)	351 (1.5)	<0.001
**MG 5: Antihypertensives incl. Diuretics and Renin-angiotensin-aldosterone system inhibitors**	1424 (60.8)	918 (39.2)	20939 (89.75)	2391 (10.25)	<0.001
**MG 6: Beta Blockers**	1824 (77.88)	518 (22.12)	22264 (95.43)	1066 (4.57)	<0.001
**MG 7: Statins, Fibrates incl. Proprotein convertase subtilisin/kexin type 9 inhibitors and Inclisiran**	1727 (73.74)	615 (26.26)	21541 (92.33)	1789 (7.67)	<0.001
**MG 8: Immunosuppressants and Immunomodulators**	2173 (92.78)	169 (7.22)	23174 (99.33)	156 (0.67)	<0.001
**MG 9: Systemic Steroids**	1864 (79.59)	478 (20.41)	22195 (95.14)	1135 (4.86)	<0.001
**MG 10: Chemotherapy**	2290 (97.78)	52 (2.22)	23229 (99.57)	101 (0.43)	<0.001
**MG 11: Iron supplements, Erythropoietic stimulating agents, Vitamin B12, folic acid**	2138 (91.29)	204 (8.71)	22916 (98.23)	414 (1.77)	<0.001
**MG 12: Antacids incl. Antihistamines**	1616 (69)	726 (31)	21589 (92.54)	1741 (7.46)	<0.001
**MG 13: Vitamin D and other Vitamin supplements**	1929 (82.37)	413 (17.63)	22357 (95.83)	973 (4.17)	<0.001
**MG 14: Caplacizumab**	2342 (100)	0 (0)	23330 (100)	0 (0)	1
**MG 15: Systemic Hemostatics**	2340 (99.91)	2 (0.09)	23330 (100)	0 (0)	0.0083
**MG 16: Hereditary angioedema Therapeutics**	2342 (100)	0 (0)	23330 (100)	0 (0)	1
**MG 17: Peripheral Vasodilators**	2322 (99.15)	20 (0.85)	23226 (99.55)	104 (0.45)	0.0105
**MG 18: Hormonal contraceptives and similar hormone preparations**	2248 (95.99)	94 (4.01)	22592 (96.84)	738 (3.16)	0.0312
**MG 19: Immunoglobulins**	2342 (100)	0 (0)	23329 (100)	1 (0)	1
**MG 20: Interferons and CSF**	2260 (96.5)	82 (3.5)	23142 (99.19)	188 (0.81)	<0.001
**MG 21: NSAR and other anti-inflammatory drugs**	1414 (60.38)	928 (39.62)	19033 (81.58)	4297 (18.42)	<0.001
**MG 22: Gout medications**	2262 (96.58)	80 (3.42)	23158 (99.26)	172 (0.74)	<0.001
**MG 23: Antiepileptics**	2025 (86.46)	317 (13.54)	22804 (97.75)	526 (2.25)	<0.001
**MG 24: Antipsychotics**	1555 (66.4)	787 (33.6)	21319 (91.38)	2011 (8.62)	<0.001
**MG 25: Rhinological and throat antiseptics**	2054 (87.7)	288 (12.3)	22276 (95.48)	1054 (4.52)	<0.001
**MG 26: inhaled anti-obstructive drugs**	1654 (70.62)	688 (29.38)	22064 (94.57)	1266 (5.43)	<0.001
**MG 27: inhaled steroids**	2204 (94.11)	138 (5.89)	22939 (98.32)	391 (1.68)	<0.001
**MG 28: other COPD drugs**	2279 (97.31)	63 (2.69)	23193 (99.41)	137 (0.59)	<0.001
**MG 29: Cold and Cough preparations**	1973 (84.24)	369 (15.76)	22758 (97.55)	572 (2.45)	<0.001
**MG 30: Systemic Antihistamines**	2175 (92.87)	167 (7.13)	22727 (97.42)	603 (2.58)	<0.001

Table S10: Medication Groups for Influenza- hospitalized patients aged 65 - 74 as compared to controls

Age Group 65-74	Influenza (n = 2272)		Control (n = 22365)		
Medication Groups	No: n (%)	Yes: n (%)	No: n (%)	Yes: n (%)	p-value
MG 1: Anticoagulants	1275 (56.12)	997 (43.88)	19137 (85.57)	3228 (14.43)	<0.001
MG 2: Antibiotics, Antivirals, Antiprotozoals or Anthelmintics	1120 (49.3)	1152 (50.7)	16675 (74.56)	5690 (25.44)	<0.001
MG 3: Insulins and other Antidiabetics	1695 (74.6)	577 (25.4)	20715 (92.62)	1650 (7.38)	<0.001
MG 4: "heart" drugs	1959 (86.22)	313 (13.78)	21303 (95.25)	1062 (4.75)	<0.001
MG 5: Antihypertensives incl. Diuretics and Renin-angiotensin-aldosterone system inhibitors	871 (38.34)	1401 (61.66)	16888 (75.51)	5477 (24.49)	<0.001
MG 6: Beta Blockers	1464 (64.44)	808 (35.56)	19724 (88.19)	2641 (11.81)	<0.001
MG 7: Statins, Fibrates incl. Proprotein convertase subtilisin/kexin type 9 inhibitors and Inclisiran	1270 (55.9)	1002 (44.1)	18167 (81.23)	4198 (18.77)	<0.001
MG 8: Immunosuppressants and Immunomodulators	2162 (95.16)	110 (4.84)	22140 (98.99)	225 (1.01)	<0.001
MG 9: Systemic Steroids	1770 (77.9)	502 (22.1)	20568 (91.97)	1797 (8.03)	<0.001
MG 10: Chemotherapy	2184 (96.13)	88 (3.87)	22075 (98.7)	290 (1.3)	<0.001
MG 11: Iron supplements, Erythropoietic stimulating agents, Vitamin B12, folic acid	2034 (89.52)	238 (10.48)	21698 (97.02)	667 (2.98)	<0.001
MG 12: Antacids incl. Antihistamines	1415 (62.28)	857 (37.72)	19189 (85.8)	3176 (14.2)	<0.001
MG 13: Vitamin D and other Vitamin supplements	1792 (78.87)	480 (21.13)	20640 (92.29)	1725 (7.71)	<0.001
MG 14: Caplacizumab	2272 (100)	0 (0)	22365 (100)	0 (0)	1
MG 15: Systemic Hemostatics	2272 (100)	0 (0)	22363 (99.99)	2 (0.01)	1
MG 16: Hereditary angioedema Therapeutics	2272 (100)	0 (0)	22365 (100)	0 (0)	1
MG 17: Peripheral Vasodilators	2251 (99.08)	21 (0.92)	22157 (99.07)	208 (0.93)	1
MG 18: Hormonal contraceptives and similar hormone preparations	2195 (96.61)	77 (3.39)	21503 (96.15)	862 (3.85)	0.2957
MG 19: Immunoglobulins	2270 (99.91)	2 (0.09)	22358 (99.97)	7 (0.03)	0.1986
MG 20: Interferons and CSF	2190 (96.39)	82 (3.61)	22083 (98.74)	282 (1.26)	<0.001
MG 21: NSAR and other anti-inflammatory drugs	1449 (63.78)	823 (36.22)	17226 (77.02)	5139 (22.98)	<0.001
MG 22: Gout medications	2110 (92.87)	162 (7.13)	21882 (97.84)	483 (2.16)	<0.001
MG 23: Antiepileptics	1892 (83.27)	380 (16.73)	21541 (96.32)	824 (3.68)	<0.001
MG 24: Antipsychotics	1367 (60.17)	905 (39.83)	19322 (86.39)	3043 (13.61)	<0.001
MG 25: Rhinological and throat antiseptics	2042 (89.88)	230 (10.12)	21219 (94.88)	1146 (5.12)	<0.001
MG 26: inhaled anti-obstructive drugs	1436 (63.2)	836 (36.8)	20377 (91.11)	1988 (8.89)	<0.001
MG 27: inhaled steroids	2131 (93.79)	141 (6.21)	21818 (97.55)	547 (2.45)	<0.001
MG 28: other COPD drugs	2195 (96.61)	77 (3.39)	22227 (99.38)	138 (0.62)	<0.001
MG 29: Cold and Cough preparations	1897 (83.49)	375 (16.51)	21408 (95.72)	957 (4.28)	<0.001
MG 30: Systemic Antihistamines	2086 (91.81)	186 (8.19)	21626 (96.7)	739 (3.3)	1

Table S11: Medication Groups for Influenza- hospitalized aged ≥75 patients as compared to controls

Age Group ≥75	Influenza (n = 5257)		Control (n = 49894)		
**Medication Groups**	**No: n (%)**	**Yes: n (%)**	**No: n (%)**	**Yes: n (%)**	**p-value**
**MG 1: Anticoagulants**	2429 (46.21)	2828 (53.79)	36032 (72.22)	13862 (27.78)	<0.001
**MG 2: Antibiotics, Antivirals, Antiprotozoals or Anthelmintics**	2776 (52.81)	2481 (47.19)	34137 (68.42)	15757 (31.58)	<0.001
**MG 3: Insulins and other Antidiabetics**	4247 (80.79)	1010 (19.21)	45468 (91.13)	4426 (8.87)	<0.001
**MG 4: "heart" drugs**	4343 (82.61)	914 (17.39)	44540 (89.27)	5354 (10.73)	<0.001
**MG 5: Antihypertensives incl. Diuretics and Renin-angiotensin-aldosterone system inhibitors**	1752 (33.33)	3505 (66.67)	31029 (62.19)	18865 (37.81)	<0.001
**MG 6: Beta Blockers**	3440 (65.44)	1817 (34.56)	40244 (80.66)	9650 (19.34)	<0.001
**MG 7: Statins, Fibrates incl. Proprotein convertase subtilisin/kexin type 9 inhibitors and Inclisiran**	3215 (61.16)	2042 (38.84)	38676 (77.52)	11218 (22.48)	<0.001
**MG 8: Immunosuppressants and Immunomodulators**	5160 (98.15)	97 (1.85)	49431 (99.07)	463 (0.93)	<0.001
**MG 9: Systemic Steroids**	4475 (85.12)	782 (14.88)	44799 (89.79)	5095 (10.21)	<0.001
**MG 10: Chemotherapy**	5050 (96.06)	207 (3.94)	48760 (97.73)	1134 (2.27)	<0.001
**MG 11: Iron supplements, Erythropoietic stimulating agents, Vitamin B12, folic acid**	4653 (88.51)	604 (11.49)	46528 (93.25)	3366 (6.75)	<0.001
**MG 12: Antacids incl. Antihistamines**	3581 (68.12)	1676 (31.88)	39089 (78.34)	10805 (21.66)	<0.001
**MG 13: Vitamin D and other Vitamin supplements**	4096 (77.92)	1161 (22.08)	43377 (86.94)	6517 (13.06)	<0.001
**MG 14: Caplacizumab**	5257 (100)	0 (0)	49894 (100)	0 (0)	1
**MG 15: Systemic Hemostatics**	5253 (99.92)	4 (0.08)	49892 (100)	2 (0)	0.0011
**MG 16: Hereditary angioedema Therapeutics**	5257 (100)	0 (0)	49894 (100)	0 (0)	1
**MG 17: Peripheral Vasodilators**	5133 (97.64)	124 (2.36)	49039 (98.29)	855 (1.71)	<0.001
**MG 18: Hormonal contraceptives and similar hormone preparations**	5083 (96.69)	174 (3.31)	48150 (96.5)	1744 (3.5)	0.51
**MG 19: Immunoglobulins**	5254 (99.94)	3 (0.06)	49888 (99.99)	6 (0.01)	0.0469
**MG 20: Interferons and CSF**	5188 (98.69)	69 (1.31)	49382 (98.97)	512 (1.03)	0.0624
**MG 21: NSAR and other anti-inflammatory drugs**	3831 (72.87)	1426 (27.13)	37266 (74.69)	12628 (25.31)	0.0043
**MG 22: Gout medications**	4857 (92.39)	400 (7.61)	47931 (96.07)	1963 (3.93)	<0.001
**MG 23: Antiepileptics**	4486 (85.33)	771 (14.67)	46691 (93.58)	3203 (6.42)	<0.001
**MG 24: Antipsychotics**	2544 (48.39)	2713 (51.61)	36494 (73.14)	13400 (26.86)	<0.001
**MG 25: Rhinological and throat antiseptics**	4935 (93.87)	322 (6.13)	47483 (95.17)	2411 (4.83)	<0.001
**MG 26: inhaled anti-obstructive drugs**	4000 (76.09)	1257 (23.91)	44779 (89.75)	5115 (10.25)	<0.001
**MG 27: inhaled steroids**	5061 (96.27)	196 (3.73)	48804 (97.82)	1090 (2.18)	<0.001
**MG 28: other COPD drugs**	5189 (98.71)	68 (1.29)	49604 (99.42)	290 (0.58)	<0.001
**MG 29: Cold and Cough preparations**	4553 (86.61)	704 (13.39)	46320 (92.84)	3574 (7.16)	<0.001
**MG 30: Systemic Antihistamines**	4924 (93.67)	333 (6.33)	47477 (95.16)	2417 (4.84)	<0.001

Table S12: Medication Groups for Influenza- and COVID-19 hospitalized patients aged 19-40 before and after Propensity Score Matching

	Before PSM				After PSM			
**Age Group 19 - 40**	**Covid (n = 3941)**		**Influenza (n = 925)**		**Covid (n = 1266)**		**Influenza (n = 710)**	
**Medication Groups**	**No: n (%)**	**Yes: n (%)**	**No: n (%)**	**Yes: n (%)**	**No: n (%)**	**Yes: n (%)**	**No: n (%)**	**Yes: n (%)**
**MG 1: Anticoagulants**	3620 (91.85)	321 (8.15)	863 (93.3)	62 (6.7)	1212 (95.73)	54 (4.27)	679 (95.63)	31 (4.37)
**MG 2: Antibiotics, Antivirals, Antiprotozoals or Anthelmintics**	2271 (57.62)	1670 (42.38)	451 (48.76)	474 (51.24)	743 (58.69)	523 (41.31)	394 (55.49)	316 (44.51)
**MG 3: Insulins and other Antidiabetics**	3836 (97.34)	105 (2.66)	889 (96.11)	36 (3.89)	1237 (97.71)	29 (2.29)	697 (98.17)	13 (1.83)
**MG 4: "heart" drugs**	3879 (98.43)	62 (1.57)	911 (98.49)	14 (1.51)	1252 (98.89)	14 (1.11)	705 (99.3)	5 (0.7)
**MG 5: Antihypertensives incl. Diuretics and Renin-angiotensin-aldosterone system inhibitors**	3712 (94.19)	229 (5.81)	862 (93.19)	63 (6.81)	1228 (97)	38 (3)	687 (96.76)	23 (3.24)
**MG 6: Beta Blockers**	3848 (97.64)	93 (2.36)	889 (96.11)	36 (3.89)	1252 (98.89)	14 (1.11)	697 (98.17)	13 (1.83)
**MG 7: Statins, Fibrates incl. Proprotein convertase subtilisin/kexin type 9 inhibitors and Inclisiran**	3845 (97.56)	96 (2.44)	899 (97.19)	26 (2.81)	1252 (98.89)	14 (1.11)	702 (98.87)	8 (1.13)
**MG 8: Immunosuppressants and Immunomodulators**	3837 (97.36)	104 (2.64)	883 (95.46)	42 (4.54)	1257 (99.29)	9 (0.71)	700 (98.59)	10 (1.41)
**MG 9: Systemic Steroids**	3676 (93.28)	265 (6.72)	849 (91.78)	76 (8.22)	1230 (97.16)	36 (2.84)	678 (95.49)	32 (4.51)
**MG 10: Chemotherapy**	3922 (99.52)	19 (0.48)	909 (98.27)	16 (1.73)	1266 (100)	0 (0)	709 (99.86)	1 (0.14)
**MG 11: Iron supplements, Erythropoietic stimulating agents, Vitamin B12, folic acid**	3700 (93.88)	241 (6.12)	847 (91.57)	78 (8.43)	1205 (95.18)	61 (4.82)	676 (95.21)	34 (4.79)
**MG 12: Antacids incl. Antihistamines**	3563 (90.41)	378 (9.59)	811 (87.68)	114 (12.32)	1206 (95.26)	60 (4.74)	659 (92.82)	51 (7.18)
**MG 13: Vitamin D and other Vitamin supplements**	3664 (92.97)	277 (7.03)	852 (92.11)	73 (7.89)	1215 (95.97)	51 (4.03)	683 (96.2)	27 (3.8)
**MG 14: Caplacizumab**	3940 (99.97)	1 (0.03)	925 (100)	0 (0)	1266 (100)	0 (0)	710 (100)	0 (0)
**MG 15: Systemic Hemostatics**	3940 (99.97)	1 (0.03)	924 (99.89)	1 (0.11)	1266 (100)	0 (0)	709 (99.86)	1 (0.14)
**MG 16: Hereditary angioedema Therapeutics**	3940 (99.97)	1 (0.03)	924 (99.89)	1 (0.11)	1266 (100)	0 (0)	709 (99.86)	1 (0.14)
**MG 17: Peripheral Vasodilators**	3936 (99.87)	5 (0.13)	925 (100)	0 (0)	1265 (99.92)	1 (0.08)	710 (100)	0 (0)
**MG 18: Hormonal contraceptives and similar hormone preparations**	3752 (95.2)	189 (4.8)	865 (93.51)	60 (6.49)	1203 (95.02)	63 (4.98)	672 (94.65)	38 (5.35)
**MG 19: Immunoglobulins**	3937 (99.9)	4 (0.1)	924 (99.89)	1 (0.11)	1266 (100)	0 (0)	710 (100)	0 (0)
**MG 20: Interferons and CSF**	3893 (98.78)	48 (1.22)	909 (98.27)	16 (1.73)	1259 (99.45)	7 (0.55)	701 (98.73)	9 (1.27)
**MG 21: NSAR and other anti-inflammatory drugs**	3010 (76.38)	931 (23.62)	722 (78.05)	203 (21.95)	1065 (84.12)	201 (15.88)	577 (81.27)	133 (18.73)
**MG 22: Gout medications**	3922 (99.52)	19 (0.48)	924 (99.89)	1 (0.11)	1266 (100)	0 (0)	710 (100)	0 (0)
**MG 23: Antiepileptics**	3770 (95.66)	171 (4.34)	860 (92.97)	65 (7.03)	1233 (97.39)	33 (2.61)	688 (96.9)	22 (3.1)
**MG 24: Antipsychotics**	3446 (87.44)	495 (12.56)	783 (84.65)	142 (15.35)	1171 (92.5)	95 (7.5)	649 (91.41)	61 (8.59)
**MG 25: Rhinological and throat antiseptics**	3570 (90.59)	371 (9.41)	805 (87.03)	120 (12.97)	1179 (93.13)	87 (6.87)	645 (90.85)	65 (9.15)
**MG 26: inhaled anti-obstructive drugs**	3438 (87.24)	503 (12.76)	796 (86.05)	129 (13.95)	1161 (91.71)	105 (8.29)	643 (90.56)	67 (9.44)
**MG 27: inhaled steroids**	3746 (95.05)	195 (4.95)	899 (97.19)	26 (2.81)	1248 (98.58)	18 (1.42)	698 (98.31)	12 (1.69)
**MG 28: other COPD drugs**	3904 (99.06)	37 (0.94)	905 (97.84)	20 (2.16)	1262 (99.68)	4 (0.32)	704 (99.15)	6 (0.85)
**MG 29: Cold and Cough preparations**	3727 (94.57)	214 (5.43)	851 (92)	74 (8)	1235 (97.55)	31 (2.45)	674 (94.93)	36 (5.07)
**MG 30: Systemic Antihistamines**	3762 (95.46)	179 (4.54)	869 (93.95)	56 (6.05)	1239 (97.87)	27 (2.13)	687 (96.76)	23 (3.24)

Table S13: Medication Groups for Influenza- and COVID-19 hospitalized patients aged 41 – 64 before and after Propensity Score Matching

	Before PSM				After PSM			
**Age Group 41 - 64**	**Covid (n = 16481)**		**Influenza (n = 3242)**		**Covid (n = 3978)**		**Covid (n = 2124)**	
**Medication Groups**	**No: n (%)**	**Yes: n (%)**	**No: n (%)**	**Yes: n (%)**	**No: n (%)**	**Yes: n (%)**	**No: n (%)**	**Yes: n (%)**
**MG 1: Anticoagulants**	13288 (80.63)	3193 (19.37)	1790 (76.43)	552 (23.57)	3242 (81.5)	736 (18.5)	1672 (78.72)	452 (21.28)
**MG 2: Antibiotics, Antivirals, Antiprotozoals or Anthelmintics**	9464 (57.42)	7017 (42.58)	1058 (45.18)	1284 (54.82)	1951 (49.04)	2027 (50.96)	1030 (48.49)	1094 (51.51)
**MG 3: Insulins and other Antidiabetics**	14320 (86.89)	2161 (13.11)	1925 (82.19)	417 (17.81)	3403 (85.55)	575 (14.45)	1790 (84.27)	334 (15.73)
**MG 4: "heart" drugs**	15821 (96)	660 (4)	2182 (93.17)	160 (6.83)	3781 (95.05)	197 (4.95)	2008 (94.54)	116 (5.46)
**MG 5: Antihypertensives incl. Diuretics and Renin-angiotensin-aldosterone system inhibitors**	11493 (69.73)	4988 (30.27)	1424 (60.8)	918 (39.2)	2647 (66.54)	1331 (33.46)	1366 (64.31)	758 (35.69)
**MG 6: Beta Blockers**	14397 (87.36)	2084 (12.64)	1824 (77.88)	518 (22.12)	3344 (84.06)	634 (15.94)	1741 (81.97)	383 (18.03)
**MG 7: Statins, Fibrates incl. Proprotein convertase subtilisin/kexin type 9 inhibitors and Inclisiran**	13112 (79.56)	3369 (20.44)	1727 (73.74)	615 (26.26)	3101 (77.95)	877 (22.05)	1606 (75.61)	518 (24.39)
**MG 8: Immunosuppressants and Immunomodulators**	15829 (96.04)	652 (3.96)	2173 (92.78)	169 (7.22)	3806 (95.68)	172 (4.32)	2008 (94.54)	116 (5.46)
**MG 9: Systemic Steroids**	14478 (87.85)	2003 (12.15)	1864 (79.59)	478 (20.41)	3428 (86.17)	550 (13.83)	1768 (83.24)	356 (16.76)
**MG 10: Chemotherapy**	16235 (98.51)	246 (1.49)	2290 (97.78)	52 (2.22)	3919 (98.52)	59 (1.48)	2078 (97.83)	46 (2.17)
**MG 11: Iron supplements, Erythropoietic stimulating agents, Vitamin B12, folic acid**	15671 (95.09)	810 (4.91)	2138 (91.29)	204 (8.71)	3769 (94.75)	209 (5.25)	1976 (93.03)	148 (6.97)
**MG 12: Antacids incl. Antihistamines**	13462 (81.68)	3019 (18.32)	1616 (69)	726 (31)	3044 (76.52)	934 (23.48)	1571 (73.96)	553 (26.04)
**MG 13: Vitamin D and other Vitamin supplements**	14604 (88.61)	1877 (11.39)	1929 (82.37)	413 (17.63)	3454 (86.83)	524 (13.17)	1808 (85.12)	316 (14.88)
**MG 14: Caplacizumab**	16481 (100)	0 (0)	2342 (100)	0 (0)	3978 (100)	0 (0)	2124 (100)	0 (0)
**MG 15: Systemic Hemostatics**	16476 (99.97)	5 (0.03)	2340 (99.91)	2 (0.09)	3975 (99.92)	3 (0.08)	2122 (99.91)	2 (0.09)
**MG 16: Hereditary angioedema Therapeutics**	16481 (100)	0 (0)	2342 (100)	0 (0)	3978 (100)	0 (0)	2124 (100)	0 (0)
**MG 17: Peripheral Vasodilators**	16412 (99.58)	69 (0.42)	2322 (99.15)	20 (0.85)	3952 (99.35)	26 (0.65)	2107 (99.2)	17 (0.8)
**MG 18: Hormonal contraceptives and similar hormone preparations**	15922 (96.61)	559 (3.39)	2248 (95.99)	94 (4.01)	3834 (96.38)	144 (3.62)	2044 (96.23)	80 (3.77)
**MG 19: Immunoglobulins**	16471 (99.94)	10 (0.06)	2342 (100)	0 (0)	3977 (99.97)	1 (0.03)	2124 (100)	0 (0)
**MG 20: Interferons and CSF**	16199 (98.29)	282 (1.71)	2260 (96.5)	82 (3.5)	3893 (97.86)	85 (2.14)	2063 (97.13)	61 (2.87)
**MG 21: NSAR and other anti-inflammatory drugs**	10852 (65.85)	5629 (34.15)	1414 (60.38)	928 (39.62)	2565 (64.48)	1413 (35.52)	1318 (62.05)	806 (37.95)
**MG 22: Gout medications**	16096 (97.66)	385 (2.34)	2262 (96.58)	80 (3.42)	3888 (97.74)	90 (2.26)	2061 (97.03)	63 (2.97)
**MG 23: Antiepileptics**	15174 (92.07)	1307 (7.93)	2025 (86.46)	317 (13.54)	3617 (90.93)	361 (9.07)	1891 (89.03)	233 (10.97)
**MG 24: Antipsychotics**	12866 (78.07)	3615 (21.93)	1555 (66.4)	787 (33.6)	2939 (73.88)	1039 (26.12)	1510 (71.09)	614 (28.91)
**MG 25: Rhinological and throat antiseptics**	15105 (91.65)	1376 (8.35)	2054 (87.7)	288 (12.3)	3605 (90.62)	373 (9.38)	1893 (89.12)	231 (10.88)
**MG 26: inhaled anti-obstructive drugs**	13354 (81.03)	3127 (18.97)	1654 (70.62)	688 (29.38)	3040 (76.42)	938 (23.58)	1603 (75.47)	521 (24.53)
**MG 27: inhaled steroids**	3746 (95.05)	195 (4.95)	2204 (94.11)	138 (5.89)	3797 (95.45)	181 (4.55)	2003 (94.3)	121 (5.7)
**MG 28: other COPD drugs**	3904 (99.06)	37 (0.94)	2279 (97.31)	63 (2.69)	3898 (97.99)	80 (2.01)	2077 (97.79)	47 (2.21)
**MG 29: Cold and Cough preparations**	3727 (94.57)	214 (5.43)	1973 (84.24)	369 (15.76)	3621 (91.03)	357 (8.97)	1851 (87.15)	273 (12.85)
**MG 30: Systemic Antihistamines**	3762 (95.46)	179 (4.54)	2175 (92.87)	167 (7.13)	3744 (94.12)	234 (5.88)	1983 (93.36)	141 (6.64)

Table S14: Medication Groups for Influenza- and COVID-19 hospitalized patients aged 65-74 before and after Propensity Score Matching

	Before PSM				After PSM			
**Age Group 65-74**	**Covid (n = 10140)**		**Influenza (n = 2272)**		**Covid (n = 3666)**		**Influenza (n = 2015)**	
**Medication Groups**	**No: n (%)**	**Yes: n (%)**	**No: n (%)**	**Yes: n (%)**	**No: n (%)**	**Yes: n (%)**	**No: n (%)**	**Yes: n (%)**
**MG 1: Anticoagulants**	6027 (59.44)	4113 (40.56)	1275 (56.12)	997 (43.88)	2229 (60.8)	1437 (39.2)	1169 (58.01)	846 (41.99)
**MG 2: Antibiotics, Antivirals, Antiprotozoals or Anthelmintics**	5743 (56.64)	4397 (43.36)	1120 (49.3)	1152 (50.7)	1989 (54.26)	1677 (45.74)	1062 (52.7)	953 (47.3)
**MG 3: Insulins and other Antidiabetics**	7455 (73.52)	2685 (26.48)	1695 (74.6)	577 (25.4)	2790 (76.1)	876 (23.9)	1499 (74.39)	516 (25.61)
**MG 4: "heart" drugs**	9119 (89.93)	1021 (10.07)	1959 (86.22)	313 (13.78)	3304 (90.13)	362 (9.87)	1794 (89.03)	221 (10.97)
**MG 5: Antihypertensives incl. Diuretics and Renin-angiotensin-aldosterone system inhibitors**	4197 (41.39)	5943 (58.61)	871 (38.34)	1401 (61.66)	1597 (43.56)	2069 (56.44)	810 (40.2)	1205 (59.8)
**MG 6: Beta Blockers**	7046 (69.49)	3094 (30.51)	1464 (64.44)	808 (35.56)	2550 (69.56)	1116 (30.44)	1357 (67.34)	658 (32.66)
**MG 7: Statins, Fibrates incl. Proprotein convertase subtilisin/kexin type 9 inhibitors and Inclisiran**	5897 (58.16)	4243 (41.84)	1270 (55.9)	1002 (44.1)	2188 (59.68)	1478 (40.32)	1163 (57.72)	852 (42.28)
**MG 8: Immunosuppressants and Immunomodulators**	9655 (95.22)	485 (4.78)	2162 (95.16)	110 (4.84)	3500 (95.47)	166 (4.53)	1918 (95.19)	97 (4.81)
**MG 9: Systemic Steroids**	8410 (82.94)	1730 (17.06)	1770 (77.9)	502 (22.1)	3051 (83.22)	615 (16.78)	1647 (81.74)	368 (18.26)
**MG 10: Chemotherapy**	9770 (96.35)	370 (3.65)	2184 (96.13)	88 (3.87)	3529 (96.26)	137 (3.74)	1942 (96.38)	73 (3.62)
**MG 11: Iron supplements, Erythropoietic stimulating agents, Vitamin B12, folic acid**	9255 (91.27)	885 (8.73)	2034 (89.52)	238 (10.48)	3379 (92.17)	287 (7.83)	1832 (90.92)	183 (9.08)
**MG 12: Antacids incl. Antihistamines**	6916 (68.21)	3224 (31.79)	1415 (62.28)	857 (37.72)	2504 (68.3)	1162 (31.7)	1338 (66.4)	677 (33.6)
**MG 13: Vitamin D and other Vitamin supplements**	8074 (79.63)	2066 (20.37)	1792 (78.87)	480 (21.13)	2988 (81.51)	678 (18.49)	1610 (79.9)	405 (20.1)
**MG 14: Caplacizumab**	10140 (100)	0 (0)	2272 (100)	0 (0)	3666 (100)	0 (0)	2015 (100)	0 (0)
**MG 15: Systemic Hemostatics**	10135 (99.95)	5 (0.05)	2272 (100)	0 (0)	3664 (99.95)	2 (0.05)	2015 (100)	0 (0)
**MG 16: Hereditary angioedema Therapeutics**	10140 (100)	0 (0)	2272 (100)	0 (0)	3666 (100)	0 (0)	2015 (100)	0 (0)
**MG 17: Peripheral Vasodilators**	10040 (99.01)	100 (0.99)	2251 (99.08)	21 (0.92)	3624 (98.85)	42 (1.15)	1998 (99.16)	17 (0.84)
**MG 18: Hormonal contraceptives and similar hormone preparations**	9812 (96.77)	328 (3.23)	2195 (96.61)	77 (3.39)	3558 (97.05)	108 (2.95)	1946 (96.58)	69 (3.42)
**MG 19: Immunoglobulins**	10131 (99.91)	9 (0.09)	2270 (99.91)	2 (0.09)	3663 (99.92)	3 (0.08)	2013 (99.9)	2 (0.1)
**MG 20: Interferons and CSF**	9894 (97.57)	246 (2.43)	2190 (96.39)	82 (3.61)	3584 (97.76)	82 (2.24)	1956 (97.07)	59 (2.93)
**MG 21: NSAR and other anti-inflammatory drugs**	6658 (65.66)	3482 (34.34)	1449 (63.78)	823 (36.22)	2486 (67.81)	1180 (32.19)	1326 (65.81)	689 (34.19)
**MG 22: Gout medications**	9446 (93.16)	694 (6.84)	2110 (92.87)	162 (7.13)	3428 (93.51)	238 (6.49)	1875 (93.05)	140 (6.95)
**MG 23: Antiepileptics**	8823 (87.01)	1317 (12.99)	1892 (83.27)	380 (16.73)	3173 (86.55)	493 (13.45)	1706 (84.67)	309 (15.33)
**MG 24: Antipsychotics**	6607 (65.16)	3533 (34.84)	1367 (60.17)	905 (39.83)	2370 (64.65)	1296 (35.35)	1274 (63.23)	741 (36.77)
**MG 25: Rhinological and throat antiseptics**	9430 (93)	710 (7)	2042 (89.88)	230 (10.12)	3415 (93.15)	251 (6.85)	1832 (90.92)	183 (9.08)
**MG 26: inhaled anti-obstructive drugs**	7613 (75.08)	2527 (24.92)	1436 (63.2)	836 (36.8)	2685 (73.24)	981 (26.76)	1404 (69.68)	611 (30.32)
**MG 27: inhaled steroids**	9589 (94.57)	551 (5.43)	2131 (93.79)	141 (6.21)	3465 (94.52)	201 (5.48)	1905 (94.54)	110 (5.46)
**MG 28: other COPD drugs**	9972 (98.34)	168 (1.66)	2195 (96.61)	77 (3.39)	3616 (98.64)	50 (1.36)	1987 (98.61)	28 (1.39)
**MG 29: Cold and Cough preparations**	8904 (87.81)	1236 (12.19)	1897 (83.49)	375 (16.51)	3230 (88.11)	436 (11.89)	1735 (86.1)	280 (13.9)
**MG 30: Systemic Antihistamines**	9519 (93.88)	621 (6.12)	2086 (91.81)	186 (8.19)	3440 (93.84)	226 (6.16)	1893 (93.95)	122 (6.05)

Table S15: Medication Groups for Influenza- and COVID-19 hospitalized patients aged ≥75 before and after Propensity Score Matching

	Before PSM				After PSM			
**Age Group ≥75**	**Covid (n = 21453)**		**Influenza (n = 5257)**		**Covid (n = 9579)**		**Influenza (n = 5005)**	
**Medication Groups**	**No: n (%)**	**Yes: n (%)**	**No: n (%)**	**Yes: n (%)**	**No: n (%)**	**Yes: n (%)**	**No: n (%)**	**Yes: n (%)**
**MG 1: Anticoagulants**	9466 (44.12)	11987 (55.88)	2429 (46.21)	2828 (53.79)	4425 (46.19)	5154 (53.81)	2303 (46.01)	2702 (53.99)
**MG 2: Antibiotics, Antivirals, Antiprotozoals or Anthelmintics**	12165 (56.71)	9288 (43.29)	2776 (52.81)	2481 (47.19)	5321 (55.55)	4258 (44.45)	2709 (54.13)	2296 (45.87)
**MG 3: Insulins and other Antidiabetics**	16856 (78.57)	4597 (21.43)	4247 (80.79)	1010 (19.21)	7714 (80.53)	1865 (19.47)	4032 (80.56)	973 (19.44)
**MG 4: "heart" drugs**	17985 (83.83)	3468 (16.17)	4343 (82.61)	914 (17.39)	8040 (83.93)	1539 (16.07)	4166 (83.24)	839 (16.76)
**MG 5: Antihypertensives incl. Diuretics and Renin-angiotensin-aldosterone system inhibitors**	6983 (32.55)	14470 (67.45)	1752 (33.33)	3505 (66.67)	3282 (34.26)	6297 (65.74)	1681 (33.59)	3324 (66.41)
**MG 6: Beta Blockers**	13877 (64.69)	7576 (35.31)	3440 (65.44)	1817 (34.56)	6383 (66.64)	3196 (33.36)	3279 (65.51)	1726 (34.49)
**MG 7: Statins, Fibrates incl. Proprotein convertase subtilisin/kexin type 9 inhibitors and Inclisiran**	13255 (61.79)	8198 (38.21)	3215 (61.16)	2042 (38.84)	5993 (62.56)	3586 (37.44)	3093 (61.8)	1912 (38.2)
**MG 8: Immunosuppressants and Immunomodulators**	20957 (97.69)	496 (2.31)	5160 (98.15)	97 (1.85)	9413 (98.27)	166 (1.73)	4913 (98.16)	92 (1.84)
**MG 9: Systemic Steroids**	18378 (85.67)	3075 (14.33)	4475 (85.12)	782 (14.88)	8287 (86.51)	1292 (13.49)	4286 (85.63)	719 (14.37)
**MG 10: Chemotherapy**	20577 (95.92)	876 (4.08)	5050 (96.06)	207 (3.94)	9201 (96.05)	378 (3.95)	4806 (96.02)	199 (3.98)
**MG 11: Iron supplements, Erythropoietic stimulating agents, Vitamin B12, folic acid**	18772 (87.5)	2681 (12.5)	4653 (88.51)	604 (11.49)	8500 (88.74)	1079 (11.26)	4440 (88.71)	565 (11.29)
**MG 12: Antacids incl. Antihistamines**	14233 (66.35)	7220 (33.65)	3581 (68.12)	1676 (31.88)	6567 (68.56)	3012 (31.44)	3409 (68.11)	1596 (31.89)
**MG 13: Vitamin D and other Vitamin supplements**	16389 (76.39)	5064 (23.61)	4096 (77.92)	1161 (22.08)	7493 (78.22)	2086 (21.78)	3900 (77.92)	1105 (22.08)
**MG 14: Caplacizumab**	21453 (100)	0 (0)	5257 (100)	0 (0)	9579 (100)	0 (0)	5005 (100)	0 (0)
**MG 15: Systemic Hemostatics**	21441 (99.94)	12 (0.06)	5253 (99.92)	4 (0.08)	9576 (99.97)	3 (0.03)	5001 (99.92)	4 (0.08)
**MG 16: Hereditary angioedema Therapeutics**	21453 (100)	0 (0)	5257 (100)	0 (0)	9579 (100)	0 (0)	5005 (100)	0 (0)
**MG 17: Peripheral Vasodilators**	21165 (98.66)	288 (1.34)	5133 (97.64)	124 (2.36)	9511 (99.29)	68 (0.71)	4962 (99.14)	43 (0.86)
**MG 18: Hormonal contraceptives and similar hormone preparations**	20955 (97.68)	498 (2.32)	5083 (96.69)	174 (3.31)	9361 (97.72)	218 (2.28)	4844 (96.78)	161 (3.22)
**MG 19: Immunoglobulins**	21442 (99.95)	11 (0.05)	5254 (99.94)	3 (0.06)	9577 (99.98)	2 (0.02)	5002 (99.94)	3 (0.06)
**MG 20: Interferons and CSF**	21215 (98.89)	238 (1.11)	5188 (98.69)	69 (1.31)	9481 (98.98)	98 (1.02)	4950 (98.9)	55 (1.1)
**MG 21: NSAR and other anti-inflammatory drugs**	16162 (75.34)	5291 (24.66)	3831 (72.87)	1426 (27.13)	7185 (75.01)	2394 (24.99)	3711 (74.15)	1294 (25.85)
**MG 22: Gout medications**	19756 (92.09)	1697 (7.91)	4857 (92.39)	400 (7.61)	8878 (92.68)	701 (7.32)	4630 (92.51)	375 (7.49)
**MG 23: Antiepileptics**	18228 (84.97)	3225 (15.03)	4486 (85.33)	771 (14.67)	8225 (85.86)	1354 (14.14)	4275 (85.41)	730 (14.59)
**MG 24: Antipsychotics**	10254 (47.8)	11199 (52.2)	2544 (48.39)	2713 (51.61)	4657 (48.62)	4922 (51.38)	2433 (48.61)	2572 (51.39)
**MG 25: Rhinological and throat antiseptics**	20627 (96.15)	826 (3.85)	4935 (93.87)	322 (6.13)	9230 (96.36)	349 (3.64)	4715 (94.21)	290 (5.79)
**MG 26: inhaled anti-obstructive drugs**	17210 (80.22)	4243 (19.78)	4000 (76.09)	1257 (23.91)	7621 (79.56)	1958 (20.44)	3917 (78.26)	1088 (21.74)
**MG 27: inhaled steroids**	20787 (96.9)	666 (3.1)	5061 (96.27)	196 (3.73)	9276 (96.84)	303 (3.16)	4843 (96.76)	162 (3.24)
**MG 28: other COPD drugs**	21273 (99.16)	180 (0.84)	5189 (98.71)	68 (1.29)	9519 (99.37)	60 (0.63)	4967 (99.24)	38 (0.76)
**MG 29: Cold and Cough preparations**	19077 (88.92)	2376 (11.08)	4553 (86.61)	704 (13.39)	8551 (89.27)	1028 (10.73)	4344 (86.79)	661 (13.21)
**MG 30: Systemic Antihistamines**	20062 (93.52)	1391 (6.48)	4924 (93.67)	333 (6.33)	8993 (93.88)	586 (6.12)	4696 (93.83)	309 (6.17)

Analysis of the COVID and Influenza patient cohorts

Tables S16-S19 show the results of the simple and multivariable Cox regression models comparing **all-cause mortality** of propensity score matched COVID-19 and Influenza patients including group, sex, age, and polypharmacy with clustering variable region (federal state) and corresponding medication groups as confounding factors.

Tables S20-S23 show the results of the simple and multivariable Cox regression models for **all-cause mortality within COVID-19 patients** including group, sex, age, and polypharmacy with clustering variable region (federal state) and corresponding medication groups as confounding factors.

Tables S24-S27 show the results of the simple and multivariable Cox regression models for **all-cause mortality within Influenza patients** including group, sex, age, and polypharmacy with clustering variable region (federal state) and corresponding medication groups as confounding factors.

Tables S28-S31 show the results of the simple and multivariable Cox regression models comparing **in-hospital mortality** of propensity score matched COVID-19 and Influenza patients including group, sex, age, and polypharmacy with clustering variable region (federal state) and corresponding medication groups as confounding factors.

Tables S32-S35 show the results of the simple and multivariable Cox regression models comparing **all-cause mortality conditioned on hospital survival** of propensity score matched COVID-19 and Influenza patients including group, sex, age, and polypharmacy with clustering variable region (federal state) and corresponding medication groups as confounding factors.

Tables S36-S39 show the results of the simple and multivariable competing risk regression models comparing **readmission** of propensity score matched COVID-19 and Influenza patients including group, sex, age, and polypharmacy with clustering variable region (federal state) and corresponding medication groups as confounding factors and competing risk death.

Tables S40-S43 show the results of the simple and multivariable competing risk regression models comparing **hospital discharge** of propensity score matched COVID-19 and Influenza patients including group, sex, age, and polypharmacy with clustering variable region (federal state) and corresponding medication groups as confounding factors and competing risk death.

All-Cause Mortality

Table S16: Results of the simple and multivariable Cox regression for all-cause mortality comparing propensity score matched COVID-19 and Influenza patients of age 19 to 40.

	Simple		Multivariable	
**Confounder**	**HR (CI)**	**p- value**	**HR (CI)**	**p- value**
**Group: Flu (ref: Covid)**	1.21 (0.68-2.20)	0.5091	1.20 (0.67-2.14)	0.5453
**Age Group: 31-35 (ref: 36-40)**	1.02 (0.30-3.55)	0.9691	1.13 (0.39-3.29)	0.8181
**Age Group: 26-30 (ref: 36-40)**	1.06 (0.34-3.26)	0.9233	1.42 (0.48-4.20)	0.5259
**Age Group: 19-25 (ref: 36-40)**	1.92 (0.49-7.5)	0.3499	2.17 (0.74-6.35)	0.1568
**Sex: W (ref: M)**	0.30 (0.10-0.90)	0.0324	0.24 (0.08-0.7)	0.0093
**Number Medication groups: 2-5 (ref: 0-1)**	3.56 (2.16-5.86)	<0.001	1.58 (0.62-4.02)	0.3349
**Number Medication groups: >=6 (ref: 0-1)**	13.64 (10.59-17.58)	<0.001	0.90 (0.17-4.70)	0.8999
**Anticoagulants: Yes (ref: No)**	3.57 (1.73-7.37)	<0.001	3.98 (1.71-9.25)	0.0013
**Antibiotics, Antivirals, Antiprotozoals or Anthelmintics: Yes (ref: No)**	0.99 (0.66-1.49)	0.9625		
**Insulins and other Antidiabetics: Yes (ref: No)**	5.06 (0.72-35.49)	0.1031		
**"heart" drugs: Yes (ref: No)**	5.76 (3.21-10.31)	<0.001	6.75 (2.22-20.57)	<0.001
**Antihypertensives incl. Diuretics and Renin-angiotensin-aldosterone system inhibitors: Yes (ref: No)**	2.34 (0.57-9.66)	0.2400		
**Beta Blockers: Yes (ref: No)**	0.82 (0.36-1.84)	0.6231		
**Statins, Fibrates incl. Proprotein convertase subtilisin/kexin type 9 inhibitors and Inclisiran: Yes (ref: No)**	1.35 (0.25-7.47)	0.7278		
**Immunosuppressants and Immunomodulators: Yes (ref: No)**	1.11 (0.55-2.24)	0.7682		
**Systemic Steroids: Yes (ref: No)**	1.49 (0.56-3.93)	0.4210		
**Chemotherapy: Yes (ref: No)**	NA (NA-NA)	NA		
**Iron supplements, Erythropoietic stimulating agents, Vitamin B12, folic acid: Yes (ref: No)**	1.52 (0.85-2.71)	0.1610		
**Antacids incl. Antihistamines: Yes (ref: No)**	3.30 (1.34-8.12)	0.0094	3.97 (1.63-9.68)	0.0024
**Vitamin D and other Vitamin supplements: Yes (ref: No)**	0.62 (0.21-1.84)	0.3886		
**Caplacizumab: Yes (ref: No)**	NA (NA-NA)	NA		
**Systemic Hemostatics: Yes (ref: No)**	NA (NA-NA)	NA		
**Hereditary angioedema Therapeutics: Yes (ref: No)**	NA (NA-NA)	NA		
**Peripheral Vasodilators: Yes (ref: No)**	NA (NA-NA)	NA		
**Immunoglobulins: Yes (ref: No)**	NA (NA-NA)	NA		
**Interferons and CSF: Yes (ref: No)**	NA (NA-NA)	NA		
**NSAR and other anti-inflammatory drugs: Yes (ref: No)**	0.44 (0.14-1.36)	0.1554		
**Gout medications: Yes (ref: No)**	NA (NA-NA)	NA		
**Antiepileptics: Yes (ref: No)**	3.21 (0.94-10.98)	0.0624	3.36 (1.20-9.36)	0.0206
**Antipsychotics: Yes (ref: No)**	1.06 (0.37-3.05)	0.9132		
**inhaled anti-obstructive drugs: Yes (ref: No)**	0.74 (0.26-2.11)	0.5762		
**inhaled steroids: Yes (ref: No)**	2.36 (1.36-4.08)	0.0022	4.03 (1.83-8.86)	<0.001
**other COPD drugs: Yes (ref: No)**	NA (NA-NA)	NA		
**Systemic Antihistamines: Yes (ref: No)**	0.73 (0.38-1.4)	0.3437		

Table S17: Results of the simple and multivariable Cox regression models for all-cause mortality comparing propensity score matched COVID-19 and Influenza patients of age 41 to 64.

	Simple		Multivariable	
**Confounder**	**HR (CI)**	**p- value**	**HR (CI)**	**p- value**
**Group: Flu (ref: Covid)**	0.79 (0.66-0.96)	0.0157	0.79 (0.66-0.94)	0.0094
**Age Group: 56-60 (ref: 61-64)**	0.75 (0.61-0.92)	0.0066	0.75 (0.59-0.94)	0.0137
**Age Group: 51-55 (ref: 61-64)**	0.48 (0.40-0.59)	<0.001	0.46 (0.37-0.58)	<0.001
**Age Group: 46-50 (ref: 61-64)**	0.39 (0.32-0.46)	<0.001	0.36 (0.31-0.42)	<0.001
**Age Group: 41-55 (ref: 61-64)**	0.30 (0.19-0.45)	<0.001	0.28 (0.19-0.44)	<0.001
**Sex: W (ref: M)**	0.56 (0.48-0.65)	<0.001	0.52 (0.45-0.61)	<0.001
**Number Medication groups: 2-5 (ref: 0-1)**	2.04 (1.70-2.45)	<0.001	1.83 (1.47-2.28)	<0.001
**Number Medication groups: 6-10 (ref: 0-1)**	4.41 (3.76-5.18)	<0.001	2.66 (1.92-3.69)	<0.001
**Number Medication groups: >=11 (ref: 0-1)**	5.78 (4.58-7.29)	<0.001	2.13 (1.46-3.11)	<0.001
**Anticoagulants: Yes (ref: No)**	1.28 (1.10-1.50)	0.0018	1.39 (1.12-1.72)	0.0029
**Antibiotics, Antivirals, Antiprotozoals or Anthelmintics: Yes (ref: No)**	0.93 (0.83-1.04)	0.1931		
**Insulins and other Antidiabetics: Yes (ref: No)**	1.05 (0.88-1.26)	0.5628		
**"heart" drugs: Yes (ref: No)**	0.96 (0.84-1.09)	0.4904		
**Antihypertensives incl. Diuretics and Renin-angiotensin-aldosterone system inhibitors: Yes (ref: No)**	1.07 (0.92-1.25)	0.3532		
**Beta Blockers: Yes (ref: No)**	0.96 (0.80-1.14)	0.6207		
**Statins, Fibrates incl. Proprotein convertase subtilisin/kexin type 9 inhibitors and Inclisiran: Yes (ref: No)**	0.61 (0.49-0.77)	<0.001	0.75 (0.57-0.98)	0.0339
**Immunosuppressants and Immunomodulators: Yes (ref: No)**	1.02 (0.71-1.47)	0.9187		
**Systemic Steroids: Yes (ref: No)**	1.21 (1.00-1.46)	0.0464	1.25 (1.04-1.51)	0.0173
**Chemotherapy: Yes (ref: No)**	2.08 (1.50-2.88)	<0.001	1.72 (1.18-2.51)	0.0052
**Iron supplements, Erythropoietic stimulating agents, Vitamin B12, folic acid: Yes (ref: No)**	2.27 (1.80-2.86)	<0.001	1.77 (1.43-2.2)	<0.001
**Antacids incl. Antihistamines: Yes (ref: No)**	1.06 (0.99-1.13)	0.1052		
**Vitamin D and other Vitamin supplements: Yes (ref: No)**	1.69 (1.33-2.15)	<0.001	1.46 (1.2-1.79)	<0.001
**Caplacizumab: Yes (ref: No)**	NA (NA-NA)	NA		
**Systemic Hemostatics: Yes (ref: No)**	2.63 (1.31-5.28)	0.0065	2.01 (1.29-3.15)	0.0021
**Hereditary angioedema Therapeutics: Yes (ref: No)**	NA (NA-NA)	NA		
**Peripheral Vasodilators: Yes (ref: No)**	0.62 (0.25-1.59)	0.3222		
**Immunoglobulins: Yes (ref: No)**	NA (NA-NA)	NA		
**Interferons and CSF: Yes (ref: No)**	1.9 (1.48-2.44)	<0.001	1.53 (1.06-2.22)	0.0232
**NSAR and other anti-inflammatory drugs: Yes (ref: No)**	0.64 (0.58-0.71)	<0.001	0.69 (0.61-0.78)	<0.001
**Gout medications: Yes (ref: No)**	1.45 (0.86-2.45)	0.1621		
**Antiepileptics: Yes (ref: No)**	1.84 (1.64-2.05)	<0.001	1.57 (1.37-1.79)	<0.001
**Antipsychotics: Yes (ref: No)**	1.44 (1.30-1.58)	<0.001	1.25 (1.12-1.40)	<0.001
**inhaled anti-obstructive drugs: Yes (ref: No)**	0.87 (0.67-1.13)	0.2804		
**inhaled steroids: Yes (ref: No)**	0.52 (0.33-0.83)	0.0055	0.64 (0.39-1.05)	0.0760
**other COPD drugs: Yes (ref: No)**	0.83 (0.49-1.40)	0.4790		
**Systemic Antihistamines: Yes (ref: No)**	1.33 (1.14-1.55)	<0.001	1.32 (1.11-1.58)	0.0021

Table S18: Results of the simple and multivariable Cox regression models for all-cause mortality comparing propensity score matched COVID-19 and Influenza patients of age 65 to 74.

	Simple		Multivariable	
**Confounder**	**HR (CI)**	**p- value**	**HR (CI)**	**p- value**
**Group: Flu (ref: Covid)**	0.51 (0.42-0.61)	<0.001	0.50 (0.41-0.60)	<0.001
**Age Group: 65-70 (ref: 71-74)**	0.75 (0.64-0.88)	<0.001	0.76 (0.65-0.90)	0.0013
**Sex: W (ref: M)**	0.60 (0.53-0.67)	<0.001	0.59 (0.51-0.68)	<0.001
**Number Medication groups: 2-5 (ref: 0-1)**	1.35 (1.15-1.59)	<0.001	1.26 (1.06-1.49)	0.0082
**Number Medication groups: 6-10 (ref: 0-1)**	2.32 (1.92-2.81)	<0.001	1.73 (1.36-2.21)	<0.001
**Number Medication groups: >=11 (ref: 0-1)**	2.86 (2.30-3.55)	<0.001	1.57 (0.94-2.62)	0.0824
**Anticoagulants: Yes (ref: No)**	1.16 (0.97-1.39)	0.1034		
**Antibiotics, Antivirals, Antiprotozoals or Anthelmintics: Yes (ref: No)**	0.97 (0.86-1.08)	0.5636		
**Insulins and other Antidiabetics: Yes (ref: No)**	1.14 (1.01-1.29)	0.0298	1.24 (1.09-1.40)	<0.001
**"heart" drugs: Yes (ref: No)**	1.12 (0.98-1.27)	0.0995	1.20 (1.07-1.36)	0.0024
**Antihypertensives incl. Diuretics and Renin-angiotensin-aldosterone system inhibitors: Yes (ref: No)**	0.91 (0.76-1.08)	0.2793		
**Beta Blockers: Yes (ref: No)**	1.11 (0.90-1.37)	0.3195		
**Statins, Fibrates incl. Proprotein convertase subtilisin/kexin type 9 inhibitors and Inclisiran: Yes (ref: No)**	0.77 (0.72-0.83)	<0.001	0.81 (0.72-0.90)	<0.001
**Immunosuppressants and Immunomodulators: Yes (ref: No)**	1.04 (0.89-1.20)	0.6296		
**Systemic Steroids: Yes (ref: No)**	1.02 (0.89-1.17)	0.7785		
**Chemotherapy: Yes (ref: No)**	2.02 (1.70-2.40)	<0.001	1.79 (1.50-2.15)	<0.001
**Iron supplements, Erythropoietic stimulating agents, Vitamin B12, folic acid: Yes (ref: No)**	1.54 (1.31-1.81)	<0.001	1.34 (1.17-1.53)	<0.001
**Antacids incl. Antihistamines: Yes (ref: No)**	1.16 (1.00-1.34)	0.0444	1.19 (1.00-1.41)	0.0496
**Vitamin D and other Vitamin supplements: Yes (ref: No)**	1.05 (0.83-1.33)	0.7019		
**Caplacizumab: Yes (ref: No)**	NA (NA-NA)	NA		
**Systemic Hemostatics: Yes (ref: No)**	NA (NA-NA)	NA		
**Hereditary angioedema Therapeutics: Yes (ref: No)**	NA (NA-NA)	NA		
**Peripheral Vasodilators: Yes (ref: No)**	0.86 (0.60-1.23)	0.4146		
**Immunoglobulins: Yes (ref: No)**	NA (NA-NA)	NA		
**Interferons and CSF: Yes (ref: No)**	2.12 (1.83-2.46)	<0.001	1.97 (1.58-2.45)	<0.001
**NSAR and other anti-inflammatory drugs: Yes (ref: No)**	0.72 (0.64-0.80)	<0.001	0.77 (0.68-0.86)	<0.001
**Gout medications: Yes (ref: No)**	1.05 (0.86-1.28)	0.6369		
**Antiepileptics: Yes (ref: No)**	1.51 (1.29-1.78)	<0.001	1.39 (1.20-1.61)	<0.001
**Antipsychotics: Yes (ref: No)**	1.39 (1.25-1.54)	<0.001	1.32 (1.19-1.47)	<0.001
**inhaled anti-obstructive drugs: Yes (ref: No)**	1.04 (0.90-1.20)	0.6173		
**inhaled steroids: Yes (ref: No)**	0.71 (0.53-0.94)	0.0179	0.86 (0.66-1.12)	0.2533
**other COPD drugs: Yes (ref: No)**	0.58 (0.34-1.00)	0.0513	0.75 (0.47-1.22)	0.2509
**Systemic Antihistamines: Yes (ref: No)**	0.68 (0.57-0.81)	<0.001	0.72 (0.61-0.84)	<0.001

Table S19: Results of the simple and multivariable Cox regression models for all-cause mortality comparing propensity score matched COVID-19 and Influenza patients of age 75 and older.

	Simple		Multivariable	
**Confounder**	**HR (CI)**	**p- value**	**HR (CI)**	**p- value**
**Group: Flu (ref: Covid)**	0.51 (0.49-0.54)	<0.001	0.51 (0.49-0.53)	<0.001
**Age Group: 86-90 (ref: >= 91)**	0.62 (0.58-0.66)	<0.001	0.65 (0.60-0.70)	<0.001
**Age Group: 81-85 (ref: >= 91)**	0.42 (0.38-0.46)	<0.001	0.45 (0.41-0.49)	<0.001
**Age Group: 75-80 (ref: >= 91)**	0.28 (0.26-0.31)	<0.001	0.32 (0.28-0.36)	<0.001
**Sex: W (ref: M)**	0.70 (0.66-0.74)	<0.001	0.67 (0.64-0.71)	<0.001
**Number Medication groups: 2-5 (ref: 0-1)**	1.21 (1.1-1.33)	<0.001	1.04 (0.95-1.15)	0.3883
**Number Medication groups: 6-10 (ref: 0-1)**	1.52 (1.37-1.69)	<0.001	1.09 (0.96-1.24)	0.1911
**Number Medication groups: >=11 (ref: 0-1)**	1.99 (1.84-2.16)	<0.001	1.16 (1.05-1.27)	0.0029
**Anticoagulants: Yes (ref: No)**	1.10 (1.02-1.19)	0.0134	1.15 (1.08-1.23)	<0.001
**Antibiotics, Antivirals, Antiprotozoals or Anthelmintics: Yes (ref: No)**	1.14 (1.02-1.27)	0.0178	1.13 (1.03-1.24)	0.0112
**Insulins and other Antidiabetics: Yes (ref: No)**	1.06 (1.02-1.09)	0.0012	1.16 (1.13-1.19)	<0.001
**"heart" drugs: Yes (ref: No)**	1.04 (0.96-1.12)	0.3644		
**Antihypertensives incl. Diuretics and Renin-angiotensin-aldosterone system inhibitors: Yes (ref: No)**	0.89 (0.81-0.97)	0.0084	0.92 (0.85-1.00)	0.0485
**Beta Blockers: Yes (ref: No)**	1.01 (0.96-1.07)	0.6064		
**Statins, Fibrates incl. Proprotein convertase subtilisin/kexin type 9 inhibitors and Inclisiran: Yes (ref: No)**	0.80 (0.75-0.86)	<0.001	0.84 (0.79-0.89)	<0.001
**Immunosuppressants and Immunomodulators: Yes (ref: No)**	1.03 (0.91-1.17)	0.6093		
**Systemic Steroids: Yes (ref: No)**	0.98 (0.91-1.07)	0.7196		
**Chemotherapy: Yes (ref: No)**	1.08 (0.97-1.21)	0.1560		
**Iron supplements, Erythropoietic stimulating agents, Vitamin B12, folic acid: Yes (ref: No)**	1.41 (1.34-1.48)	<0.001	1.36 (1.31-1.42)	<0.001
**Antacids incl. Antihistamines: Yes (ref: No)**	1.08 (1.03-1.14)	0.0018	1.07 (1.02-1.12)	0.0026
**Vitamin D and other Vitamin supplements: Yes (ref: No)**	1.05 (0.99-1.13)	0.1247		
**Caplacizumab: Yes (ref: No)**	NA (NA-NA)	NA		
**Systemic Hemostatics: Yes (ref: No)**	1.94 (0.64-5.87)	0.2410		
**Hereditary angioedema Therapeutics: Yes (ref: No)**	NA (NA-NA)	NA		
**Peripheral Vasodilators: Yes (ref: No)**	0.69 (0.49-0.98)	0.0380	0.72 (0.52-0.99)	0.0448
**Immunoglobulins: Yes (ref: No)**	0.97 (0.25-3.80)	0.9703		
**Interferons and CSF: Yes (ref: No)**	1.32 (1.10-1.58)	0.0025	1.34 (1.12-1.60)	0.0013
**NSAR and other anti-inflammatory drugs: Yes (ref: No)**	0.75 (0.71-0.79)	<0.001	0.78 (0.74-0.82)	<0.001
**Gout medications: Yes (ref: No)**	1.08 (1.03-1.14)	0.0037	1.12 (1.07-1.18)	<0.001
**Antiepileptics: Yes (ref: No)**	1.05 (1.00-1.10)	0.0590	1.02 (0.98-1.05)	0.2838
**Antipsychotics: Yes (ref: No)**	1.41 (1.35-1.46)	<0.001	1.39 (1.33-1.45)	<0.001
**inhaled anti-obstructive drugs: Yes (ref: No)**	1.07 (1.01-1.14)	0.0234	1.07 (1.00-1.15)	0.0591
**inhaled steroids: Yes (ref: No)**	0.94 (0.82-1.07)	0.3133		
**other COPD drugs: Yes (ref: No)**	0.87 (0.62-1.22)	0.4216		
**Systemic Antihistamines: Yes (ref: No)**	0.98 (0.90-1.07)	0.6908		

Figure S1: Summary of Hazard ratios and 95%-confidence intervals for group, age, sex and medication group-effects comparing COVID-19 and Influenza patients for the outcome all-cause death in the age groups. Group: A hazard ratio larger than one is indicating a higher risk of the event in the Influenza group. Age: A hazard ratio larger than one is indicating a higher risk in younger age groups. Sex: A hazard ratio larger than one is indicating a higher risk in female patients. Medication Groups: a hazard ratio larger than one is indicating a higher risk in patients with a larger number of prescribed medication groups. Significant comparisons (with $p<0.05$) are marked with a blue star.

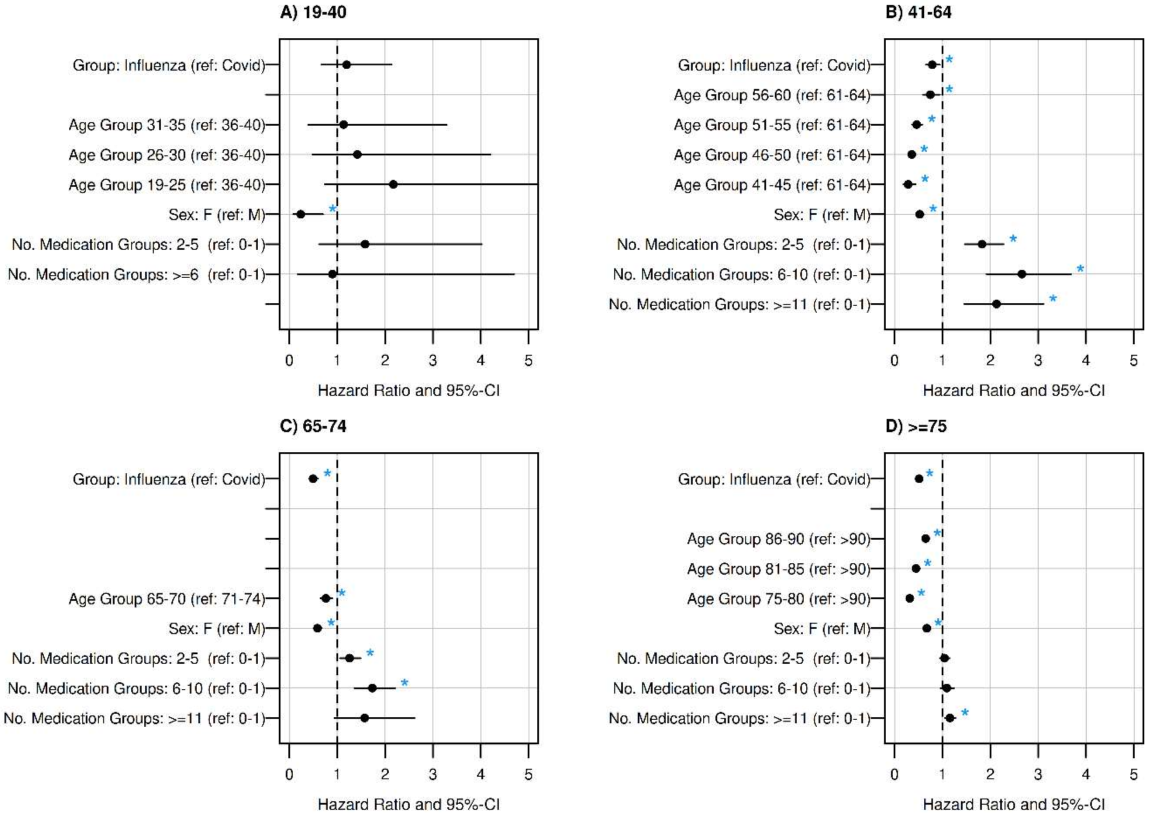

All-cause mortality within COVID-19 patient cohort

Table S20: Results of the simple and multivariable Cox regression for all-cause mortality models for COVID-19 patients of age 19 to 40.

Confounder	Simple		Multivariable	
**Confounder**	**HR (CI)**	**p- value**	**HR (CI)**	**p- value**
**Age Group: 31-35 (ref: 36-40)**	0.57 (0.21-1.59)	0.2836	0.77 (0.26-2.24)	0.6254
**Age Group: 26-30 (ref: 36-40)**	1.06 (0.60-1.88)	0.8302	1.44 (0.75-2.75)	0.2712
**Age Group: 19-25 (ref: 36-40)**	0.42 (0.12-1.42)	0.1621	0.55 (0.15-2.01)	0.3676
**Sex: W (ref: M)**	0.55 (0.43-0.70)	<0.001	0.54 (0.43-0.68)	<0.001
**Number Medication groups: 2-5 (ref: 0-1)**	1.64 (0.84-3.22)	0.1486	0.92 (0.56-1.50)	0.7332
**Number Medication groups: >=6 (ref: 0-1)**	8.53 (4.08-17.82)	<0.001	1.17 (0.28-4.90)	0.8330
**Wave: 3 (ref: Wave 1 & 2)**	0.89 (0.57-1.38)	0.5987	0.92 (0.58-1.47)	0.7396
**Wave: 4 (ref: Wave 1 & 2)**	0.82 (0.50-1.36)	0.4461	0.82 (0.55-1.23)	0.3390
**Anticoagulants: Yes (ref: No)**	1.30 (0.58-2.95)	0.5254		
**Antibiotics, Antivirals, Antiprotozoals or Anthelmintics: Yes (ref: No)**	0.68 (0.51-0.92)	0.0114	1.03 (0.68-1.56)	0.8982
**Insulins and other Antidiabetics: Yes (ref: No)**	5.91 (3.51-9.96)	<0.001	5.57 (2.14-14.48)	<0.001
**"heart" drugs: Yes (ref: No)**	NA (NA-NA)	NA		
**Antihypertensives incl. Diuretics and Renin-angiotensin-aldosterone system inhibitors: Yes (ref: No)**	2.47 (0.96-6.36)	0.0617	1.64 (0.51-5.25)	0.4057
**Beta Blockers: Yes (ref: No)**	2.04 (0.99-4.20)	0.0525	0.99 (0.48-2.04)	0.9718
**Statins, Fibrates incl. Proprotein convertase subtilisin/kexin type 9 inhibitors and Inclisiran: Yes (ref: No)**	1.74 (0.81-3.74)	0.1538		
**Immunosuppressants and Immunomodulators: Yes (ref: No)**	1.15 (0.49-2.73)	0.7486		
**Systemic Steroids: Yes (ref: No)**	1.73 (0.97-3.09)	0.0637	2.49 (1.45-4.26)	<0.001
**Chemotherapy: Yes (ref: No)**	NA (NA-NA)	NA		
**Iron supplements, Erythropoietic stimulating agents, Vitamin B12, folic acid: Yes (ref: No)**	0.66 (0.38-1.13)	0.1327		
**Antacids incl. Antihistamines: Yes (ref: No)**	1.69 (1.01-2.81)	0.0448	1.47 (0.82-2.66)	0.1970
**Vitamin D and other Vitamin supplements: Yes (ref: No)**	0.67 (0.27-1.67)	0.3939		
**Caplacizumab: Yes (ref: No)**	NA (NA-NA)	NA		
**Systemic Hemostatics: Yes (ref: No)**	NA (NA-NA)	NA		
**Hereditary angioedema Therapeutics: Yes (ref: No)**	NA (NA-NA)	NA		
**Peripheral Vasodilators: Yes (ref: No)**	NA (NA-NA)	NA		
**Immunoglobulins: Yes (ref: No)**	NA (NA-NA)	NA		
**Interferons and CSF: Yes (ref: No)**	0.81 (0.09-7.50)	0.8546		
**NSAR and other anti-inflammatory drugs: Yes (ref: No)**	0.45 (0.24-0.85)	0.0132	0.63 (0.36-1.12)	0.1142
**Gout medications: Yes (ref: No)**	NA (NA-NA)	NA		
**Antiepileptics: Yes (ref: No)**	2.50 (1.31-4.76)	0.0054	1.81 (0.78-4.24)	0.1699
**Antipsychotics: Yes (ref: No)**	2.32 (1.13-4.75)	0.0215	2.07 (1.06-4.05)	0.0337
**inhaled anti-obstructive drugs: Yes (ref: No)**	1.22 (0.77-1.93)	0.3955		
**inhaled steroids: Yes (ref: No)**	0.77 (0.29-2.01)	0.5890		
**other COPD drugs: Yes (ref: No)**	NA (NA-NA)	NA		
**Systemic Antihistamines: Yes (ref: No)**	0.77 (0.35-1.71)	0.5248		

Table S21: Results of the simple and multivariable Cox regression models for all-cause mortality for COVID-19 patients of age 41 to 64.

	Simple		Multivariable	
**Confounder**	**HR (CI)**	**p- value**	**HR (CI)**	**p- value**
**Age Group: 56-60 (ref: 61-64)**	0.63 (0.55-0.72)	<0.001	0.63 (0.55-0.73)	<0.001
**Age Group: 51-55 (ref: 61-64)**	0.43 (0.34-0.54)	<0.001	0.43 (0.34-0.53)	<0.001
**Age Group: 46-50 (ref: 61-64)**	0.33 (0.27-0.40)	<0.001	0.31 (0.26-0.37)	<0.001
**Age Group: 41-55 (ref: 61-64)**	0.19 (0.15-0.26)	<0.001	0.19 (0.15-0.24)	<0.001
**Sex: W (ref: M)**	0.64 (0.57-0.72)	<0.001	0.60 (0.54-0.67)	<0.001
**Number Medication groups: 2-5 (ref: 0-1)**	1.84 (1.57-2.16)	<0.001	1.64 (1.43-1.88)	<0.001
**Number Medication groups: 6-10 (ref: 0-1)**	4.18 (3.64-4.81)	<0.001	2.57 (2.03-3.24)	<0.001
**Number Medication groups: >=11 (ref: 0-1)**	6.00 (4.56-7.89)	<0.001	2.34 (1.50-3.64)	<0.001
**Wave: 3 (ref: Wave 1 & 2)**	0.90 (0.80-1.02)	0.0973	0.94 (0.82-1.08)	0.3984
**Wave: 4 (ref: Wave 1 & 2)**	1.27 (1.14-1.4)	<0.001	1.29 (1.15-1.43)	<0.001
**Anticoagulants: Yes (ref: No)**	1.09 (0.96-1.22)	0.1717		
**Antibiotics, Antivirals, Antiprotozoals or Anthelmintics: Yes (ref: No)**	1.13 (1.03-1.25)	0.0105	1.13 (1.02-1.24)	0.0186
**Insulins and other Antidiabetics: Yes (ref: No)**	0.97 (0.90-1.04)	0.3796		
**"heart" drugs: Yes (ref: No)**	0.81 (0.62-1.07)	0.1360		
**Antihypertensives incl. Diuretics and Renin-angiotensin-aldosterone system inhibitors: Yes (ref: No)**	1.09 (0.97-1.23)	0.1330		
**Beta Blockers: Yes (ref: No)**	1.10 (0.91-1.32)	0.3302		
**Statins, Fibrates incl. Proprotein convertase subtilisin/kexin type 9 inhibitors and Inclisiran: Yes (ref: No)**	0.69 (0.62-0.78)	<0.001	0.86 (0.76-0.98)	0.0277
**Immunosuppressants and Immunomodulators: Yes (ref: No)**	1.02 (0.88-1.19)	0.7552		
**Systemic Steroids: Yes (ref: No)**	1.36 (1.21-1.54)	<0.001	1.31 (1.19-1.44)	<0.001
**Chemotherapy: Yes (ref: No)**	2.63 (2.08-3.33)	<0.001	1.97 (1.59-2.45)	<0.001
**Iron supplements, Erythropoietic stimulating agents, Vitamin B12, folic acid: Yes (ref: No)**	1.88 (1.55-2.27)	<0.001	1.53 (1.27-1.84)	<0.001
**Antacids incl. Antihistamines: Yes (ref: No)**	1.08 (0.96-1.21)	0.1880		
**Vitamin D and other Vitamin supplements: Yes (ref: No)**	1.46 (1.16-1.84)	0.0015	1.35 (1.11-1.63)	0.0024
**Caplacizumab: Yes (ref: No)**	NA (NA-NA)	NA		
**Systemic Hemostatics: Yes (ref: No)**	4.33 (0.63-29.78)	0.1362		
**Hereditary angioedema Therapeutics: Yes (ref: No)**	NA (NA-NA)	NA		
**Peripheral Vasodilators: Yes (ref: No)**	0.49 (0.20-1.19)	0.1152		
**Immunoglobulins: Yes (ref: No)**	2.11 (0.39-11.48)	0.3895		
**Interferons and CSF: Yes (ref: No)**	2.91 (2.38-3.55)	<0.001	2.18 (1.79-2.65)	<0.001
**NSAR and other anti-inflammatory drugs: Yes (ref: No)**	0.67 (0.61-0.75)	<0.001	0.73 (0.66-0.80)	<0.001
**Gout medications: Yes (ref: No)**	1.06 (0.85-1.33)	0.5842		
**Antiepileptics: Yes (ref: No)**	1.85 (1.58-2.15)	<0.001	1.61 (1.41-1.83)	<0.001
**Antipsychotics: Yes (ref: No)**	1.42 (1.22-1.65)	<0.001	1.31 (1.16-1.48)	<0.001
**inhaled anti-obstructive drugs: Yes (ref: No)**	0.92 (0.75-1.14)	0.4592		
**inhaled steroids: Yes (ref: No)**	0.75 (0.64-0.88)	<0.001	0.87 (0.75-1.00)	0.0562
**other COPD drugs: Yes (ref: No)**	0.61 (0.39-0.95)	0.0273	0.71 (0.48-1.05)	0.0890
**Systemic Antihistamines: Yes (ref: No)**	1.06 (0.92-1.22)	0.4239		

Table S22: Results of the simple and multivariable Cox regression for all-cause mortality models for COVID-19 patients of age 65 to 74.

	Simple		Multivariable	
**Confounder**	**HR (CI)**	**p- value**	**HR (CI)**	**p- value**
**Age Group: 65-70 (ref: 71-74)**	0.74 (0.71-0.78)	<0.001	0.76 (0.73-0.79)	<0.001
**Sex: W (ref: M)**	0.58 (0.54-0.62)	<0.001	0.56 (0.51-0.61)	<0.001
**Number Medication groups: 2-5 (ref: 0-1)**	1.24 (1.11-1.39)	<0.001	1.13 (1.01-1.27)	0.0401
**Number Medication groups: 6-10 (ref: 0-1)**	2.09 (1.80-2.43)	<0.001	1.33 (1.12-1.58)	0.0011
**Number Medication groups: >=11 (ref: 0-1)**	2.84 (2.33-3.46)	<0.001	1.21 (0.92-1.61)	0.1791
**Wave: 3 (ref: Wave 1 & 2)**	0.93 (0.87-1.00)	0.0584	0.96 (0.90-1.02)	0.1516
**Wave: 4 (ref: Wave 1 & 2)**	1.22 (1.08-1.37)	0.0014	1.21 (1.08-1.36)	0.0010
**Anticoagulants: Yes (ref: No)**	1.13 (1.04-1.22)	0.0050	1.17 (1.07-1.28)	<0.001
**Antibiotics, Antivirals, Antiprotozoals or Anthelmintics: Yes (ref: No)**	0.99 (0.88-1.11)	0.8455	1.02 (0.91-1.15)	0.7042
**Insulins and other Antidiabetics: Yes (ref: No)**	1.13 (1.05-1.22)	0.0017	1.28 (1.18-1.39)	<0.001
**"heart" drugs: Yes (ref: No)**	0.98 (0.88-1.1)	0.7688		
**Antihypertensives incl. Diuretics and Renin-angiotensin-aldosterone system inhibitors: Yes (ref: No)**	0.89 (0.80-0.98)	0.0207	0.95 (0.87-1.05)	0.3224
**Beta Blockers: Yes (ref: No)**	1.09 (0.99-1.21)	0.0781	1.14 (1.09-1.20)	<0.001
**Statins, Fibrates incl. Proprotein convertase subtilisin/kexin type 9 inhibitors and Inclisiran: Yes (ref: No)**	0.74 (0.69-0.80)	<0.001	0.77 (0.72-0.83)	<0.001
**Immunosuppressants and Immunomodulators: Yes (ref: No)**	1.16 (1.00-1.33)	0.0442	1.09 (0.94-1.25)	0.2618
**Systemic Steroids: Yes (ref: No)**	1.14 (1.07-1.21)	<0.001	1.20 (1.10-1.31)	<0.001
**Chemotherapy: Yes (ref: No)**	1.74 (1.50-2.01)	<0.001	1.66 (1.37-2.00)	<0.001
**Iron supplements, Erythropoietic stimulating agents, Vitamin B12, folic acid: Yes (ref: No)**	1.62 (1.34-1.94)	<0.001	1.48 (1.26-1.75)	<0.001
**Antacids incl. Antihistamines: Yes (ref: No)**	1.12 (1.04-1.21)	0.0031	1.14 (1.06-1.22)	<0.001
**Vitamin D and other Vitamin supplements: Yes (ref: No)**	1.10 (1.03-1.18)	0.0072	1.06 (0.97-1.15)	0.1902
**Caplacizumab: Yes (ref: No)**	NA (NA-NA)	NA		
**Systemic Hemostatics: Yes (ref: No)**	1.18 (0.13-10.44)	0.8807		
**Hereditary angioedema Therapeutics: Yes (ref: No)**	NA (NA-NA)	NA		
**Peripheral Vasodilators: Yes (ref: No)**	1.12 (0.86-1.47)	0.3930		
**Immunoglobulins: Yes (ref: No)**	0.38 (0.06-2.39)	0.3024		
**Interferons and CSF: Yes (ref: No)**	1.79 (1.56-2.04)	<0.001	1.62 (1.40-1.87)	<0.001
**NSAR and other anti-inflammatory drugs: Yes (ref: No)**	0.67 (0.64-0.70)	<0.001	0.72 (0.69-0.75)	<0.001
**Gout medications: Yes (ref: No)**	1.06 (0.87-1.29)	0.5587		
**Antiepileptics: Yes (ref: No)**	1.34 (1.19-1.51)	<0.001	1.28 (1.14-1.45)	<0.001
**Antipsychotics: Yes (ref: No)**	1.35 (1.26-1.44)	<0.001	1.32 (1.23-1.42)	<0.001
**inhaled anti-obstructive drugs: Yes (ref: No)**	1.01 (0.96-1.06)	0.7535		
**inhaled steroids: Yes (ref: No)**	0.77 (0.67-0.88)	<0.001	0.88 (0.77-1)	0.0440
**other COPD drugs: Yes (ref: No)**	0.76 (0.52-1.10)	0.1504		
**Systemic Antihistamines: Yes (ref: No)**	0.85 (0.75-0.96)	0.0095	0.90 (0.80-1.01)	0.0617

Table S23: Results of the simple and multivariable Cox regression for all-cause mortality models for COVID-19 patients of age 75 and older.

	Simple		Multivariable	
**Confounder**	**HR (CI)**	**p- value**	**HR (CI)**	**p- value**
**Age Group: 86-90 (ref: >= 91)**	0.64 (0.59-0.69)	<0.001	0.67 (0.62-0.73)	<0.001
**Age Group: 81-85 (ref: >= 91)**	0.43 (0.4-0.45)	<0.001	0.46 (0.43-0.49)	<0.001
**Age Group: 75-80 (ref: >= 91)**	0.31 (0.29-0.33)	<0.001	0.35 (0.32-0.37)	<0.001
**Sex: W (ref: M)**	0.73 (0.7-0.75)	<0.001	0.69 (0.66-0.72)	<0.001
**Number Medication groups: 2-5 (ref: 0-1)**	1.09 (1-1.19)	0.0586	0.93 (0.84-1.04)	0.2236
**Number Medication groups: 6-10 (ref: 0-1)**	1.37 (1.24-1.52)	<0.001	0.95 (0.81-1.11)	0.5206
**Number Medication groups: >=11 (ref: 0-1)**	1.73 (1.51-1.97)	<0.001	0.95 (0.79-1.13)	0.5361
**Wave: 3 (ref: Wave 1 & 2)**	0.97 (0.89-1.07)	0.5734	0.99 (0.9-1.08)	0.7492
**Wave: 4 (ref: Wave 1 & 2)**	0.97 (0.85-1.11)	0.6641	1.00 (0.88-1.14)	0.9950
**Anticoagulants: Yes (ref: No)**	1.13 (1.09-1.17)	<0.001	1.15 (1.11-1.18)	<0.001
**Antibiotics, Antivirals, Antiprotozoals or Anthelmintics: Yes (ref: No)**	1.14 (1.05-1.23)	0.0010	1.15 (1.06-1.25)	<0.001
**Insulins and other Antidiabetics: Yes (ref: No)**	1.07 (1-1.13)	0.0346	1.15 (1.09-1.21)	<0.001
**"heart" drugs: Yes (ref: No)**	1.05 (1.01-1.08)	0.0068	1.08 (1.05-1.12)	<0.001
**Antihypertensives incl. Diuretics and Renin-angiotensin-aldosterone system inhibitors: Yes (ref: No)**	0.89 (0.83-0.96)	0.0034	0.93 (0.86-1.00)	0.0547
**Beta Blockers: Yes (ref: No)**	1.03 (1-1.07)	0.0730	1.07 (1.03-1.11)	<0.001
**Statins, Fibrates incl. Proprotein convertase subtilisin/kexin type 9 inhibitors and Inclisiran: Yes (ref: No)**	0.81 (0.78-0.85)	<0.001	0.85 (0.82-0.88)	<0.001
**Immunosuppressants and Immunomodulators: Yes (ref: No)**	1.04 (0.93-1.16)	0.5067		
**Systemic Steroids: Yes (ref: No)**	0.98 (0.88-1.09)	0.6804		
**Chemotherapy: Yes (ref: No)**	1.06 (0.98-1.15)	0.1365		
**Iron supplements, Erythropoietic stimulating agents, Vitamin B12, folic acid: Yes (ref: No)**	1.40 (1.32-1.48)	<0.001	1.36 (1.3-1.44)	<0.001
**Antacids incl. Antihistamines: Yes (ref: No)**	1.05 (1.03-1.07)	<0.001	1.03 (1.01-1.05)	<0.001
**Vitamin D and other Vitamin supplements: Yes (ref: No)**	1.06 (1.01-1.10)	0.0102	1.01 (0.97-1.05)	0.5704
**Caplacizumab: Yes (ref: No)**	NA (NA-NA)	NA		
**Systemic Hemostatics: Yes (ref: No)**	1.85 (0.93-3.7)	0.0809	1.89 (0.94-3.79)	0.0738
**Hereditary angioedema Therapeutics: Yes (ref: No)**	NA (NA-NA)	NA		
**Peripheral Vasodilators: Yes (ref: No)**	0.83 (0.72-0.97)	0.0163	0.90 (0.77-1.05)	0.1809
**Immunoglobulins: Yes (ref: No)**	3.32 (2.18-5.06)	<0.001	3.19 (2.3-4.43)	<0.001
**Interferons and CSF: Yes (ref: No)**	1.15 (1.06-1.25)	<0.001	1.26 (1.17-1.36)	<0.001
**NSAR and other anti-inflammatory drugs: Yes (ref: No)**	0.76 (0.72-0.79)	<0.001	0.80 (0.77-0.83)	<0.001
**Gout medications: Yes (ref: No)**	1.03 (0.95-1.13)	0.4473		
**Antiepileptics: Yes (ref: No)**	1.08 (1.05-1.12)	<0.001	1.06 (1.02-1.1)	0.0025
**Antipsychotics: Yes (ref: No)**	1.37 (1.31-1.42)	<0.001	1.34 (1.3-1.39)	<0.001
**inhaled anti-obstructive drugs: Yes (ref: No)**	1.03 (0.99-1.07)	0.1357		
**inhaled steroids: Yes (ref: No)**	0.87 (0.8-0.95)	0.0026	0.93 (0.84-1.02)	0.1150
**other COPD drugs: Yes (ref: No)**	0.89 (0.72-1.1)	0.2728		
**Systemic Antihistamines: Yes (ref: No)**	0.99 (0.92-1.06)	0.7079		

All-cause mortality within Influenza patient cohort

Table S24: Results of the simple and multivariable Cox regression for all-cause mortality models for Influenza patients of age 19 to 40.

	Simple		Multivariable	
**Confounder**	**HR (CI)**	**p- value**	**HR (CI)**	**p- value**
**Age Group: 31-35 (ref: 36-40)**	1.01 (0.5-2.05)	0.9718	1.03 (0.40-2.68)	0.9482
**Age Group: 26-30 (ref: 36-40)**	1.00 (0.35-2.87)	0.9975	1.44 (0.49-4.26)	0.5060
**Age Group: 19-25 (ref: 36-40)**	1.12 (0.61-2.06)	0.7226	1.69 (0.80-3.56)	0.1676
**Sex: W (ref: M)**	0.55 (0.28-1.06)	0.0751	0.83 (0.31-2.26)	0.7205
**Number Medication groups: 2-5 (ref: 0-1)**	4.26 (1.42-12.78)	0.0097	2.49 (0.53-11.60)	0.2470
**Number Medication groups: >=6 (ref: 0-1)**	27.65 (7.52-101.7)	<0.001	5.02 (0.8-31.73)	0.0860
**Wave: 2016/2017 (ref: Wave 2015/2016)**	NA (NA-NA)	NA	NA (NA-NA)	NA
**Wave: 2017/2018 (ref: Wave 2015/2016)**	NA (NA-NA)	NA	NA (NA-NA)	NA
**Wave: 2018/2019 (ref: Wave 2015/2016)**	NA (NA-NA)	NA	NA (NA-NA)	NA
**Wave: 2019/2020 (ref: Wave 2015/2016)**	NA (NA-NA)	NA	NA (NA-NA)	NA
**Wave: 2020/2021 (ref: Wave 2015/2016)**	0.99 (0.15-6.31)	0.9882	1.17 (0.2-6.96)	0.8638
**Anticoagulants: Yes (ref: No)**	2.80 (1.72-4.56)	<0.001	1.56 (0.63-3.89)	0.3404
**Antibiotics, Antivirals, Antiprotozoals or Anthelmintics: Yes (ref: No)**	1.25 (0.6-2.59)	0.5464		
**Insulins and other Antidiabetics: Yes (ref: No)**	0.84 (0.28-2.49)	0.7545		
**"heart" drugs: Yes (ref: No)**	6.01 (3.27-11.03)	<0.001		
**Antihypertensives incl. Diuretics and Renin-angiotensin-aldosterone system inhibitors: Yes (ref: No)**	2.48 (1.32-4.68)	0.0048	1.84 (0.50-6.82)	0.3608
**Beta Blockers: Yes (ref: No)**	2.14 (1.38-3.31)	<0.001	0.88 (0.22-3.59)	0.8605
**Statins, Fibrates incl. Proprotein convertase subtilisin/kexin type 9 inhibitors and Inclisiran: Yes (ref: No)**	0.17 (0.02-1.44)	0.1048		
**Immunosuppressants and Immunomodulators: Yes (ref: No)**	0.86 (0.51-1.45)	0.5658		
**Systemic Steroids: Yes (ref: No)**	0.46 (0.2-1.02)	0.0552	0.63 (0.19-2.10)	0.4499
**Chemotherapy: Yes (ref: No)**	3.04 (1.22-7.57)	0.0167	2.88 (0.70-11.76)	0.1415
**Iron supplements, Erythropoietic stimulating agents, Vitamin B12, folic acid: Yes (ref: No)**	1.49 (0.76-2.95)	0.2475		
**Antacids incl. Antihistamines: Yes (ref: No)**	4.01 (1.99-8.08)	<0.001	4.05 (1.69-9.71)	0.0017
**Vitamin D and other Vitamin supplements: Yes (ref: No)**	0.66 (0.39-1.12)	0.1214		
**Caplacizumab: Yes (ref: No)**	NA (NA-NA)	NA		
**Systemic Hemostatics: Yes (ref: No)**	NA (NA-NA)	NA		
**Hereditary angioedema Therapeutics: Yes (ref: No)**	NA (NA-NA)	NA		
**Peripheral Vasodilators: Yes (ref: No)**	NA (NA-NA)	NA		
**Immunoglobulins: Yes (ref: No)**	NA (NA-NA)	NA		
**Interferons and CSF: Yes (ref: No)**	NA (NA-NA)	NA		
**NSAR and other anti-inflammatory drugs: Yes (ref: No)**	0.62 (0.40-0.95)	0.0287	0.65 (0.35-1.20)	0.1658
**Gout medications: Yes (ref: No)**	NA (NA-NA)	NA		
**Antiepileptics: Yes (ref: No)**	3.16 (1.40-7.15)	0.0056	3.08 (1.02-9.33)	0.0461
**Antipsychotics: Yes (ref: No)**	2.67 (1.41-5.03)	0.0025	1.50 (0.53-4.26)	0.4479
**inhaled anti-obstructive drugs: Yes (ref: No)**	1.08 (0.71-1.63)	0.7168		
**inhaled steroids: Yes (ref: No)**	2.44 (0.67-8.93)	0.1765		
**other COPD drugs: Yes (ref: No)**	0.44 (0.05-4.10)	0.4673		
**Systemic Antihistamines: Yes (ref: No)**	0.23 (0.10-0.53)	<0.001	0.38 (0.20-0.72)	0.0030

Table S25: Results of the simple and multivariable Cox regression models for all-cause mortality of Influenza patients of age 41 to 64.

	Simple		Multivariable	
**Confounder**	**HR (CI)**	**p- value**	**HR (CI)**	**p- value**
**Age Group: 56-60 (ref: 61-64)**	0.78 (0.66-0.93)	0.0055	0.72 (0.62-0.84)	<0.001
**Age Group: 51-55 (ref: 61-64)**	0.57 (0.49-0.66)	<0.001	0.48 (0.42-0.56)	<0.001
**Age Group: 46-50 (ref: 61-64)**	0.51 (0.34-0.76)	0.0011	0.43 (0.3-0.63)	<0.001
**Age Group: 41-55 (ref: 61-64)**	0.61 (0.45-0.83)	0.0014	0.55 (0.41-0.72)	<0.001
**Sex: W (ref: M)**	0.50 (0.42-0.60)	<0.001	0.46 (0.37-0.57)	<0.001
**Number Medication groups: 2-5 (ref: 0-1)**	2.25 (1.58-3.19)	<0.001	1.92 (1.31-2.80)	<0.001
**Number Medication groups: 6-10 (ref: 0-1)**	4.71 (3.69-6.01)	<0.001	2.90 (1.97-4.25)	<0.001
**Number Medication groups: >=11 (ref: 0-1)**	6.45 (4.75-8.75)	<0.001	2.30 (1.29-4.07)	0.0045
**Wave: 2016/2017 (ref: Wave 2015/2016)**	0.69 (0.55-0.86)	0.0013	0.72 (0.57-0.91)	0.0054
**Wave: 2017/2018 (ref: Wave 2015/2016)**	0.66 (0.43-1.01)	0.0579	0.67 (0.4-1.13)	0.1374
**Wave: 2018/2019 (ref: Wave 2015/2016)**	0.69 (0.44-1.11)	0.1242	0.72 (0.44-1.18)	0.1927
**Wave: 2019/2020 (ref: Wave 2015/2016)**	0.72 (0.40-1.31)	0.2814	0.71 (0.37-1.35)	0.2976
**Wave: 2020/2021 (ref: Wave 2015/2016)**	7.54 (2.95-19.28)	<0.001	6.42 (3.37-12.20)	<0.001
**Anticoagulants: Yes (ref: No)**	1.21 (0.99-1.47)	0.0566	1.46 (1.20-1.79)	<0.001
**Antibiotics, Antivirals, Antiprotozoals or Anthelmintics: Yes (ref: No)**	0.93 (0.77-1.13)	0.4631		
**Insulins and other Antidiabetics: Yes (ref: No)**	1.07 (0.91-1.26)	0.4070		
**"heart" drugs: Yes (ref: No)**	1.21 (0.84-1.73)	0.2981		
**Antihypertensives incl. Diuretics and Renin-angiotensin-aldosterone system inhibitors: Yes (ref: No)**	1.21 (0.93-1.58)	0.1527		
**Beta Blockers: Yes (ref: No)**	0.87 (0.74-1.01)	0.0698	0.88 (0.71-1.09)	0.2464
**Statins, Fibrates incl. Proprotein convertase subtilisin/kexin type 9 inhibitors and Inclisiran: Yes (ref: No)**	0.56 (0.46-0.69)	<0.001	0.72 (0.55-0.94)	0.0153
**Immunosuppressants and Immunomodulators: Yes (ref: No)**	1.15 (0.84-1.57)	0.3786		
**Systemic Steroids: Yes (ref: No)**	1.29 (1.07-1.56)	0.0081	1.36 (1.08-1.72)	0.0094
**Chemotherapy: Yes (ref: No)**	3.06 (1.71-5.47)	<0.001	2.92 (1.83-4.66)	<0.001
**Iron supplements, Erythropoietic stimulating agents, Vitamin B12, folic acid: Yes (ref: No)**	2.26 (1.67-3.06)	<0.001	1.79 (1.29-2.48)	<0.001
**Antacids incl. Antihistamines: Yes (ref: No)**	1.01 (0.74-1.39)	0.9437		
**Vitamin D and other Vitamin supplements: Yes (ref: No)**	1.57 (1.33-1.84)	<0.001	1.33 (1.11-1.58)	0.0016
**Caplacizumab: Yes (ref: No)**	NA (NA-NA)	NA		
**Systemic Hemostatics: Yes (ref: No)**	2.33 (1.16-4.70)	0.0179	2.02 (0.58-7.02)	0.2697
**Hereditary angioedema Therapeutics: Yes (ref: No)**	NA (NA-NA)	NA		
**Peripheral Vasodilators: Yes (ref: No)**	0.65 (0.23-1.83)	0.4160		
**Immunoglobulins: Yes (ref: No)**	NA (NA-NA)	NA		
**Interferons and CSF: Yes (ref: No)**	1.61 (0.97-2.67)	0.0677	1.32 (0.88-1.99)	0.1785
**NSAR and other anti-inflammatory drugs: Yes (ref: No)**	0.59 (0.49-0.70)	<0.001	0.64 (0.52-0.78)	<0.001
**Gout medications: Yes (ref: No)**	1.38 (0.85-2.23)	0.1936		
**Antiepileptics: Yes (ref: No)**	1.97 (1.50-2.57)	<0.001	1.66 (1.28-2.16)	<0.001
**Antipsychotics: Yes (ref: No)**	1.48 (1.29-1.69)	<0.001	1.27 (1.07-1.51)	0.0067
**inhaled anti-obstructive drugs: Yes (ref: No)**	0.88 (0.68-1.14)	0.3394		
**inhaled steroids: Yes (ref: No)**	0.62 (0.43-0.90)	0.0110	0.80 (0.57-1.11)	0.1826
**other COPD drugs: Yes (ref: No)**	0.65 (0.53-0.79)	<0.001	0.73 (0.56-0.94)	0.0161
**Systemic Antihistamines: Yes (ref: No)**	1.46 (1.19-1.8)	<0.001	1.45 (1.00-2.10)	0.0491

Table S26: Results of the simple and multivariable Cox regression models for all-cause mortality of Influenza patients of age 65 to 74.

	Simple		Multivariable	
**Confounder**	**HR (CI)**	**p- value**	**HR (CI)**	**p- value**
**Age Group: 65-70 (ref: 71-74)**	0.76 (0.65-0.88)	<0.001	0.77 (0.65-0.91)	0.0021
**Sex: W (ref: M)**	0.66 (0.54-0.80)	<0.001	0.64 (0.49-0.84)	0.0013
**Number Medication groups: 2-5 (ref: 0-1)**	1.58 (1.19-2.11)	0.0018	1.38 (1.02-1.86)	0.0356
**Number Medication groups: 6-10 (ref: 0-1)**	2.91 (2.27-3.74)	<0.001	2.14 (1.59-2.88)	<0.001
**Number Medication groups: >=11 (ref: 0-1)**	3.33 (2.56-4.33)	<0.001	1.79 (1.24-2.59)	0.0018
**Wave: 2016/2017 (ref: Wave 2015/2016)**	0.79 (0.57-1.10)	0.1619	0.78 (0.55-1.10)	0.1525
**Wave: 2017/2018 (ref: Wave 2015/2016)**	0.84 (0.57-1.24)	0.3785	0.83 (0.58-1.19)	0.3039
**Wave: 2018/2019 (ref: Wave 2015/2016)**	0.70 (0.53-0.93)	0.0149	0.69 (0.52-0.91)	0.0088
**Wave: 2019/2020 (ref: Wave 2015/2016)**	0.92 (0.64-1.31)	0.6322	0.9 (0.63-1.29)	0.5738
**Wave: 2020/2021 (ref: Wave 2015/2016)**	1.56 (0.80-3.03)	0.1933	1.65 (0.93-2.94)	0.0896
**Anticoagulants: Yes (ref: No)**	1.16 (0.89-1.50)	0.2727		
**Antibiotics, Antivirals, Antiprotozoals or Anthelmintics: Yes (ref: No)**	1.09 (0.89-1.33)	0.3958		
**Insulins and other Antidiabetics: Yes (ref: No)**	1.26 (1.12-1.41)	<0.001	1.41 (1.24-1.61)	<0.001
**"heart" drugs: Yes (ref: No)**	1.12 (0.85-1.48)	0.4047		
**Antihypertensives incl. Diuretics and Renin-angiotensin-aldosterone system inhibitors: Yes (ref: No)**	0.90 (0.79-1.02)	0.0921	1.01 (0.92-1.11)	0.7818
**Beta Blockers: Yes (ref: No)**	1.09 (0.86-1.38)	0.4887		
**Statins, Fibrates incl. Proprotein convertase subtilisin/kexin type 9 inhibitors and Inclisiran: Yes (ref: No)**	0.71 (0.62-0.81)	<0.001	0.75 (0.66-0.86)	<0.001
**Immunosuppressants and Immunomodulators: Yes (ref: No)**	0.96 (0.65-1.43)	0.8485		
**Systemic Steroids: Yes (ref: No)**	1.19 (0.98-1.44)	0.0737	1.24 (1.06-1.45)	0.0087
**Chemotherapy: Yes (ref: No)**	2.75 (1.72-4.38)	<0.001	2.46 (1.54-3.91)	<0.001
**Iron supplements, Erythropoietic stimulating agents, Vitamin B12, folic acid: Yes (ref: No)**	1.49 (1.12-1.99)	0.0066	1.34 (1.04-1.73)	0.0225
**Antacids incl. Antihistamines: Yes (ref: No)**	1.15 (0.93-1.42)	0.2056		
**Vitamin D and other Vitamin supplements: Yes (ref: No)**	0.98 (0.64-1.51)	0.9301		
**Caplacizumab: Yes (ref: No)**	NA (NA-NA)	NA		
**Systemic Hemostatics: Yes (ref: No)**	NA (NA-NA)	NA		
**Hereditary angioedema Therapeutics: Yes (ref: No)**	NA (NA-NA)	NA		
**Peripheral Vasodilators: Yes (ref: No)**	0.86 (0.60-1.24)	0.4283		
**Immunoglobulins: Yes (ref: No)**	NA (NA-NA)	NA		
**Interferons and CSF: Yes (ref: No)**	2.45 (2.01-2.99)	<0.001	2.07 (1.7-2.53)	<0.001
**NSAR and other anti-inflammatory drugs: Yes (ref: No)**	0.83 (0.70-0.98)	0.0319	0.88 (0.75-1.02)	0.0817
**Gout medications: Yes (ref: No)**	1.06 (0.83-1.35)	0.6404		
**Antiepileptics: Yes (ref: No)**	1.39 (1.18-1.64)	<0.001	1.28 (1.13-1.45)	<0.001
**Antipsychotics: Yes (ref: No)**	1.34 (1.21-1.49)	<0.001	1.39 (1.23-1.57)	<0.001
**inhaled anti-obstructive drugs: Yes (ref: No)**	1.16 (0.95-1.43)	0.1530		
**inhaled steroids: Yes (ref: No)**	0.82 (0.75-0.90)	<0.001	0.94 (0.84-1.05)	0.3086
**other COPD drugs: Yes (ref: No)**	1.26 (0.91-1.76)	0.1669		
**Systemic Antihistamines: Yes (ref: No)**	0.71 (0.61-0.83)	<0.001	0.78 (0.64-0.95)	0.0124

Table S27: Results of the simple and multivariable Cox regression models for all-cause mortality of Influenza patients of age 75 and older.

	Simple		Multivariable	
Confounder	HR (CI)	p- value	HR (CI)	p- value
Age Group: 86-90 (ref: >= 91)	0.56 (0.51-0.62)	<0.001	0.58 (0.54-0.63)	<0.001
Age Group: 81-85 (ref: >= 91)	0.41 (0.37-0.47)	<0.001	0.43 (0.39-0.48)	<0.001
Age Group: 75-80 (ref: >= 91)	0.25 (0.21-0.29)	<0.001	0.27 (0.24-0.3)	<0.001
Sex: W (ref: M)	0.71 (0.66-0.77)	<0.001	0.68 (0.65-0.72)	<0.001
Number Medication groups: 2-5 (ref: 0-1)	1.30 (1.18-1.44)	<0.001	1.18 (1.06-1.32)	0.0024
Number Medication groups: 6-10 (ref: 0-1)	1.68 (1.53-1.84)	<0.001	1.28 (1.09-1.5)	0.0030
Number Medication groups: >=11 (ref: 0-1)	2.43 (2.31-2.55)	<0.001	1.6 (1.26-2.03)	<0.001
Wave: 2016/2017 (ref: Wave 2015/2016)	0.93 (0.72-1.19)	0.5520	0.98 (0.77-1.25)	0.8789
Wave: 2017/2018 (ref: Wave 2015/2016)	1.00 (0.77-1.31)	0.9935	1.07 (0.84-1.36)	0.5737
Wave: 2018/2019 (ref: Wave 2015/2016)	1.08 (0.81-1.44)	0.6104	1.13 (0.88-1.46)	0.3303
Wave: 2019/2020 (ref: Wave 2015/2016)	1.05 (0.85-1.3)	0.6256	1.10 (0.90-1.34)	0.3414
Wave: 2020/2021 (ref: Wave 2015/2016)	3.59 (1.32-9.73)	0.0120	3.85 (1.02-14.5)	0.0466
Anticoagulants: Yes (ref: No)	1.08 (0.99-1.18)	0.0694	1.12 (1.04-1.21)	0.0016
Antibiotics, Antivirals, Antiprotozoals or Anthelmintics: Yes (ref: No)	1.07 (0.96-1.20)	0.2422		
Insulins and other Antidiabetics: Yes (ref: No)	1.10 (1.02-1.19)	0.0197	1.19 (1.1-1.28)	<0.001
"heart" drugs: Yes (ref: No)	1.06 (1.00-1.12)	0.0701	1.08 (1.02-1.13)	0.0043
Antihypertensives incl. Diuretics and Renin-angiotensin-aldosterone system inhibitors: Yes (ref: No)	0.90 (0.83-0.97)	0.0081	0.91 (0.83-1.00)	0.0450
Beta Blockers: Yes (ref: No)	0.97 (0.91-1.03)	0.2669		
Statins, Fibrates incl. Proprotein convertase subtilisin/kexin type 9 inhibitors and Inclisiran: Yes (ref: No)	0.78 (0.72-0.84)	<0.001	0.80 (0.75-0.86)	<0.001
Immunosuppressants and Immunomodulators: Yes (ref: No)	1.08 (0.88-1.33)	0.4605		
Systemic Steroids: Yes (ref: No)	0.99 (0.89-1.11)	0.8864		
Chemotherapy: Yes (ref: No)	1.08 (0.92-1.26)	0.3664		
Iron supplements, Erythropoietic stimulating agents, Vitamin B12, folic acid: Yes (ref: No)	1.35 (1.23-1.49)	<0.001	1.30 (1.18-1.44)	<0.001
Antacids incl. Antihistamines: Yes (ref: No)	1.16 (1.10-1.22)	<0.001	1.14 (1.09-1.19)	<0.001
Vitamin D and other Vitamin supplements: Yes (ref: No)	1.02 (0.93-1.11)	0.7019		
Caplacizumab: Yes (ref: No)	NA (NA-NA)	NA		
Systemic Hemostatics: Yes (ref: No)	2.24 (0.38-13.16)	0.3726		
Hereditary angioedema Therapeutics: Yes (ref: No)	NA (NA-NA)	NA		
Peripheral Vasodilators: Yes (ref: No)	0.66 (0.54-0.8)	<0.001	0.71 (0.61-0.84)	<0.001
Immunoglobulins: Yes (ref: No)	0.85 (0.16-4.46)	0.8440		
Interferons and CSF: Yes (ref: No)	1.32 (1.06-1.66)	0.0139	1.36 (1.1-1.68)	0.0048
NSAR and other anti-inflammatory drugs: Yes (ref: No)	0.74 (0.69-0.79)	<0.001	0.76 (0.71-0.82)	<0.001
Gout medications: Yes (ref: No)	1.10 (1.04-1.17)	0.0014	1.13 (1.06-1.22)	<0.001
Antiepileptics: Yes (ref: No)	1.04 (0.93-1.17)	0.4633		
Antipsychotics: Yes (ref: No)	1.44 (1.40-1.48)	<0.001	1.42 (1.37-1.48)	<0.001
inhaled anti-obstructive drugs: Yes (ref: No)	1.13 (1.06-1.20)	<0.001	1.16 (1.07-1.25)	<0.001
inhaled steroids: Yes (ref: No)	0.81 (0.63-1.05)	0.1187		
other COPD drugs: Yes (ref: No)	0.83 (0.57-1.21)	0.3352		
Systemic Antihistamines: Yes (ref: No)	1.08 (0.95-1.23)	0.2470		

Figure S2: Summary of Hazard ratios and 95%-confidence intervals for age, sex and medication group-effects within COVID-19 (red) and Influenza patients (blue) for the outcome all-cause death in the age groups. Age: A hazard ratio larger than one is indicating a higher risk in younger age groups. Sex: A hazard ratio larger than one is indicating a higher risk in female patients. Medication Groups: a hazard ratio larger than one is indicating a higher risk in patients with a larger number of prescribed medication groups. Significant comparisons (with p<0.05) are marked with a blue star.

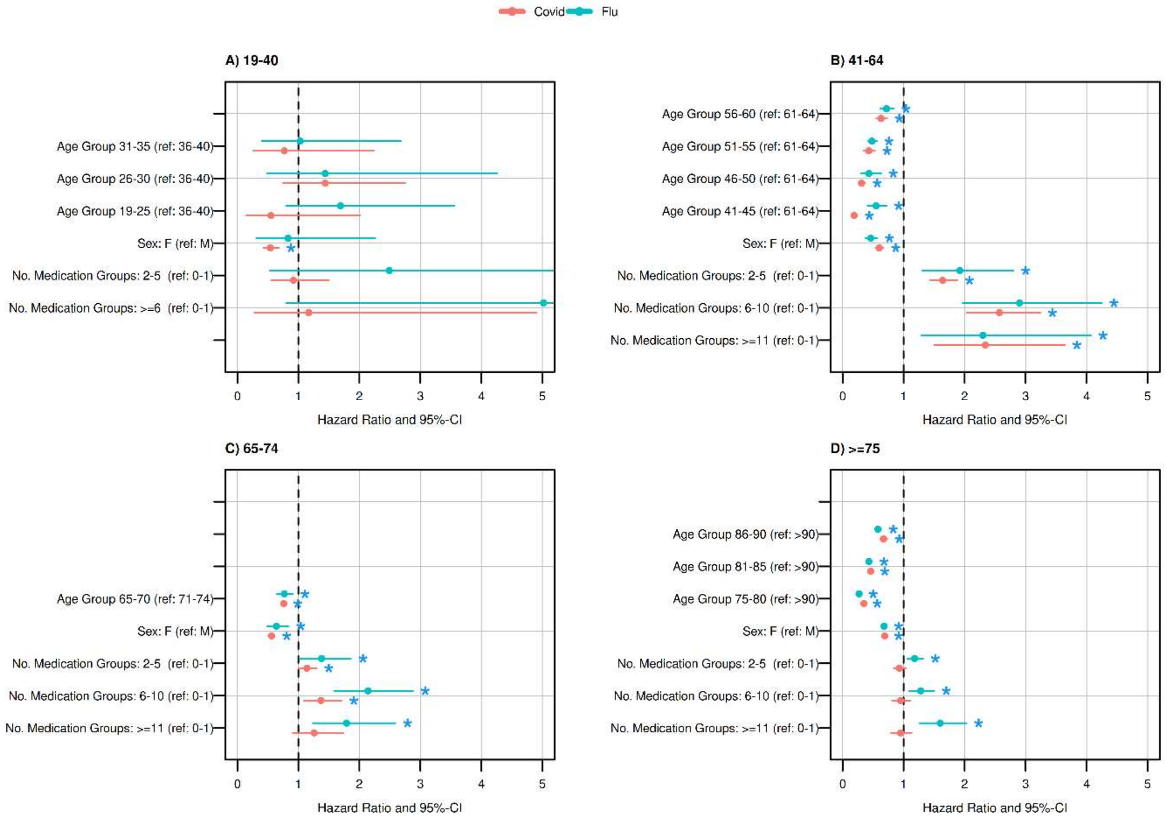

In-Hospital Mortality

Table S28: Results of the simple and multivariable Cox regression models for in-hospital mortality comparing propensity score matched COVID-19 and Influenza patients of age 19 to 40.

	Simple		Multivariable	
**Confounder**	**HR (CI)**	**p- value**	**HR (CI)**	**p- value**
**Group: Flu (ref: Covid)**	1.75 (0.50-6.05)	0.3802	1.47 (0.54-4.00)	0.4476
**Age Group: 31-35 (ref: 36-40)**	0.31 (0.06-1.77)	0.1899	0.43 (0.09-2.13)	0.2984
**Age Group: 26-30 (ref: 36-40)**	3.02 (1.33-6.87)	0.0085	2.94 (1.08-7.96)	0.0343
**Age Group: 19-25 (ref: 36-40)**	2.19 (0.51-9.51)	0.2936	2.18 (0.58-8.25)	0.2507
**Sex: W (ref: M)**	0.42 (0.03-5.55)	0.5138	0.39 (0.03-5.43)	0.4809
**Number Medication groups: 2-5 (ref: 0-1)**	0.82 (0.19-3.6)	0.7906	1.31 (0.26-6.64)	0.7457
**Number Medication groups: >=6 (ref: 0-1)**	NA (NA-NA)	NA	NA (NA-NA)	NA
**Anticoagulants: Yes (ref: No)**	3.40 (0.70-16.53)	0.1294		
**Antibiotics, Antivirals, Antiprotozoals or Anthelmintics: Yes (ref: No)**	0.83 (0.25-2.83)	0.7702		
**Insulins and other Antidiabetics: Yes (ref: No)**	3.19 (0.80-12.71)	0.1007		
**"heart" drugs: Yes (ref: No)**	NA (NA-NA)	NA		
**Antihypertensives incl. Diuretics and Renin-angiotensin-aldosterone system inhibitors: Yes (ref: No)**	0.57 (0.18-1.74)	0.3218		
**Beta Blockers: Yes (ref: No)**	4.09 (2.63-6.35)	<0.001	3.97 (2.66-5.91)	<0.001
**Statins, Fibrates incl. Proprotein convertase subtilisin/kexin type 9 inhibitors and Inclisiran: Yes (ref: No)**	NA (NA-NA)	NA		
**Immunosuppressants and Immunomodulators: Yes (ref: No)**	NA (NA-NA)	NA		
**Systemic Steroids: Yes (ref: No)**	0.78 (0.27-2.25)	0.6476		
**Chemotherapy: Yes (ref: No)**	NA (NA-NA)	NA		
**Iron supplements, Erythropoietic stimulating agents, Vitamin B12, folic acid: Yes (ref: No)**	2.31 (0.53-10.11)	0.2681		
**Antacids incl. Antihistamines: Yes (ref: No)**	0.29 (0.06-1.47)	0.1344		
**Vitamin D and other Vitamin supplements: Yes (ref: No)**	2.66 (0.05-136.67)	0.6267		
**Caplacizumab: Yes (ref: No)**	NA (NA-NA)	NA		
**Systemic Hemostatics: Yes (ref: No)**	NA (NA-NA)	NA		
**Hereditary angioedema Therapeutics: Yes (ref: No)**	NA (NA-NA)	NA		
**Peripheral Vasodilators: Yes (ref: No)**	NA (NA-NA)	NA		
**Immunoglobulins: Yes (ref: No)**	NA (NA-NA)	NA		
**Interferons and CSF: Yes (ref: No)**	NA (NA-NA)	NA		
**NSAR and other anti-inflammatory drugs: Yes (ref: No)**	0.34 (0.16-0.72)	0.0050	0.34 (0.18-0.63)	<0.001
**Gout medications: Yes (ref: No)**	NA (NA-NA)	NA		
**Antiepileptics: Yes (ref: No)**	1.23 (0.52-2.91)	0.6397		
**Antipsychotics: Yes (ref: No)**	0.53 (0.34-0.83)	0.0052	0.30 (0.12-0.77)	0.0118
**inhaled anti-obstructive drugs: Yes (ref: No)**	NA (NA-NA)	NA		
**inhaled steroids: Yes (ref: No)**	1.04 (0.36-3.02)	0.9468		
**other COPD drugs: Yes (ref: No)**	NA (NA-NA)	NA		
**Systemic Antihistamines: Yes (ref: No)**	1.06 (0.54-2.05)	0.8733		

Table S29: Results of the simple and multivariable Cox regression models for in-hospital mortality comparing propensity score matched COVID-19 and Influenza patients of age 41 to 64.

	Simple		Multivariable	
**Confounder**	**HR (CI)**	**p- value**	**HR (CI)**	**p- value**
**Group: Flu (ref: Covid)**	0.76 (0.59-0.97)	0.0265	0.71 (0.52-0.96)	0.0287
**Age Group: 56-60 (ref: 61-64)**	0.85 (0.64-1.14)	0.2735	0.83 (0.63-1.09)	0.1841
**Age Group: 51-55 (ref: 61-64)**	0.53 (0.38-0.73)	<0.001	0.51 (0.37-0.71)	<0.001
**Age Group: 46-50 (ref: 61-64)**	0.45 (0.21-0.94)	0.0344	0.44 (0.21-0.9)	0.0247
**Age Group: 41-55 (ref: 61-64)**	0.38 (0.16-0.86)	0.0210	0.37 (0.17-0.82)	0.0138
**Sex: W (ref: M)**	0.87 (0.62-1.22)	0.4082	0.83 (0.58-1.17)	0.2754
**Number Medication groups: 2-5 (ref: 0-1)**	1.47 (1.17-1.85)	<0.001	1.40 (1.14-1.72)	0.0012
**Number Medication groups: 6-10 (ref: 0-1)**	2.82 (2.42-3.29)	<0.001	2.23 (1.71-2.9)	<0.001
**Number Medication groups: >=11 (ref: 0-1)**	2.30 (0.94-5.63)	0.0671	1.52 (0.51-4.54)	0.4540
**Anticoagulants: Yes (ref: No)**	1.25 (1.07-1.46)	0.0051	1.24 (1.04-1.48)	0.0157
**Antibiotics, Antivirals, Antiprotozoals or Anthelmintics: Yes (ref: No)**	1.09 (0.91-1.3)	0.3623		
**Insulins and other Antidiabetics: Yes (ref: No)**	1.01 (0.75-1.36)	0.9596		
**"heart" drugs: Yes (ref: No)**	0.89 (0.62-1.27)	0.5110		
**Antihypertensives incl. Diuretics and Renin-angiotensin-aldosterone system inhibitors: Yes (ref: No)**	1.11 (0.94-1.3)	0.2176		
**Beta Blockers: Yes (ref: No)**	1.07 (0.81-1.41)	0.6240		
**Statins, Fibrates incl. Proprotein convertase subtilisin/kexin type 9 inhibitors and Inclisiran: Yes (ref: No)**	0.89 (0.67-1.18)	0.4104		
**Immunosuppressants and Immunomodulators: Yes (ref: No)**	0.77 (0.58-1.02)	0.0694	0.77 (0.56-1.06)	0.1040
**Systemic Steroids: Yes (ref: No)**	0.95 (0.77-1.19)	0.6732		
**Chemotherapy: Yes (ref: No)**	1.53 (0.82-2.84)	0.1812		
**Iron supplements, Erythropoietic stimulating agents, Vitamin B12, folic acid: Yes (ref: No)**	1.35 (0.73-2.53)	0.3418		
**Antacids incl. Antihistamines: Yes (ref: No)**	0.90 (0.73-1.11)	0.3324		
**Vitamin D and other Vitamin supplements: Yes (ref: No)**	1.7 (1.17-2.46)	0.0053	1.70 (1.2-2.4)	0.0027
**Caplacizumab: Yes (ref: No)**	NA (NA-NA)	NA		
**Systemic Hemostatics: Yes (ref: No)**	NA (NA-NA)	NA		
**Hereditary angioedema Therapeutics: Yes (ref: No)**	NA (NA-NA)	NA		
**Peripheral Vasodilators: Yes (ref: No)**	0.69 (0.34-1.41)	0.3116		
**Immunoglobulins: Yes (ref: No)**	NA (NA-NA)	NA		
**Interferons and CSF: Yes (ref: No)**	1.01 (0.43-2.35)	0.9906		
**NSAR and other anti-inflammatory drugs: Yes (ref: No)**	0.77 (0.64-0.93)	0.0054	0.77 (0.65-0.92)	0.0045
**Gout medications: Yes (ref: No)**	1.16 (0.81-1.65)	0.4170		
**Antiepileptics: Yes (ref: No)**	1.2 (0.92-1.56)	0.1862		
**Antipsychotics: Yes (ref: No)**	1.23 (0.97-1.56)	0.0887	1.15 (0.91-1.44)	0.2429
**inhaled anti-obstructive drugs: Yes (ref: No)**	0.86 (0.64-1.15)	0.3060		
**inhaled steroids: Yes (ref: No)**	0.64 (0.31-1.32)	0.2291		
**other COPD drugs: Yes (ref: No)**	0.83 (0.38-1.82)	0.6463		
**Systemic Antihistamines: Yes (ref: No)**	1.07 (0.77-1.48)	0.6759		

Table S30: Results of the simple and multivariable Cox regression models for in-hospital mortality comparing propensity score matched COVID-19 and Influenza patients of age 65 to 74.

	Simple		Multivariable	
**Confounder**	**HR (CI)**	**p- value**	**HR (CI)**	**p- value**
**Group: Flu (ref: Covid)**	0.39 (0.28-0.53)	<0.001	0.38 (0.28-0.52)	<0.001
**Age Group: 65-70 (ref: 71-74)**	0.83 (0.75-0.92)	<0.001	0.85 (0.76-0.96)	0.0084
**Sex: W (ref: M)**	0.58 (0.5-0.67)	<0.001	0.59 (0.5-0.69)	<0.001
**Number Medication groups: 2-5 (ref: 0-1)**	1.20 (0.91-1.58)	0.2041	1.29 (0.95-1.75)	0.1006
**Number Medication groups: 6-10 (ref: 0-1)**	1.66 (1.29-2.13)	<0.001	1.85 (1.28-2.66)	0.0010
**Number Medication groups: >=11 (ref: 0-1)**	1.82 (1.31-2.53)	<0.001	2.05 (1.19-3.53)	0.0100
**Anticoagulants: Yes (ref: No)**	1.05 (0.91-1.21)	0.5218		
**Antibiotics, Antivirals, Antiprotozoals or Anthelmintics: Yes (ref: No)**	0.90 (0.75-1.1)	0.3052		
**Insulins and other Antidiabetics: Yes (ref: No)**	1.01 (0.87-1.18)	0.8913		
**"heart" drugs: Yes (ref: No)**	1.20 (0.97-1.49)	0.0850	1.15 (0.96-1.37)	0.1202
**Antihypertensives incl. Diuretics and Renin-angiotensin-aldosterone system inhibitors: Yes (ref: No)**	0.80 (0.66-0.96)	0.0180	0.81 (0.68-0.96)	0.0177
**Beta Blockers: Yes (ref: No)**	1.13 (0.93-1.36)	0.2219		
**Statins, Fibrates incl. Proprotein convertase subtilisin/kexin type 9 inhibitors and Inclisiran: Yes (ref: No)**	0.92 (0.81-1.05)	0.2091		
**Immunosuppressants and Immunomodulators: Yes (ref: No)**	1.08 (0.68-1.73)	0.7383		
**Systemic Steroids: Yes (ref: No)**	0.87 (0.71-1.07)	0.1819		
**Chemotherapy: Yes (ref: No)**	1.15 (0.9-1.47)	0.2584		
**Iron supplements, Erythropoietic stimulating agents, Vitamin B12, folic acid: Yes (ref: No)**	1.29 (1.05-1.59)	0.0147	1.17 (0.95-1.44)	0.1301
**Antacids incl. Antihistamines: Yes (ref: No)**	1.14 (0.96-1.35)	0.1235		
**Vitamin D and other Vitamin supplements: Yes (ref: No)**	0.92 (0.70-1.20)	0.5231		
**Caplacizumab: Yes (ref: No)**	NA (NA-NA)	NA		
**Systemic Hemostatics: Yes (ref: No)**	NA (NA-NA)	NA		
**Hereditary angioedema Therapeutics: Yes (ref: No)**	NA (NA-NA)	NA		
**Peripheral Vasodilators: Yes (ref: No)**	0.76 (0.42-1.38)	0.3693		
**Immunoglobulins: Yes (ref: No)**	NA (NA-NA)	NA		
**Interferons and CSF: Yes (ref: No)**	1.00 (0.67-1.48)	0.9891		
**NSAR and other anti-inflammatory drugs: Yes (ref: No)**	0.64 (0.49-0.83)	<0.001	0.66 (0.51-0.85)	0.0013
**Gout medications: Yes (ref: No)**	1.09 (0.97-1.22)	0.1327		
**Antiepileptics: Yes (ref: No)**	1.26 (0.99-1.61)	0.0569	1.16 (0.92-1.47)	0.2154
**Antipsychotics: Yes (ref: No)**	1.35 (1.12-1.61)	0.0012	1.27 (1.05-1.53)	0.0125
**inhaled anti-obstructive drugs: Yes (ref: No)**	1.11 (0.92-1.33)	0.2826		
**inhaled steroids: Yes (ref: No)**	0.89 (0.52-1.53)	0.6757		
**other COPD drugs: Yes (ref: No)**	0.50 (0.23-1.07)	0.0749	0.58 (0.27-1.24)	0.1589
**Systemic Antihistamines: Yes (ref: No)**	0.72 (0.56-0.92)	0.0083	0.76 (0.60-0.97)	0.0254

Table S31: Results of the simple and multivariable Cox regression models for in-hospital mortality comparing propensity score matched COVID-19 and Influenza patients of age 75 and older.

	Simple		Multivariable	
**Confounder**	**HR (CI)**	**p- value**	**HR (CI)**	**p- value**
**Group: Flu (ref: Covid)**	0.39 (0.35-0.42)	<0.001	0.38 (0.35-0.42)	<0.001
**Age Group: 86-90 (ref: >= 91)**	0.68 (0.61-0.75)	<0.001	0.70 (0.63-0.79)	<0.001
**Age Group: 81-85 (ref: >= 91)**	0.51 (0.46-0.56)	<0.001	0.55 (0.5-0.6)	<0.001
**Age Group: 75-80 (ref: >= 91)**	0.39 (0.35-0.43)	<0.001	0.44 (0.39-0.49)	<0.001
**Sex: W (ref: M)**	0.66 (0.62-0.71)	<0.001	0.64 (0.6-0.69)	<0.001
**Number Medication groups: 2-5 (ref: 0-1)**	1.10 (1.02-1.19)	0.0175	0.98 (0.85-1.14)	0.8280
**Number Medication groups: 6-10 (ref: 0-1)**	1.29 (1.16-1.44)	<0.001	1.04 (0.85-1.27)	0.7177
**Number Medication groups: >=11 (ref: 0-1)**	1.42 (1.17-1.71)	<0.001	1.02 (0.77-1.35)	0.8986
**Anticoagulants: Yes (ref: No)**	1.11 (1.04-1.18)	<0.001	1.13 (1.07-1.21)	<0.001
**Antibiotics, Antivirals, Antiprotozoals or Anthelmintics: Yes (ref: No)**	1.12 (0.99-1.26)	0.0668	1.09 (0.96-1.23)	0.1655
**Insulins and other Antidiabetics: Yes (ref: No)**	0.99 (0.95-1.04)	0.7558		
**"heart" drugs: Yes (ref: No)**	1.03 (0.94-1.12)	0.5739		
**Antihypertensives incl. Diuretics and Renin-angiotensin-aldosterone system inhibitors: Yes (ref: No)**	0.93 (0.84-1.02)	0.1277		
**Beta Blockers: Yes (ref: No)**	1.07 (0.97-1.18)	0.1961		
**Statins, Fibrates incl. Proprotein convertase subtilisin/kexin type 9 inhibitors and Inclisiran: Yes (ref: No)**	0.77 (0.72-0.83)	<0.001	0.81 (0.74-0.88)	<0.001
**Immunosuppressants and Immunomodulators: Yes (ref: No)**	1.05 (0.86-1.30)	0.6268		
**Systemic Steroids: Yes (ref: No)**	1.03 (0.88-1.20)	0.7315		
**Chemotherapy: Yes (ref: No)**	0.89 (0.72-1.09)	0.2686		
**Iron supplements, Erythropoietic stimulating agents, Vitamin B12, folic acid: Yes (ref: No)**	1.24 (1.12-1.37)	<0.001	1.19 (1.11-1.29)	<0.001
**Antacids incl. Antihistamines: Yes (ref: No)**	1.07 (1.00-1.14)	0.0413	1.04 (0.98-1.10)	0.2456
**Vitamin D and other Vitamin supplements: Yes (ref: No)**	0.98 (0.87-1.11)	0.7960		
**Caplacizumab: Yes (ref: No)**	NA (NA-NA)	NA		
**Systemic Hemostatics: Yes (ref: No)**	2.19 (0.66-7.31)	0.2030		
**Hereditary angioedema Therapeutics: Yes (ref: No)**	NA (NA-NA)	NA		
**Peripheral Vasodilators: Yes (ref: No)**	0.70 (0.37-1.33)	0.2767		
**Immunoglobulins: Yes (ref: No)**	2.91 (0.68-12.39)	0.1482		
**Interferons and CSF: Yes (ref: No)**	1.12 (0.87-1.43)	0.3836		
**NSAR and other anti-inflammatory drugs: Yes (ref: No)**	0.83 (0.76-0.91)	<0.001	0.86 (0.79-0.93)	<0.001
**Gout medications: Yes (ref: No)**	1.11 (0.99-1.25)	0.0746	1.13 (1-1.28)	0.0511
**Antiepileptics: Yes (ref: No)**	0.97 (0.90-1.05)	0.4669		
**Antipsychotics: Yes (ref: No)**	1.29 (1.22-1.37)	<0.001	1.27 (1.20-1.35)	<0.001
**inhaled anti-obstructive drugs: Yes (ref: No)**	0.98 (0.89-1.08)	0.7055		
**inhaled steroids: Yes (ref: No)**	1.07 (0.84-1.36)	0.6006		
**other COPD drugs: Yes (ref: No)**	0.95 (0.62-1.43)	0.7918		
**Systemic Antihistamines: Yes (ref: No)**	1.05 (0.97-1.15)	0.2232		

Figure S3: Summary of Hazard ratios and 95%-confidence intervals for group, age, sex and medication group-effects comparing COVID-19 and Influenza patients for the outcome in-hospital mortality in the age groups. Group: A hazard ratio larger than one is indicating a higher risk of the event in the Influenza group. Age: A hazard ratio larger than one is indicating a higher risk in younger age groups. Sex: A hazard ratio larger than one is indicating a higher risk in female patients. Medication Groups: a hazard ratio larger than one is indicating a higher risk in patients with a larger number of prescribed medication groups. Significant comparisons (with p<0.05) are marked with a blue star.

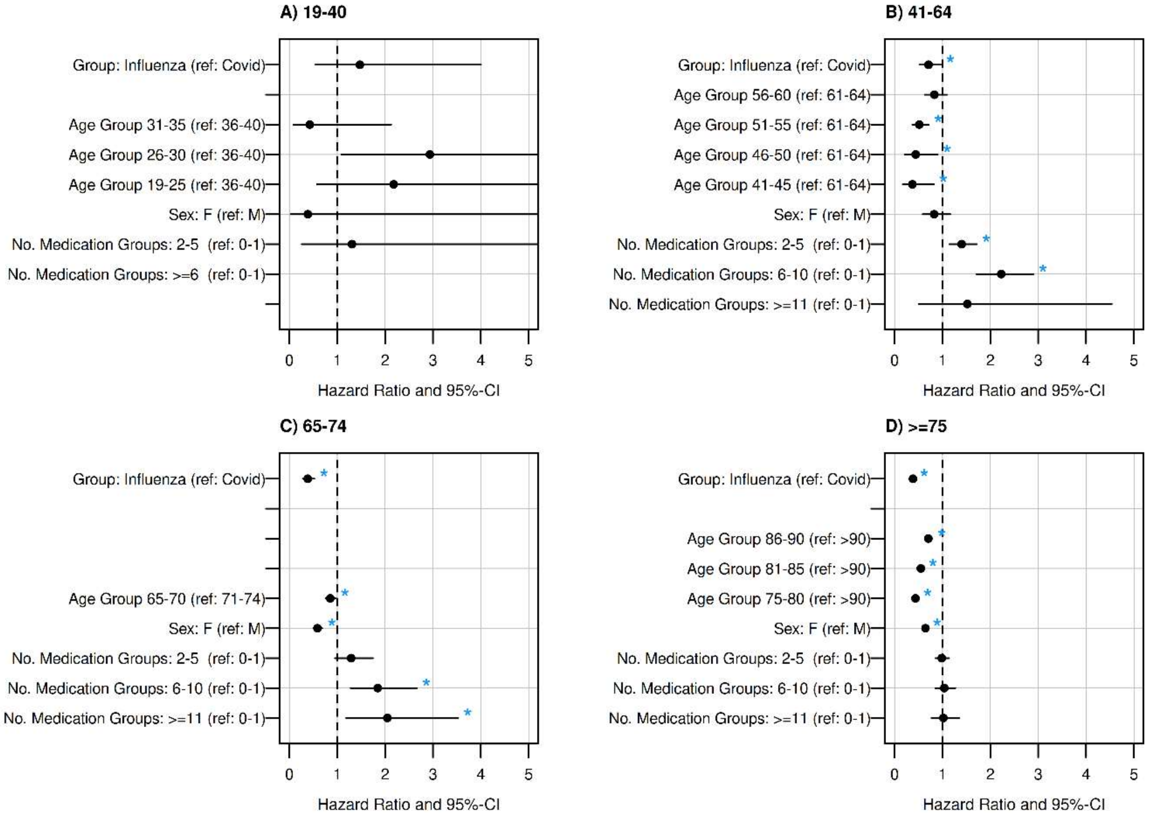

All-Cause Mortality in hospital survivors

Table S32: Results of the simple and multivariable Cox regression for all-cause mortality after hospital discharge models comparing propensity score matched COVID-19 and Influenza patients of age 19 to 40.

	Simple		Multivariable	
**Confounder**	**HR (CI)**	**p- value**	**HR (CI)**	**p- value**
**Group: Flu (ref: Covid)**	NA (NA-NA)	NA	NA (NA-NA)	NA
**Age Group: 31-35 (ref: 36-40)**	0.36 (0.09-1.48)	0.1566	0.41 (0.13-1.34)	0.1399
**Age Group: 26-30 (ref: 36-40)**	0.23 (0.05-1.03)	0.0546	0.22 (0.10-0.47)	<0.001
**Age Group: 19-25 (ref: 36-40)**	0.68 (0.27-1.67)	0.3946	0.75 (0.23-2.45)	0.6317
**Sex: W (ref: M)**	0.98 (0.41-2.33)	0.9580	0.92 (0.31-2.73)	0.8795
**Number Medication groups: 2-5 (ref: 0-1)**	2.44 (0.64-9.24)	0.1900	1.93 (0.31-11.92)	0.4776
**Number Medication groups: >=6 (ref: 0-1)**	NA (NA-NA)	NA	NA (NA-NA)	NA
**Length of hospital stay (days)**	1.05 (1.00-1.10)	0.0527	1.05 (1.00-1.10)	0.0509
**Anticoagulants: Yes (ref: No)**	2.22 (1.00-4.91)	0.0487	1.51 (0.49-4.59)	0.4722
**Antibiotics, Antivirals, Antiprotozoals or Anthelmintics: Yes (ref: No)**	1.19 (0.47-3.01)	0.7173		
**Insulins and other Antidiabetics: Yes (ref: No)**	3.02 (0.47-19.6)	0.2464		
**"heart" drugs: Yes (ref: No)**	2.94 (1.49-5.84)	0.0020	3.25 (0.97-10.9)	0.0566
**Antihypertensives incl. Diuretics and Renin-angiotensin-aldosterone system inhibitors: Yes (ref: No)**	NA (NA-NA)	NA		
**Beta Blockers: Yes (ref: No)**	1.02 (0.63-1.66)	0.9344		
**Statins, Fibrates incl. Proprotein convertase subtilisin/kexin type 9 inhibitors and Inclisiran: Yes (ref: No)**	0.62 (0.08-4.99)	0.6507		
**Immunosuppressants and Immunomodulators: Yes (ref: No)**	1.79 (0.59-5.40)	0.3024		
**Systemic Steroids: Yes (ref: No)**	0.83 (0.17-4.05)	0.8210		
**Chemotherapy: Yes (ref: No)**	NA (NA-NA)	NA		
**Iron supplements, Erythropoietic stimulating agents, Vitamin B12, folic acid: Yes (ref: No)**	5.46 (3.24-9.20)	<0.001	5.47 (2.67-11.21)	<0.001
**Antacids incl. Antihistamines: Yes (ref: No)**	2.24 (0.42-11.84)	0.3433		
**Vitamin D and other Vitamin supplements: Yes (ref: No)**	0.44 (0.19-1.04)	0.0624	0.68 (0.14-3.21)	0.6279
**Caplacizumab: Yes (ref: No)**	NA (NA-NA)	NA		
**Systemic Hemostatics: Yes (ref: No)**	NA (NA-NA)	NA		
**Hereditary angioedema Therapeutics: Yes (ref: No)**	NA (NA-NA)	NA		
**Peripheral Vasodilators: Yes (ref: No)**	NA (NA-NA)	NA		
**Immunoglobulins: Yes (ref: No)**	NA (NA-NA)	NA		
**Interferons and CSF: Yes (ref: No)**	NA (NA-NA)	NA		
**NSAR and other anti-inflammatory drugs: Yes (ref: No)**	0.50 (0.23-1.07)	0.0735	1.22 (0.45-3.26)	0.6951
**Gout medications: Yes (ref: No)**	NA (NA-NA)	NA		
**Antiepileptics: Yes (ref: No)**	1.57 (0.43-5.71)	0.4964		
**Antipsychotics: Yes (ref: No)**	0.79 (0.32-1.95)	0.6019		
**inhaled anti-obstructive drugs: Yes (ref: No)**	1.21 (0.52-2.80)	0.6540		
**inhaled steroids: Yes (ref: No)**	NA (NA-NA)	NA		
**other COPD drugs: Yes (ref: No)**	1.16 (0.20-6.65)	0.8657		
**Systemic Antihistamines: Yes (ref: No)**	NA (NA-NA)	NA		

Table S33: Results of the simple and multivariable Cox regression models for all-cause mortality after hospital discharge comparing propensity score matched COVID-19 and Influenza patients of age 41 to 64.

	Simple		Multivariable	
**Confounder**	**HR (CI)**	**p- value**	**HR (CI)**	**p- value**
**Group: Flu (ref: Covid)**	1.62 (1.13-2.31)	0.0082	1.67 (1.18-2.36)	0.0039
**Age Group: 56-60 (ref: 61-64)**	0.72 (0.62-0.84)	<0.001	0.70 (0.63-0.78)	<0.001
**Age Group: 51-55 (ref: 61-64)**	0.58 (0.41-0.81)	0.0013	0.55 (0.39-0.76)	<0.001
**Age Group: 46-50 (ref: 61-64)**	0.40 (0.26-0.61)	<0.001	0.35 (0.23-0.54)	<0.001
**Age Group: 41-55 (ref: 61-64)**	0.42 (0.32-0.54)	<0.001	0.40 (0.30-0.54)	<0.001
**Sex: W (ref: M)**	0.53 (0.42-0.65)	<0.001	0.48 (0.38-0.60)	<0.001
**Number Medication groups: 2-5 (ref: 0-1)**	2.28 (1.46-3.55)	<0.001	1.83 (1.12-2.99)	0.0162
**Number Medication groups: 6-10 (ref: 0-1)**	4.36 (2.81-6.76)	<0.001	2.51 (1.40-4.50)	0.0019
**Number Medication groups: >=11 (ref: 0-1)**	6.70 (4.57-9.83)	<0.001	2.34 (0.96-5.67)	0.0603
**Length of hospital stay (days)**	1.02 (1.01-1.02)	<0.001	1.01 (1.01-1.02)	<0.001
**Anticoagulants: Yes (ref: No)**	1.36 (1.12-1.66)	0.0023	1.47 (1.10-1.96)	0.0084
**Antibiotics, Antivirals, Antiprotozoals or Anthelmintics: Yes (ref: No)**	0.92 (0.87-0.97)	0.0040	1.05 (0.95-1.16)	0.3308
**Insulins and other Antidiabetics: Yes (ref: No)**	0.90 (0.76-1.06)	0.2171		
**"heart" drugs: Yes (ref: No)**	1.27 (1.00-1.61)	0.0462	1.46 (1.13-1.89)	0.0035
**Antihypertensives incl. Diuretics and Renin-angiotensin-aldosterone system inhibitors: Yes (ref: No)**	0.97 (0.77-1.22)	0.7999		
**Beta Blockers: Yes (ref: No)**	0.79 (0.67-0.92)	0.0030	0.9 (0.74-1.10)	0.3091
**Statins, Fibrates incl. Proprotein convertase subtilisin/kexin type 9 inhibitors and Inclisiran: Yes (ref: No)**	0.52 (0.42-0.64)	<0.001	0.66 (0.47-0.92)	0.0146
**Immunosuppressants and Immunomodulators: Yes (ref: No)**	0.99 (0.67-1.47)	0.9775		
**Systemic Steroids: Yes (ref: No)**	1.50 (1.26-1.79)	<0.001	1.60 (1.26-2.04)	<0.001
**Chemotherapy: Yes (ref: No)**	3.84 (2.55-5.79)	<0.001	3.16 (2.32-4.32)	<0.001
**Iron supplements, Erythropoietic stimulating agents, Vitamin B12, folic acid: Yes (ref: No)**	2.92 (2.07-4.11)	<0.001	2.15 (1.58-2.92)	<0.001
**Antacids incl. Antihistamines: Yes (ref: No)**	1.14 (0.84-1.54)	0.4068		
**Vitamin D and other Vitamin supplements: Yes (ref: No)**	1.47 (1.21-1.77)	<0.001	1.33 (1.10-1.60)	0.0032
**Caplacizumab: Yes (ref: No)**	NA (NA-NA)	NA		
**Systemic Hemostatics: Yes (ref: No)**	3.98 (3.07-5.17)	<0.001	4.23 (2.79-6.43)	<0.001
**Hereditary angioedema Therapeutics: Yes (ref: No)**	NA (NA-NA)	NA		
**Peripheral Vasodilators: Yes (ref: No)**	0.61 (0.22-1.72)	0.3544		
**Immunoglobulins: Yes (ref: No)**	NA (NA-NA)	NA		
**Interferons and CSF: Yes (ref: No)**	3.39 (2.23-5.14)	<0.001	2.66 (1.73-4.09)	<0.001
**NSAR and other anti-inflammatory drugs: Yes (ref: No)**	0.49 (0.41-0.58)	<0.001	0.54 (0.44-0.66)	<0.001
**Gout medications: Yes (ref: No)**	1.26 (0.73-2.17)	0.4037		
**Antiepileptics: Yes (ref: No)**	2.35 (1.90-2.90)	<0.001	2.05 (1.63-2.57)	<0.001
**Antipsychotics: Yes (ref: No)**	1.54 (1.35-1.75)	<0.001	1.40 (1.19-1.65)	<0.001
**inhaled anti-obstructive drugs: Yes (ref: No)**	0.68 (0.46-1.01)	0.0579	0.84 (0.58-1.21)	0.3409
**inhaled steroids: Yes (ref: No)**	0.46 (0.31-0.68)	<0.001	0.63 (0.44-0.91)	0.0127
**other COPD drugs: Yes (ref: No)**	0.58 (0.39-0.89)	0.0118	0.90 (0.57-1.42)	0.6495
**Systemic Antihistamines: Yes (ref: No)**	1.38 (0.94-2.04)	0.1001		

Table S34: Results of the simple and multivariable Cox regression models for all-cause mortality after hospital discharge comparing propensity score matched COVID-19 and Influenza patients of age 65 to 74.

	Simple		Multivariable	
**Confounder**	**HR (CI)**	**p- value**	**HR (CI)**	**p- value**
**Group: Flu (ref: Covid)**	1.19 (0.87-1.64)	0.2739	1.15 (0.84-1.58)	0.3819
**Age Group: 65-70 (ref: 71-74)**	0.72 (0.65-0.80)	<0.001	0.72 (0.65-0.80)	<0.001
**Sex: W (ref: M)**	0.68 (0.58-0.79)	<0.001	0.67 (0.56-0.80)	<0.001
**Number Medication groups: 2-5 (ref: 0-1)**	1.55 (1.11-2.17)	0.0105	1.23 (0.84-1.80)	0.2918
**Number Medication groups: 6-10 (ref: 0-1)**	2.70 (1.99-3.66)	<0.001	1.48 (0.92-2.37)	0.1031
**Number Medication groups: >=11 (ref: 0-1)**	2.83 (2.00-4.00)	<0.001	1.03 (0.66-1.62)	0.8961
**Length of hospital stay (days)**	1.02 (1.01-1.02)	<0.001	1.01 (1.01-1.02)	<0.001
**Anticoagulants: Yes (ref: No)**	1.3 (1.02-1.65)	0.0368	1.38 (1.11-1.72)	0.0037
**Antibiotics, Antivirals, Antiprotozoals or Anthelmintics: Yes (ref: No)**	1.12 (0.93-1.35)	0.2252		
**Insulins and other Antidiabetics: Yes (ref: No)**	1.19 (1.07-1.33)	0.0017	1.36 (1.2-1.54)	<0.001
**"heart" drugs: Yes (ref: No)**	1.07 (0.83-1.40)	0.5939		
**Antihypertensives incl. Diuretics and Renin-angiotensin-aldosterone system inhibitors: Yes (ref: No)**	0.84 (0.68-1.05)	0.1234		
**Beta Blockers: Yes (ref: No)**	0.96 (0.77-1.19)	0.6974		
**Statins, Fibrates incl. Proprotein convertase subtilisin/kexin type 9 inhibitors and Inclisiran: Yes (ref: No)**	0.72 (0.64-0.80)	<0.001	0.77 (0.69-0.86)	<0.001
**Immunosuppressants and Immunomodulators: Yes (ref: No)**	0.90 (0.66-1.23)	0.5096		
**Systemic Steroids: Yes (ref: No)**	1.23 (1.03-1.47)	0.0239	1.31 (1.10-1.56)	0.0022
**Chemotherapy: Yes (ref: No)**	2.52 (2.04-3.12)	<0.001	2.22 (1.72-2.88)	<0.001
**Iron supplements, Erythropoietic stimulating agents, Vitamin B12, folic acid: Yes (ref: No)**	1.63 (1.30-2.05)	<0.001	1.48 (1.24-1.76)	<0.001
**Antacids incl. Antihistamines: Yes (ref: No)**	1.10 (0.96-1.27)	0.1675		
**Vitamin D and other Vitamin supplements: Yes (ref: No)**	1.02 (0.71-1.48)	0.8974		
**Caplacizumab: Yes (ref: No)**	NA (NA-NA)	NA		
**Systemic Hemostatics: Yes (ref: No)**	NA (NA-NA)	NA		
**Hereditary angioedema Therapeutics: Yes (ref: No)**	NA (NA-NA)	NA		
**Peripheral Vasodilators: Yes (ref: No)**	0.91 (0.40-2.08)	0.8234		
**Immunoglobulins: Yes (ref: No)**	NA (NA-NA)	NA		
**Interferons and CSF: Yes (ref: No)**	3.31 (2.66-4.12)	<0.001	3.01 (2.35-3.86)	<0.001
**NSAR and other anti-inflammatory drugs: Yes (ref: No)**	0.81 (0.72-0.91)	<0.001	0.88 (0.77-1.01)	0.0603
**Gout medications: Yes (ref: No)**	1.03 (0.73-1.43)	0.8770		
**Antiepileptics: Yes (ref: No)**	1.45 (1.27-1.65)	<0.001	1.41 (1.24-1.61)	<0.001
**Antipsychotics: Yes (ref: No)**	1.38 (1.19-1.60)	<0.001	1.41 (1.17-1.69)	<0.001
**inhaled anti-obstructive drugs: Yes (ref: No)**	1.06 (0.91-1.25)	0.4483		
**inhaled steroids: Yes (ref: No)**	0.69 (0.52-0.92)	0.0100	0.85 (0.65-1.11)	0.2250
**other COPD drugs: Yes (ref: No)**	1.12 (0.81-1.55)	0.4908		
**Systemic Antihistamines: Yes (ref: No)**	0.73 (0.55-0.97)	0.0315	0.78 (0.62-0.99)	0.0393

Table S35: Results of the simple and multivariable Cox regression models for all-cause mortality after hospital discharge comparing propensity score matched COVID-19 and Influenza patients of age 75 and older.

	Simple		Multivariable	
**Confounder**	**HR (CI)**	**p- value**	**HR (CI)**	**p- value**
**Group: Flu (ref: Covid)**	1.01 (0.91-1.13)	0.8439	1.01 (0.91-1.13)	0.8350
**Age Group: 86-90 (ref: >= 91)**	0.57 (0.53-0.62)	<0.001	0.59 (0.55-0.63)	<0.001
**Age Group: 81-85 (ref: >= 91)**	0.37 (0.34-0.41)	<0.001	0.39 (0.36-0.42)	<0.001
**Age Group: 75-80 (ref: >= 91)**	0.23 (0.21-0.26)	<0.001	0.26 (0.23-0.29)	<0.001
**Sex: W (ref: M)**	0.77 (0.72-0.83)	<0.001	0.74 (0.69-0.80)	<0.001
**Number Medication groups: 2-5 (ref: 0-1)**	1.21 (1.08-1.35)	<0.001	0.95 (0.80-1.13)	0.5809
**Number Medication groups: 6-10 (ref: 0-1)**	1.60 (1.40-1.83)	<0.001	0.97 (0.75-1.24)	0.7930
**Number Medication groups: >=11 (ref: 0-1)**	2.07 (1.88-2.27)	<0.001	0.98 (0.74-1.3)	0.8855
**Length of hospital stay (days)**	1.02 (1.01-1.02)	<0.001	1.02 (1.01-1.02)	<0.001
**Anticoagulants: Yes (ref: No)**	1.16 (1.06-1.26)	0.0012	1.22 (1.12-1.33)	<0.001
**Antibiotics, Antivirals, Antiprotozoals or Anthelmintics: Yes (ref: No)**	1.17 (1.04-1.30)	0.0062	1.19 (1.07-1.33)	0.0017
**Insulins and other Antidiabetics: Yes (ref: No)**	1.08 (1.01-1.15)	0.0215	1.17 (1.12-1.23)	<0.001
**"heart" drugs: Yes (ref: No)**	1.03 (0.99-1.09)	0.1758		
**Antihypertensives incl. Diuretics and Renin-angiotensin-aldosterone system inhibitors: Yes (ref: No)**	0.88 (0.81-0.96)	0.0025	0.94 (0.87-1.03)	0.1713
**Beta Blockers: Yes (ref: No)**	0.97 (0.91-1.04)	0.4547		
**Statins, Fibrates incl. Proprotein convertase subtilisin/kexin type 9 inhibitors and Inclisiran: Yes (ref: No)**	0.83 (0.78-0.89)	<0.001	0.88 (0.83-0.94)	<0.001
**Immunosuppressants and Immunomodulators: Yes (ref: No)**	1.00 (0.79-1.26)	0.9861		
**Systemic Steroids: Yes (ref: No)**	1.02 (0.95-1.09)	0.6482		
**Chemotherapy: Yes (ref: No)**	1.22 (1.08-1.38)	0.0014	1.30 (1.11-1.51)	0.0011
**Iron supplements, Erythropoietic stimulating agents, Vitamin B12, folic acid: Yes (ref: No)**	1.52 (1.39-1.67)	<0.001	1.50 (1.37-1.65)	<0.001
**Antacids incl. Antihistamines: Yes (ref: No)**	1.05 (0.97-1.13)	0.2047		
**Vitamin D and other Vitamin supplements: Yes (ref: No)**	1.02 (0.95-1.10)	0.5018		
**Caplacizumab: Yes (ref: No)**	NA (NA-NA)	NA		
**Systemic Hemostatics: Yes (ref: No)**	1.75 (0.27-11.57)	0.5607		
**Hereditary angioedema Therapeutics: Yes (ref: No)**	NA (NA-NA)	NA		
**Peripheral Vasodilators: Yes (ref: No)**	0.54 (0.40-0.73)	<0.001	0.59 (0.45-0.76)	<0.001
**Immunoglobulins: Yes (ref: No)**	0.94 (0.20-4.43)	0.9344		
**Interferons and CSF: Yes (ref: No)**	1.51 (1.10-2.06)	0.0097	1.61 (1.17-2.23)	0.0037
**NSAR and other anti-inflammatory drugs: Yes (ref: No)**	0.72 (0.68-0.76)	<0.001	0.77 (0.71-0.83)	<0.001
**Gout medications: Yes (ref: No)**	0.99 (0.92-1.07)	0.8167		
**Antiepileptics: Yes (ref: No)**	1.04 (0.97-1.12)	0.2733		
**Antipsychotics: Yes (ref: No)**	1.50 (1.43-1.57)	<0.001	1.52 (1.44-1.61)	<0.001
**inhaled anti-obstructive drugs: Yes (ref: No)**	1.13 (1.07-1.20)	<0.001	1.20 (1.11-1.30)	<0.001
**inhaled steroids: Yes (ref: No)**	0.81 (0.69-0.94)	0.0046	0.81 (0.69-0.95)	0.0097
**other COPD drugs: Yes (ref: No)**	0.71 (0.54-0.92)	0.0094	0.75 (0.59-0.97)	0.0257
**Systemic Antihistamines: Yes (ref: No)**	0.97 (0.91-1.04)	0.4035		

Figure S4: Summary of Hazard ratios and 95%-confidence intervals for group, age, sex and medication group-effects comparing COVID-19 and Influenza patients for the outcome all-cause death in hospital survivors in the age groups. Group: A hazard ratio larger than one is indicating a higher risk of the event in the Influenza group. Age: A hazard ratio larger than one is indicating a higher risk in younger age groups. Sex: A hazard ratio larger than one is indicating a higher risk in female patients. Medication Groups: a hazard ratio larger than one is indicating a higher risk in patients with a larger number of prescribed medication groups. Significant comparisons (with p<0.05) are marked with a blue star.

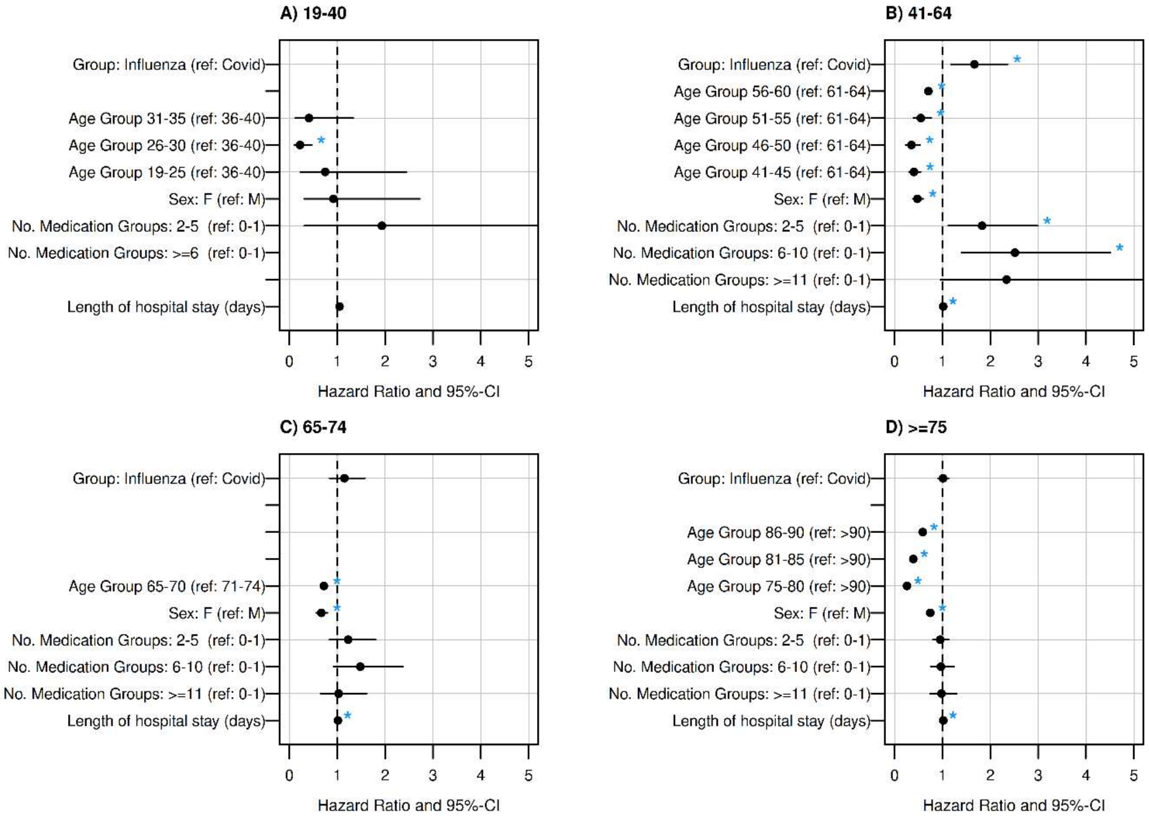

Readmission

Table S36: Results of the simple and multivariable Competing risk regression observing readmission to hospital with competing event all-cause mortality comparing propensity score matched COVID-19 and Influenza patients of age 19 to 40.

	Simple		Multivariable	
**Confounder**	**HR (CI)**	**p- value**	**HR (CI)**	**p- value**
**Group: Flu (ref: Covid)**	2.35 (1.97-2.80)	<0.001	2.35 (1.96-2.81)	<0.001
**Age Group: 31-35 (ref: 36-40)**	1.50 (1.18-1.92)	0.0011	1.54 (1.19-1.99)	<0.001
**Age Group: 26-30 (ref: 36-40)**	1.63 (1.27-2.09)	<0.001	1.74 (1.34-2.26)	<0.001
**Age Group: 19-25 (ref: 36-40)**	1.14 (0.88-1.49)	0.3200	1.20 (0.91-1.59)	0.2000
**Sex: W (ref: M)**	2.33 (1.92-2.83)	<0.001	2.35 (1.92-2.87)	<0.001
**Number Medication groups: 2-5 (ref: 0-1)**	1.08 (0.89-1.31)	0.4500	1.09 (0.82-1.45)	0.5700
**Number Medication groups: >=6 (ref: 0-1)**	2.02 (1.37-2.97)	<0.001	1.04 (0.45-2.40)	0.9300
**Length of hospital stay (days)**	1.02 (1.01-1.03)	<0.001	1.02 (1.01-1.02)	<0.001
**Anticoagulants: Yes (ref: No)**	1.75 (1.23-2.47)	0.0017	1.56 (1.06-2.31)	0.0240
**Antibiotics, Antivirals, Antiprotozoals or Anthelmintics: Yes (ref: No)**	0.90 (0.74-1.10)	0.3100		
**Insulins and other Antidiabetics: Yes (ref: No)**	1.67 (0.83-3.33)	0.1500		
**"heart" drugs: Yes (ref: No)**	0.72 (0.28-1.80)	0.4800		
**Antihypertensives incl. Diuretics and Renin-angiotensin-aldosterone system inhibitors: Yes (ref: No)**	1.51 (0.96-2.36)	0.0720	1.33 (0.78-2.28)	0.2900
**Beta Blockers: Yes (ref: No)**	1.78 (1.01-3.14)	0.0460	1.09 (0.51-2.33)	0.8300
**Statins, Fibrates incl. Proprotein convertase subtilisin/kexin type 9 inhibitors and Inclisiran: Yes (ref: No)**	0.57 (0.23-1.38)	0.2100		
**Immunosuppressants and Immunomodulators: Yes (ref: No)**	1.07 (0.53-2.16)	0.8500		
**Systemic Steroids: Yes (ref: No)**	1.15 (0.77-1.74)	0.4900		
**Chemotherapy: Yes (ref: No)**	3.06 (1.26-7.4)	0.0130	1.85 (0.58-5.94)	0.3000
**Iron supplements, Erythropoietic stimulating agents, Vitamin B12, folic acid: Yes (ref: No)**	1.79 (1.29-2.47)	<0.001	1.62 (1.14-2.3)	0.0073
**Antacids incl. Antihistamines: Yes (ref: No)**	1.61 (1.10-2.37)	0.0150	1.60 (1.08-2.37)	0.0180
**Vitamin D and other Vitamin supplements: Yes (ref: No)**	0.97 (0.61-1.53)	0.8900		
**Caplacizumab: Yes (ref: No)**	NA (NA-NA)	NA		
**Systemic Hemostatics: Yes (ref: No)**	NA (NA-NA)	NA		
**Hereditary angioedema Therapeutics: Yes (ref: No)**	NA (NA-NA)	NA		
**Peripheral Vasodilators: Yes (ref: No)**	NA (NA-NA)	NA		
**Immunoglobulins: Yes (ref: No)**	NA (NA-NA)	NA		
**Interferons and CSF: Yes (ref: No)**	1.29 (0.57-2.91)	0.5400		
**NSAR and other anti-inflammatory drugs: Yes (ref: No)**	0.62 (0.46-0.82)	0.0010	0.64 (0.47-0.87)	0.0041
**Gout medications: Yes (ref: No)**	5.48 (3.40-8.83)	<0.001	1.48 (0.29-7.50)	0.6400
**Antiepileptics: Yes (ref: No)**	1.55 (0.95-2.53)	0.0760	1.20 (0.71-2.02)	0.5000
**Antipsychotics: Yes (ref: No)**	1.43 (1.05-1.96)	0.0240	1.45 (1.05-2.01)	0.0240
**inhaled anti-obstructive drugs: Yes (ref: No)**	0.67 (0.48-0.94)	0.0210	0.71 (0.51-1.01)	0.0570
**inhaled steroids: Yes (ref: No)**	0.80 (0.41-1.57)	0.5200		
**other COPD drugs: Yes (ref: No)**	1.46 (0.46-4.61)	0.5200		
**Systemic Antihistamines: Yes (ref: No)**	0.55 (0.30-1.02)	0.0590	0.68 (0.36-1.27)	0.2300

Table S37: Results of the simple and multivariable Competing risk regression observing readmission to hospital with competing event all-cause mortality comparing propensity score matched COVID-19 and Influenza patients of age 41 to 64.

	Simple		Multivariable	
**Confounder**	**HR (CI)**	**p- value**	**HR (CI)**	**p- value**
**Group: Flu (ref: Covid)**	1.84 (1.67-2.02)	<0.001	1.83 (1.66-2.01)	<0.001
**Age Group: 56-60 (ref: 61-64)**	0.96 (0.85-1.08)	0.4700	0.94 (0.84-1.07)	0.3500
**Age Group: 51-55 (ref: 61-64)**	0.88 (0.77-1.01)	0.0720	0.88 (0.77-1.01)	0.0650
**Age Group: 46-50 (ref: 61-64)**	0.84 (0.72-0.98)	0.0250	0.80 (0.69-0.93)	0.0039
**Age Group: 41-55 (ref: 61-64)**	0.71 (0.59-0.86)	<0.001	0.69 (0.57-0.84)	<0.001
**Sex: W (ref: M)**	0.82 (0.75-0.9)	<0.001	0.81 (0.74-0.89)	<0.001
**Number Medication groups: 2-5 (ref: 0-1)**	1.59 (1.40-1.80)	<0.001	1.40 (1.21-1.62)	<0.001
**Number Medication groups: 6-10 (ref: 0-1)**	2.45 (2.14-2.81)	<0.001	1.81 (1.45-2.26)	<0.001
**Number Medication groups: >=11 (ref: 0-1)**	3.53 (2.68-4.64)	<0.001	2.01 (1.35-3.01)	<0.001
**Length of hospital stay (days)**	1.01 (1.01-1.02)	<0.001	1.01 (1.01-1.02)	<0.001
**Anticoagulants: Yes (ref: No)**	1.16 (1.03-1.31)	0.0180	1.18 (1.05-1.34)	0.0070
**Antibiotics, Antivirals, Antiprotozoals or Anthelmintics: Yes (ref: No)**	1.05 (0.95-1.16)	0.3400		
**Insulins and other Antidiabetics: Yes (ref: No)**	0.98 (0.86-1.12)	0.7800		
**"heart" drugs: Yes (ref: No)**	1.12 (0.93-1.34)	0.2400		
**Antihypertensives incl. Diuretics and Renin-angiotensin-aldosterone system inhibitors: Yes (ref: No)**	0.98 (0.88-1.09)	0.6700		
**Beta Blockers: Yes (ref: No)**	0.98 (0.86-1.11)	0.7400		
**Statins, Fibrates incl. Proprotein convertase subtilisin/kexin type 9 inhibitors and Inclisiran: Yes (ref: No)**	0.82 (0.73-0.92)	<0.001	0.90 (0.8-1.02)	0.0990
**Immunosuppressants and Immunomodulators: Yes (ref: No)**	0.97 (0.8-1.19)	0.7800		
**Systemic Steroids: Yes (ref: No)**	1.41 (1.25-1.59)	<0.001	1.43 (1.26-1.62)	<0.001
**Chemotherapy: Yes (ref: No)**	1.79 (1.37-2.34)	<0.001	1.65 (1.27-2.14)	<0.001
**Iron supplements, Erythropoietic stimulating agents, Vitamin B12, folic acid: Yes (ref: No)**	1.30 (1.10-1.55)	0.00290	1.24 (1.04-1.47)	0.0140
**Antacids incl. Antihistamines: Yes (ref: No)**	1.00 (0.89-1.14)	0.9500		
**Vitamin D and other Vitamin supplements: Yes (ref: No)**	1.05 (0.92-1.20)	0.4700		
**Caplacizumab: Yes (ref: No)**	NA (NA-NA)	NA		
**Systemic Hemostatics: Yes (ref: No)**	5.12 (4.45-5.9)	<0.001	3.99 (2.92-5.46)	<0.001
**Hereditary angioedema Therapeutics: Yes (ref: No)**	NA (NA-NA)	NA		
**Peripheral Vasodilators: Yes (ref: No)**	1.53 (0.95-2.47)	0.0810	1.42 (0.89-2.26)	0.1400
**Immunoglobulins: Yes (ref: No)**	NA (NA-NA)	NA		
**Interferons and CSF: Yes (ref: No)**	2.06 (1.61-2.66)	<0.001	1.87 (1.45-2.40)	<0.001
**NSAR and other anti-inflammatory drugs: Yes (ref: No)**	0.90 (0.82-1.00)	0.0490	0.94 (0.84-1.04)	0.2100
**Gout medications: Yes (ref: No)**	1.00 (0.78-1.30)	0.9700		
**Antiepileptics: Yes (ref: No)**	1.48 (1.29-1.69)	<0.001	1.46 (1.27-1.68)	<0.001
**Antipsychotics: Yes (ref: No)**	1.15 (1.03-1.28)	0.0098	1.11 (1.00-1.25)	0.0600
**inhaled anti-obstructive drugs: Yes (ref: No)**	0.90 (0.81-1.01)	0.0660	0.98 (0.88-1.10)	0.7900
**inhaled steroids: Yes (ref: No)**	0.68 (0.54-0.85)	<0.001	0.70 (0.56-0.88)	0.0023
**other COPD drugs: Yes (ref: No)**	1.08 (0.82-1.44)	0.5800		
**Systemic Antihistamines: Yes (ref: No)**	1.03 (0.87-1.23)	0.7200		

Table S38: Results of the simple and multivariable Competing risk regression observing readmission to hospital with competing event all-cause mortality comparing propensity score matched COVID-19 and Influenza patients of age 65 to 74.

	Simple		Multivariable	
**Confounder**	**HR (CI)**	**p- value**	**HR (CI)**	**p- value**
**Group: Flu (ref: Covid)**	1.51 (1.39-1.65)	<0.001	1.51 (1.39-1.65)	<0.001
**Age Group: 65-70 (ref: 71-74)**	0.86 (0.79-0.94)	<0.001	0.86 (0.79-0.94)	<0.001
**Sex: W (ref: M)**	0.82 (0.75-0.89)	<0.001	0.81 (0.74-0.88)	<0.001
**Number Medication groups: 2-5 (ref: 0-1)**	1.50 (1.31-1.72)	<0.001	1.31 (1.12-1.53)	<0.001
**Number Medication groups: 6-10 (ref: 0-1)**	2.11 (1.84-2.43)	<0.001	1.55 (1.26-1.90)	<0.001
**Number Medication groups: >=11 (ref: 0-1)**	2.71 (2.21-3.33)	<0.001	1.66 (1.22-2.26)	0.00130
**Length of hospital stay (days)**	1.01 (1.01-1.01)	<0.001	1.01 (1.01-1.01)	<0.001
**Anticoagulants: Yes (ref: No)**	1.16 (1.05-1.29)	0.0029	1.19 (1.08-1.32)	<0.001
**Antibiotics, Antivirals, Antiprotozoals or Anthelmintics: Yes (ref: No)**	1.13 (1.04-1.24)	0.0054	1.14 (1.04-1.25)	0.0037
**Insulins and other Antidiabetics: Yes (ref: No)**	0.95 (0.86-1.05)	0.3500		
**"heart" drugs: Yes (ref: No)**	1.11 (0.98-1.27)	0.1100		
**Antihypertensives incl. Diuretics and Renin-angiotensin-aldosterone system inhibitors: Yes (ref: No)**	0.95 (0.86-1.05)	0.3300		
**Beta Blockers: Yes (ref: No)**	1.04 (0.94-1.14)	0.4400		
**Statins, Fibrates incl. Proprotein convertase subtilisin/kexin type 9 inhibitors and Inclisiran: Yes (ref: No)**	0.93 (0.85-1.01)	0.1000	0.99 (0.90-1.09)	0.7700
**Immunosuppressants and Immunomodulators: Yes (ref: No)**	0.94 (0.78-1.15)	0.5700		
**Systemic Steroids: Yes (ref: No)**	1.22 (1.09-1.35)	<0.001	1.22 (1.09-1.36)	<0.001
**Chemotherapy: Yes (ref: No)**	1.28 (1.04-1.58)	0.0180	1.22 (0.98-1.52)	0.0690
**Iron supplements, Erythropoietic stimulating agents, Vitamin B12, folic acid: Yes (ref: No)**	1.24 (1.06-1.44)	0.0056	1.24 (1.06-1.44)	0.0063
**Antacids incl. Antihistamines: Yes (ref: No)**	0.95 (0.85-1.05)	0.3200		
**Vitamin D and other Vitamin supplements: Yes (ref: No)**	0.89 (0.79-1.00)	0.0520	0.89 (0.79-1.01)	0.0650
**Caplacizumab: Yes (ref: No)**	NA (NA-NA)	NA		
**Systemic Hemostatics: Yes (ref: No)**	2.39 (2.16-2.65)	<0.001	2.87 (2.38-3.45)	<0.001
**Hereditary angioedema Therapeutics: Yes (ref: No)**	NA (NA-NA)	NA		
**Peripheral Vasodilators: Yes (ref: No)**	0.78 (0.48-1.25)	0.3000		
**Immunoglobulins: Yes (ref: No)**	1.53 (0.26-8.88)	0.6400		
**Interferons and CSF: Yes (ref: No)**	1.76 (1.37-2.27)	<0.001	1.59 (1.22-2.08)	<0.001
**NSAR and other anti-inflammatory drugs: Yes (ref: No)**	0.92 (0.84-1.01)	0.0740	0.95 (0.86-1.04)	0.2700
**Gout medications: Yes (ref: No)**	1.02 (0.87-1.20)	0.7700		
**Antiepileptics: Yes (ref: No)**	1.07 (0.95-1.21)	0.2700		
**Antipsychotics: Yes (ref: No)**	1.15 (1.05-1.26)	0.0022	1.20 (1.09-1.31)	<0.001
**inhaled anti-obstructive drugs: Yes (ref: No)**	1.00 (0.91-1.10)	0.9900		
**inhaled steroids: Yes (ref: No)**	0.87 (0.74-1.04)	0.1300		
**other COPD drugs: Yes (ref: No)**	0.89 (0.61-1.30)	0.5500		
**Systemic Antihistamines: Yes (ref: No)**	1.03 (0.88-1.22)	0.6800		

Table S39: Results of the simple and multivariable Competing risk regression observing readmission to hospital with competing event all-cause mortality comparing propensity score matched COVID-19 and Influenza patients of age 75 and older.

	Simple		Multivariable	
**Confounder**	**HR (CI)**	**p- value**	**HR (CI)**	**p- value**
**Group: Flu (ref: Covid)**	1.47 (1.39-1.55)	<0.001	1.46 (1.39-1.54)	<0.001
**Age Group: 86-90 (ref: >= 91)**	1.14 (1.04-1.26)	0.0078	1.16 (1.05-1.28)	0.0039
**Age Group: 81-85 (ref: >= 91)**	1.13 (1.03-1.24)	0.0120	1.15 (1.04-1.26)	0.0046
**Age Group: 75-80 (ref: >= 91)**	1.11 (1.02-1.22)	0.0220	1.14 (1.04-1.26)	0.0052
**Sex: W (ref: M)**	0.86 (0.82-0.91)	<0.001	0.86 (0.82-0.91)	<0.001
**Number Medication groups: 2-5 (ref: 0-1)**	1.26 (1.15-1.38)	<0.001	1.09 (0.99-1.20)	0.0930
**Number Medication groups: 6-10 (ref: 0-1)**	1.66 (1.51-1.82)	<0.001	1.18 (1.05-1.34)	0.0080
**Number Medication groups: >=11 (ref: 0-1)**	2.17 (1.89-2.49)	<0.001	1.24 (1.02-1.51)	0.0280
**Length of hospital stay (days)**	1.01 (1.01-1.01)	<0.001	1.01 (1.01-1.01)	<0.001
**Anticoagulants: Yes (ref: No)**	1.10 (1.04-1.17)	0.0012	1.13 (1.06-1.20)	<0.001
**Antibiotics, Antivirals, Antiprotozoals or Anthelmintics: Yes (ref: No)**	1.10 (1.04-1.16)	<0.001	1.12 (1.06-1.18)	<0.001
**Insulins and other Antidiabetics: Yes (ref: No)**	1.03 (0.96-1.1)	0.4300		
**"heart" drugs: Yes (ref: No)**	1.10 (1.02-1.17)	0.0110	1.12 (1.04-1.2)	0.0022
**Antihypertensives incl. Diuretics and Renin-angiotensin-aldosterone system inhibitors: Yes (ref: No)**	1.03 (0.97-1.1)	0.3600		
**Beta Blockers: Yes (ref: No)**	1.00 (0.94-1.06)	0.9500		
**Statins, Fibrates incl. Proprotein convertase subtilisin/kexin type 9 inhibitors and Inclisiran: Yes (ref: No)**	0.96 (0.9-1.01)	0.1200		
**Immunosuppressants and Immunomodulators: Yes (ref: No)**	1.05 (0.87-1.27)	0.6300		
**Systemic Steroids: Yes (ref: No)**	1.11 (1.04-1.20)	0.0037	1.13 (1.05-1.22)	0.0013
**Chemotherapy: Yes (ref: No)**	1.10 (0.97-1.24)	0.1400		
**Iron supplements, Erythropoietic stimulating agents, Vitamin B12, folic acid: Yes (ref: No)**	1.11 (1.02-1.21)	0.0110	1.13 (1.04-1.23)	0.0033
**Antacids incl. Antihistamines: Yes (ref: No)**	1.02 (0.96-1.09)	0.5600		
**Vitamin D and other Vitamin supplements: Yes (ref: No)**	0.97 (0.91-1.04)	0.4100		
**Caplacizumab: Yes (ref: No)**	NA (NA-NA)	NA		
**Systemic Hemostatics: Yes (ref: No)**	1.13 (0.51-2.49)	0.7600		
**Hereditary angioedema Therapeutics: Yes (ref: No)**	NA (NA-NA)	NA		
**Peripheral Vasodilators: Yes (ref: No)**	0.67 (0.47-0.95)	0.0270	0.72 (0.50-1.03)	0.0710
**Immunoglobulins: Yes (ref: No)**	2.14 (0.98-4.67)	0.0570	1.94 (0.91-4.12)	0.0860
**Interferons and CSF: Yes (ref: No)**	1.34 (1.07-1.67)	0.0100	1.34 (1.07-1.67)	0.0100
**NSAR and other anti-inflammatory drugs: Yes (ref: No)**	0.96 (0.91-1.02)	0.2000		
**Gout medications: Yes (ref: No)**	1.05 (0.95-1.15)	0.3400		
**Antiepileptics: Yes (ref: No)**	1.12 (1.04-1.20)	0.0021	1.14 (1.06-1.22)	<0.001
**Antipsychotics: Yes (ref: No)**	1.06 (1.01-1.13)	0.0270	1.08 (1.02-1.15)	0.0049
**inhaled anti-obstructive drugs: Yes (ref: No)**	1.08 (1.01-1.15)	0.0190	1.11 (1.04-1.19)	0.0015
**inhaled steroids: Yes (ref: No)**	0.86 (0.75-0.99)	0.0390	0.86 (0.74-0.99)	0.0320
**other COPD drugs: Yes (ref: No)**	1.02 (0.78-1.33)	0.9000		
**Systemic Antihistamines: Yes (ref: No)**	0.93 (0.83-1.03)	0.1600		

Figure S5: Summary of Hazard ratios and 95%-confidence intervals for group, age, sex and medication group-effects comparing COVID-19 and Influenza patients for the outcome readmission in the age groups. A hazard ratio smaller than one is indicating a larger probability for the event in the reference group. Significant comparisons (with p<0.05) are marked with a blue star.

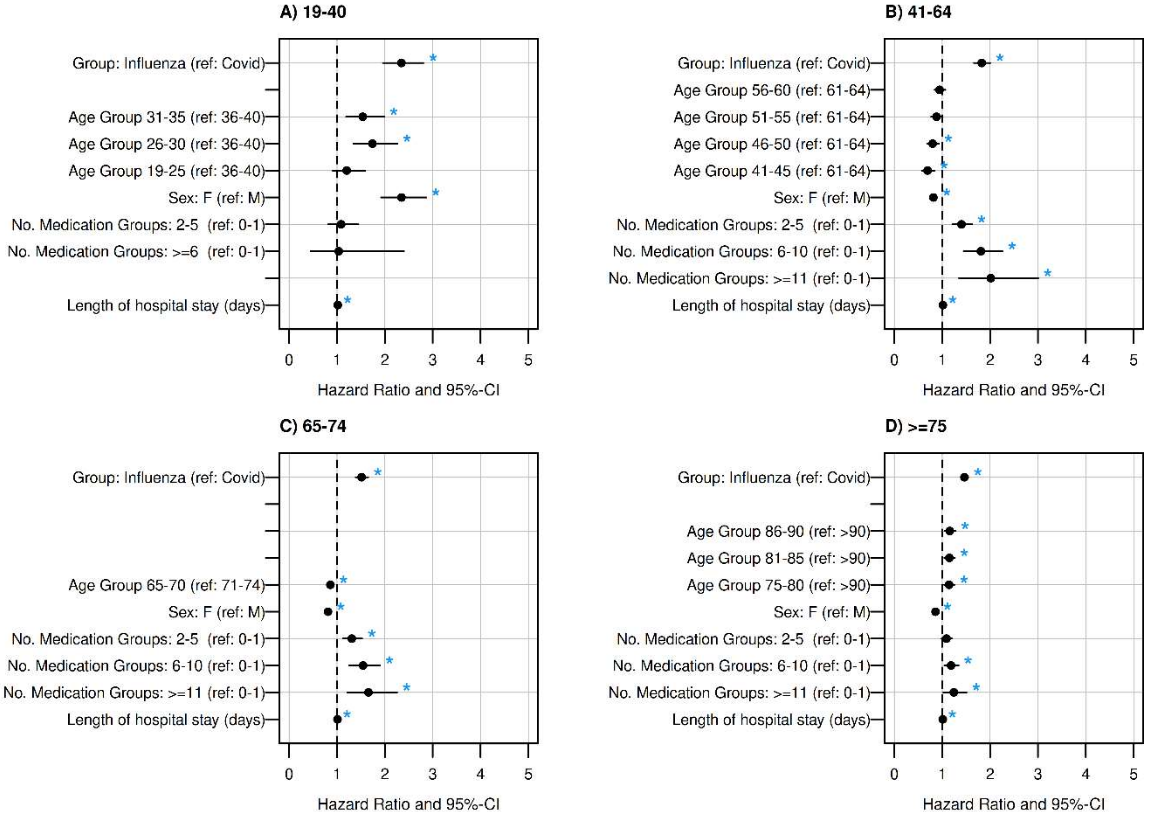

Hospital Discharge

Table S40: Results of the simple and multivariable Competing risk regression observing hospital discharge with competing event in-hospital mortality comparing propensity score matched COVID-19 and Influenza patients of age 19 to 40.

	Simple		Multivariable	
**Confounder**	**HR (CI)**	**p- value**	**HR (CI)**	**p- value**
**Group: Flu (ref: Covid)**	1.63 (1.49-1.78)	<0.001	1.62 (1.48-1.77)	<0.001
**Age Group: 31-35 (ref: 36-40)**	1.14 (1.03-1.27)	0.0120	1.16 (1.04-1.28)	0.0065
**Age Group: 26-30 (ref: 36-40)**	1.15 (1.03-1.29)	0.0150	1.13 (1.01-1.27)	0.0310
**Age Group: 19-25 (ref: 36-40)**	1.38 (1.21-1.56)	<0.001	1.36 (1.19-1.54)	<0.001
**Sex: W (ref: M)**	1.23 (1.14-1.34)	<0.001	1.23 (1.13-1.33)	<0.001
**Number Medication groups: 2-5 (ref: 0-1)**	0.82 (0.75-0.90)	<0.001	0.83 (0.74-0.92)	<0.001
**Number Medication groups: >=6 (ref: 0-1)**	0.58 (0.42-0.79)	<0.001	0.72 (0.53-0.96)	0.0280
**Anticoagulants: Yes (ref: No)**	0.86 (0.68-1.09)	0.2000		
**Antibiotics, Antivirals, Antiprotozoals or Anthelmintics: Yes (ref: No)**	1.06 (0.97-1.15)	0.2300		
**Insulins and other Antidiabetics: Yes (ref: No)**	0.65 (0.49-0.86)	0.0023	0.64 (0.48-0.85)	0.0021
**"heart" drugs: Yes (ref: No)**	0.8 (0.46-1.37)	0.4100		
**Antihypertensives incl. Diuretics and Renin-angiotensin-aldosterone system inhibitors: Yes (ref: No)**	0.73 (0.58-0.92)	0.0086	0.78 (0.63-0.97)	0.0250
**Beta Blockers: Yes (ref: No)**	0.79 (0.53-1.16)	0.2300		
**Statins, Fibrates incl. Proprotein convertase subtilisin/kexin type 9 inhibitors and Inclisiran: Yes (ref: No)**	0.82 (0.54-1.22)	0.3200		
**Immunosuppressants and Immunomodulators: Yes (ref: No)**	0.94 (0.66-1.35)	0.7600		
**Systemic Steroids: Yes (ref: No)**	0.86 (0.68-1.08)	0.1900		
**Chemotherapy: Yes (ref: No)**	1.16 (1.03-1.31)	0.0160	1.01 (0.87-1.17)	0.9200
**Iron supplements, Erythropoietic stimulating agents, Vitamin B12, folic acid: Yes (ref: No)**	1.09 (0.89-1.34)	0.4000		
**Antacids incl. Antihistamines: Yes (ref: No)**	0.96 (0.78-1.18)	0.7100		
**Vitamin D and other Vitamin supplements: Yes (ref: No)**	0.92 (0.73-1.17)	0.5000		
**Caplacizumab: Yes (ref: No)**	NA (NA-NA)	NA		
**Systemic Hemostatics: Yes (ref: No)**	0.95 (0.69-1.32)	0.7600		
**Hereditary angioedema Therapeutics: Yes (ref: No)**	1.26 (1.12-1.42)	<0.001	1.09 (0.94-1.27)	0.2300
**Peripheral Vasodilators: Yes (ref: No)**	5.65 (4.96-6.43)	<0.001	5.64 (4.95-6.42)	<0.001
**Immunoglobulins: Yes (ref: No)**	NA (NA-NA)	NA		
**Interferons and CSF: Yes (ref: No)**	1.01 (0.64-1.60)	0.9700		
**NSAR and other anti-inflammatory drugs: Yes (ref: No)**	1.15 (1.01-1.30)	0.0310	1.13 (1.00-1.28)	0.0490
**Gout medications: Yes (ref: No)**	NA (NA-NA)	NA		
**Antiepileptics: Yes (ref: No)**	0.8 (0.61-1.06)	0.1200		
**Antipsychotics: Yes (ref: No)**	0.88 (0.75-1.02)	0.0960	0.86 (0.74-1.00)	0.0470
**inhaled anti-obstructive drugs: Yes (ref: No)**	0.98 (0.85-1.14)	0.8200		
**inhaled steroids: Yes (ref: No)**	1.04 (0.78-1.40)	0.7700		
**other COPD drugs: Yes (ref: No)**	1.56 (1.08-2.26)	0.0180	1.46 (1.03-2.07)	0.0320
**Systemic Antihistamines: Yes (ref: No)**	0.87 (0.71-1.08)	0.2100		

Table S41: Results of the simple and multivariable Competing risk regression observing hospital discharge with competing event in-hospital mortality comparing propensity score matched COVID-19 and Influenza patients of age 41 to 64.

	Simple		Multivariable	
**Confounder**	**HR (CI)**	**p- value**	**HR (CI)**	**p- value**
**Group: Flu (ref: Covid)**	1.81 (1.71-1.93)	<0.001	1.84 (1.73-1.96)	<0.001
**Age Group: 56-60 (ref: 61-64)**	1.07 (1.00-1.15)	0.0490	1.09 (1.01-1.17)	0.0200
**Age Group: 51-55 (ref: 61-64)**	1.22 (1.13-1.31)	<0.001	1.23 (1.14-1.32)	<0.001
**Age Group: 46-50 (ref: 61-64)**	1.31 (1.2-1.42)	<0.001	1.31 (1.21-1.43)	<0.001
**Age Group: 41-55 (ref: 61-64)**	1.44 (1.31-1.6)	<0.001	1.47 (1.33-1.63)	<0.001
**Sex: W (ref: M)**	1.15 (1.10-1.21)	<0.001	1.15 (1.09-1.21)	<0.001
**Number Medication groups: 2-5 (ref: 0-1)**	0.83 (0.78-0.88)	<0.001	0.87 (0.81-0.94)	<0.001
**Number Medication groups: 6-10 (ref: 0-1)**	0.64 (0.59-0.69)	<0.001	0.79 (0.69-0.91)	<0.001
**Number Medication groups: >=11 (ref: 0-1)**	0.68 (0.57-0.81)	<0.001	0.96 (0.74-1.25)	0.7600
**Anticoagulants: Yes (ref: No)**	0.97 (0.90-1.05)	0.4900		
**Antibiotics, Antivirals, Antiprotozoals or Anthelmintics: Yes (ref: No)**	1.04 (0.98-1.10)	0.1700		
**Insulins and other Antidiabetics: Yes (ref: No)**	0.84 (0.77-0.90)	<0.001	0.8 (0.74-0.87)	<0.001
**"heart" drugs: Yes (ref: No)**	1.14 (1.01-1.29)	0.0410	1.11 (0.98-1.26)	0.0920
**Antihypertensives incl. Diuretics and Renin-angiotensin-aldosterone system inhibitors: Yes (ref: No)**	0.93 (0.87-0.99)	0.0180	0.93 (0.87-0.99)	0.0180
**Beta Blockers: Yes (ref: No)**	0.95 (0.88-1.03)	0.1900		
**Statins, Fibrates incl. Proprotein convertase subtilisin/kexin type 9 inhibitors and Inclisiran: Yes (ref: No)**	1.09 (1.02-1.17)	0.0094	1.12 (1.05-1.20)	0.0014
**Immunosuppressants and Immunomodulators: Yes (ref: No)**	0.96 (0.85-1.08)	0.4700		
**Systemic Steroids: Yes (ref: No)**	0.95 (0.88-1.02)	0.1800		
**Chemotherapy: Yes (ref: No)**	0.99 (0.81-1.21)	0.9000		
**Iron supplements, Erythropoietic stimulating agents, Vitamin B12, folic acid: Yes (ref: No)**	0.89 (0.79-1.00)	0.0490	0.94 (0.83-1.06)	0.3000
**Antacids incl. Antihistamines: Yes (ref: No)**	0.93 (0.87-1.00)	0.0600	0.94 (0.88-1.01)	0.1000
**Vitamin D and other Vitamin supplements: Yes (ref: No)**	0.85 (0.78-0.92)	<0.001	0.87 (0.80-0.94)	<0.001
**Caplacizumab: Yes (ref: No)**	NA (NA-NA)	NA		
**Systemic Hemostatics: Yes (ref: No)**	0.88 (0.3-2.57)	0.8100		
**Hereditary angioedema Therapeutics: Yes (ref: No)**	NA (NA-NA)	NA		
**Peripheral Vasodilators: Yes (ref: No)**	0.98 (0.73-1.32)	0.8900		
**Immunoglobulins: Yes (ref: No)**	4.32 (3.91-4.77)	<0.001	4.72 (4.20-5.31)	<0.001
**Interferons and CSF: Yes (ref: No)**	1.04 (0.89-1.21)	0.6400		
**NSAR and other anti-inflammatory drugs: Yes (ref: No)**	1.16 (1.10-1.23)	<0.001	1.14 (1.08-1.21)	<0.001
**Gout medications: Yes (ref: No)**	0.85 (0.72-1.02)	0.0820	0.84 (0.71-1.01)	0.0650
**Antiepileptics: Yes (ref: No)**	0.91 (0.83-0.99)	0.0340	0.93 (0.85-1.02)	0.1400
**Antipsychotics: Yes (ref: No)**	0.91 (0.85-0.96)	0.0017	0.91 (0.86-0.97)	0.0052
**inhaled anti-obstructive drugs: Yes (ref: No)**	0.93 (0.88-0.99)	0.0180	0.88 (0.83-0.94)	<0.001
**inhaled steroids: Yes (ref: No)**	1.18 (1.05-1.31)	0.0036	1.16 (1.04-1.30)	0.0083
**other COPD drugs: Yes (ref: No)**	1.08 (0.90-1.29)	0.4000		
**Systemic Antihistamines: Yes (ref: No)**	1.01 (0.90-1.13)	0.9200		

Table S42: Results of the simple and multivariable Competing risk regression observing hospital discharge with competing event in-hospital mortality comparing propensity score matched COVID-19 and Influenza patients of age 65 to 74.

	Simple		Multivariable	
**Confounder**	**HR (CI)**	**p- value**	**HR (CI)**	**p- value**
**Group: Flu (ref: Covid)**	2.34 (2.2-2.49)	<0.001	2.39 (2.24-2.54)	<0.001
**Age Group: 65-70 (ref: 71-74)**	1.14 (1.07-1.2)	<0.001	1.13 (1.07-1.19)	<0.001
**Sex: W (ref: M)**	1.19 (1.13-1.26)	<0.001	1.19 (1.13-1.26)	<0.001
**Number Medication groups: 2-5 (ref: 0-1)**	0.94 (0.87-1.01)	0.0890	0.95 (0.87-1.04)	0.2700
**Number Medication groups: 6-10 (ref: 0-1)**	0.72 (0.67-0.78)	<0.001	0.82 (0.72-0.94)	0.0032
**Number Medication groups: >=11 (ref: 0-1)**	0.64 (0.56-0.72)	<0.001	0.79 (0.65-0.97)	0.0270
**Anticoagulants: Yes (ref: No)**	0.93 (0.87-0.99)	0.0310	0.92 (0.86-0.99)	0.0230
**Antibiotics, Antivirals, Antiprotozoals or Anthelmintics: Yes (ref: No)**	1.04 (0.98-1.10)	0.1700		
**Insulins and other Antidiabetics: Yes (ref: No)**	0.92 (0.86-0.98)	0.0140	0.90 (0.84-0.97)	0.0036
**"heart" drugs: Yes (ref: No)**	0.98 (0.89-1.08)	0.6500		
**Antihypertensives incl. Diuretics and Renin-angiotensin-aldosterone system inhibitors: Yes (ref: No)**	1.04 (0.98-1.11)	0.2000		
**Beta Blockers: Yes (ref: No)**	0.95 (0.89-1.02)	0.1300		
**Statins, Fibrates incl. Proprotein convertase subtilisin/kexin type 9 inhibitors and Inclisiran: Yes (ref: No)**	1.12 (1.06-1.19)	<0.001	1.14 (1.07-1.21)	<0.001
**Immunosuppressants and Immunomodulators: Yes (ref: No)**	0.94 (0.81-1.08)	0.3800		
**Systemic Steroids: Yes (ref: No)**	1.05 (0.97-1.13)	0.2200		
**Chemotherapy: Yes (ref: No)**	0.95 (0.81-1.10)	0.4600		
**Iron supplements, Erythropoietic stimulating agents, Vitamin B12, folic acid: Yes (ref: No)**	0.84 (0.75-0.94)	0.0016	0.89 (0.80-0.99)	0.0370
**Antacids incl. Antihistamines: Yes (ref: No)**	0.89 (0.82-0.95)	0.0014	0.90 (0.83-0.97)	0.0039
**Vitamin D and other Vitamin supplements: Yes (ref: No)**	0.98 (0.91-1.06)	0.6500		
**Caplacizumab: Yes (ref: No)**	NA (NA-NA)	NA		
**Systemic Hemostatics: Yes (ref: No)**	1.29 (0.95-1.75)	0.1100		
**Hereditary angioedema Therapeutics: Yes (ref: No)**	NA (NA-NA)	NA		
**Peripheral Vasodilators: Yes (ref: No)**	1.18 (0.94-1.48)	0.1600		
**Immunoglobulins: Yes (ref: No)**	1.51 (0.93-2.42)	0.0920	1.42 (0.92-2.20)	0.1100
**Interferons and CSF: Yes (ref: No)**	0.90 (0.75-1.08)	0.2500		
**NSAR and other anti-inflammatory drugs: Yes (ref: No)**	1.21 (1.14-1.28)	<0.001	1.19 (1.11-1.26)	<0.001
**Gout medications: Yes (ref: No)**	0.97 (0.86-1.09)	0.6200		
**Antiepileptics: Yes (ref: No)**	0.82 (0.75-0.89)	<0.001	0.86 (0.79-0.93)	<0.001
**Antipsychotics: Yes (ref: No)**	0.86 (0.81-0.92)	<0.001	0.89 (0.84-0.95)	<0.001
**inhaled anti-obstructive drugs: Yes (ref: No)**	0.97 (0.91-1.04)	0.4100		
**inhaled steroids: Yes (ref: No)**	1.12 (0.99-1.26)	0.0750	1.05 (0.93-1.18)	0.4600
**other COPD drugs: Yes (ref: No)**	0.96 (0.80-1.17)	0.7200		
**Systemic Antihistamines: Yes (ref: No)**	1.03 (0.92-1.16)	0.5500		

Table S43: Results of the simple and multivariable Competing risk regression observing hospital discharge with competing event in-hospital mortality comparing propensity score matched COVID-19 and Influenza patients of age 75 and older.

	Simple		Multivariable	
**Confounder**	**HR (CI)**	**p- value**	**HR (CI)**	**p- value**
**Group: Flu (ref: Covid)**	2.52 (2.42-2.62)	<0.001	2.54 (2.44-2.64)	<0.001
**Age Group: 86-90 (ref: >= 91)**	1.25 (1.16-1.34)	<0.001	1.23 (1.15-1.32)	<0.001
**Age Group: 81-85 (ref: >= 91)**	1.50 (1.40-1.60)	<0.001	1.47 (1.37-1.57)	<0.001
**Age Group: 75-80 (ref: >= 91)**	1.75 (1.64-1.87)	<0.001	1.69 (1.58-1.80)	<0.001
**Sex: W (ref: M)**	1.16 (1.12-1.21)	<0.001	1.18 (1.13-1.22)	<0.001
**Number Medication groups: 2-5 (ref: 0-1)**	0.95 (0.89-1.00)	0.0660	0.95 (0.89-1.02)	0.1400
**Number Medication groups: 6-10 (ref: 0-1)**	0.84 (0.80-0.90)	<0.001	0.90 (0.82-0.99)	0.0320
**Number Medication groups: >=11 (ref: 0-1)**	0.70 (0.63-0.78)	<0.001	0.83 (0.72-0.97)	0.0200
**Anticoagulants: Yes (ref: No)**	0.99 (0.95-1.04)	0.7000		
**Antibiotics, Antivirals, Antiprotozoals or Anthelmintics: Yes (ref: No)**	0.99 (0.95-1.03)	0.7100		
**Insulins and other Antidiabetics: Yes (ref: No)**	0.96 (0.91-1.00)	0.0690	0.92 (0.88-0.97)	0.0012
**"heart" drugs: Yes (ref: No)**	0.97 (0.92-1.02)	0.1900		
**Antihypertensives incl. Diuretics and Renin-angiotensin-aldosterone system inhibitors: Yes (ref: No)**	1.06 (1.01-1.10)	0.0150	1.05 (1.01-1.10)	0.0270
**Beta Blockers: Yes (ref: No)**	0.94 (0.90-0.98)	0.0069	0.92 (0.88-0.96)	<0.001
**Statins, Fibrates incl. Proprotein convertase subtilisin/kexin type 9 inhibitors and Inclisiran: Yes (ref: No)**	1.15 (1.10-1.20)	<0.001	1.14 (1.09-1.19)	<0.001
**Immunosuppressants and Immunomodulators: Yes (ref: No)**	0.98 (0.85-1.13)	0.7500		
**Systemic Steroids: Yes (ref: No)**	0.99 (0.94-1.05)	0.7100		
**Chemotherapy: Yes (ref: No)**	1.01 (0.93-1.11)	0.7700		
**Iron supplements, Erythropoietic stimulating agents, Vitamin B12, folic acid: Yes (ref: No)**	0.85 (0.8-0.91)	<0.001	0.87 (0.81-0.92)	<0.001
**Antacids incl. Antihistamines: Yes (ref: No)**	0.98 (0.94-1.03)	0.4400		
**Vitamin D and other Vitamin supplements: Yes (ref: No)**	0.97 (0.93-1.02)	0.2600		
**Caplacizumab: Yes (ref: No)**	NA (NA-NA)	NA		
**Systemic Hemostatics: Yes (ref: No)**	0.47 (0.19-1.14)	0.0940	0.43 (0.18-1.05)	0.0640
**Hereditary angioedema Therapeutics: Yes (ref: No)**	NA (NA-NA)	NA		
**Peripheral Vasodilators: Yes (ref: No)**	1.13 (0.91-1.40)	0.2600		
**Immunoglobulins: Yes (ref: No)**	1.04 (0.39-2.80)	0.9400		
**Interferons and CSF: Yes (ref: No)**	0.92 (0.77-1.11)	0.3700		
**NSAR and other anti-inflammatory drugs: Yes (ref: No)**	1.10 (1.05-1.15)	<0.001	1.09 (1.04-1.14)	<0.001
**Gout medications: Yes (ref: No)**	0.91 (0.85-0.99)	0.0190	0.90 (0.84-0.97)	0.0078
**Antiepileptics: Yes (ref: No)**	0.95 (0.9-1.00)	0.0440	0.95 (0.9-1.00)	0.0700
**Antipsychotics: Yes (ref: No)**	0.90 (0.87-0.94)	<0.001	0.91 (0.87-0.94)	<0.001
**inhaled anti-obstructive drugs: Yes (ref: No)**	0.95 (0.90-1.00)	0.0350	0.94 (0.89-0.98)	0.0086
**inhaled steroids: Yes (ref: No)**	1.03 (0.92-1.14)	0.6500		
**other COPD drugs: Yes (ref: No)**	0.85 (0.68-1.06)	0.1600		
**Systemic Antihistamines: Yes (ref: No)**	0.95 (0.88-1.03)	0.1800		

Figure S6: Summary of Hazard ratios and 95%-confidence intervals for group, age, sex and medication group-effects comparing COVID-19 and Influenza patients for the outcome hospital discharge in the age groups. Group: A hazard ratio larger than one is indicating a higher risk of the event in the Influenza group. Age: A hazard ratio larger than one is indicating a higher risk in younger age groups. Sex: A hazard ratio larger than one is indicating a higher risk in female patients. Medication Groups: a hazard ratio larger than one is indicating a higher risk in patients with a larger number of prescribed medication groups. Significant comparisons (with $p<0.05$) are marked with a blue star.

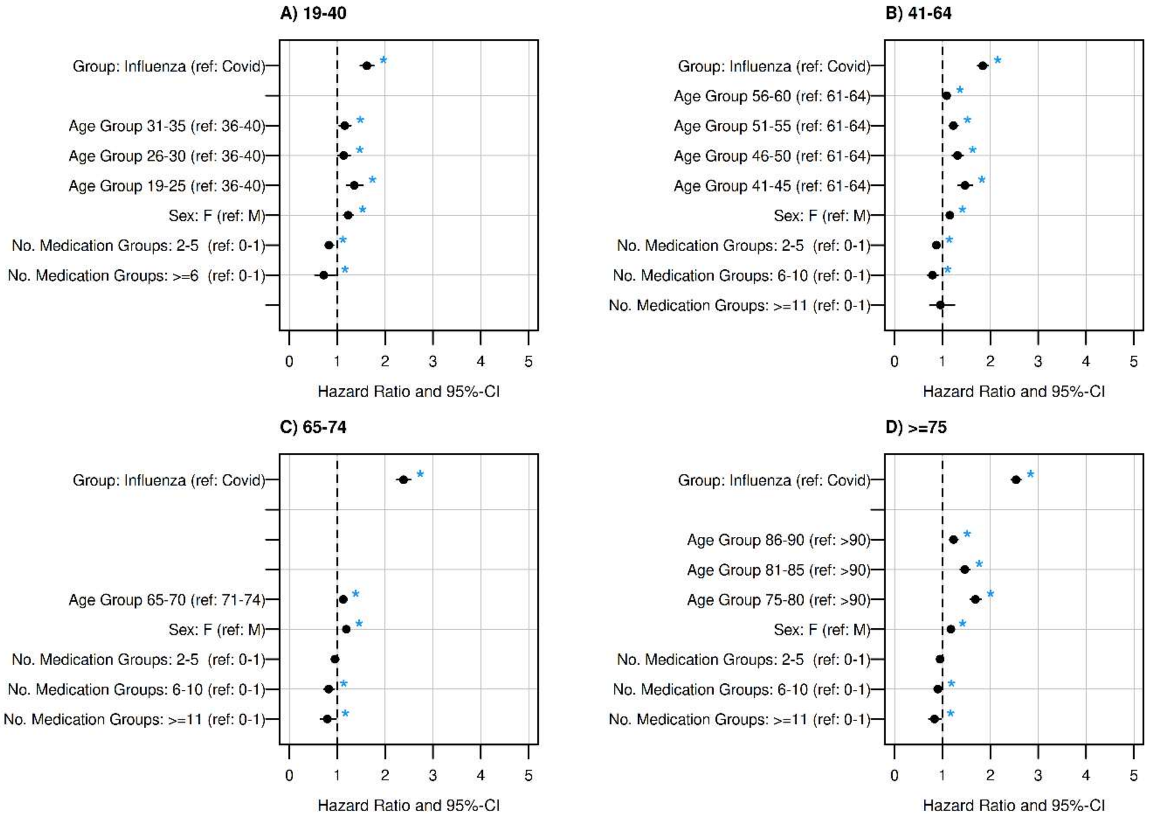

Figure S7: Kaplan-Meier curves for all-cause mortality (first column), in-hospital mortality (second column) and all-cause death conditional on hospital-survival (third column) separately for age groups (different rows) as well as for PSM-matched COVID-19 (red) and Influenza (blue) cohorts.

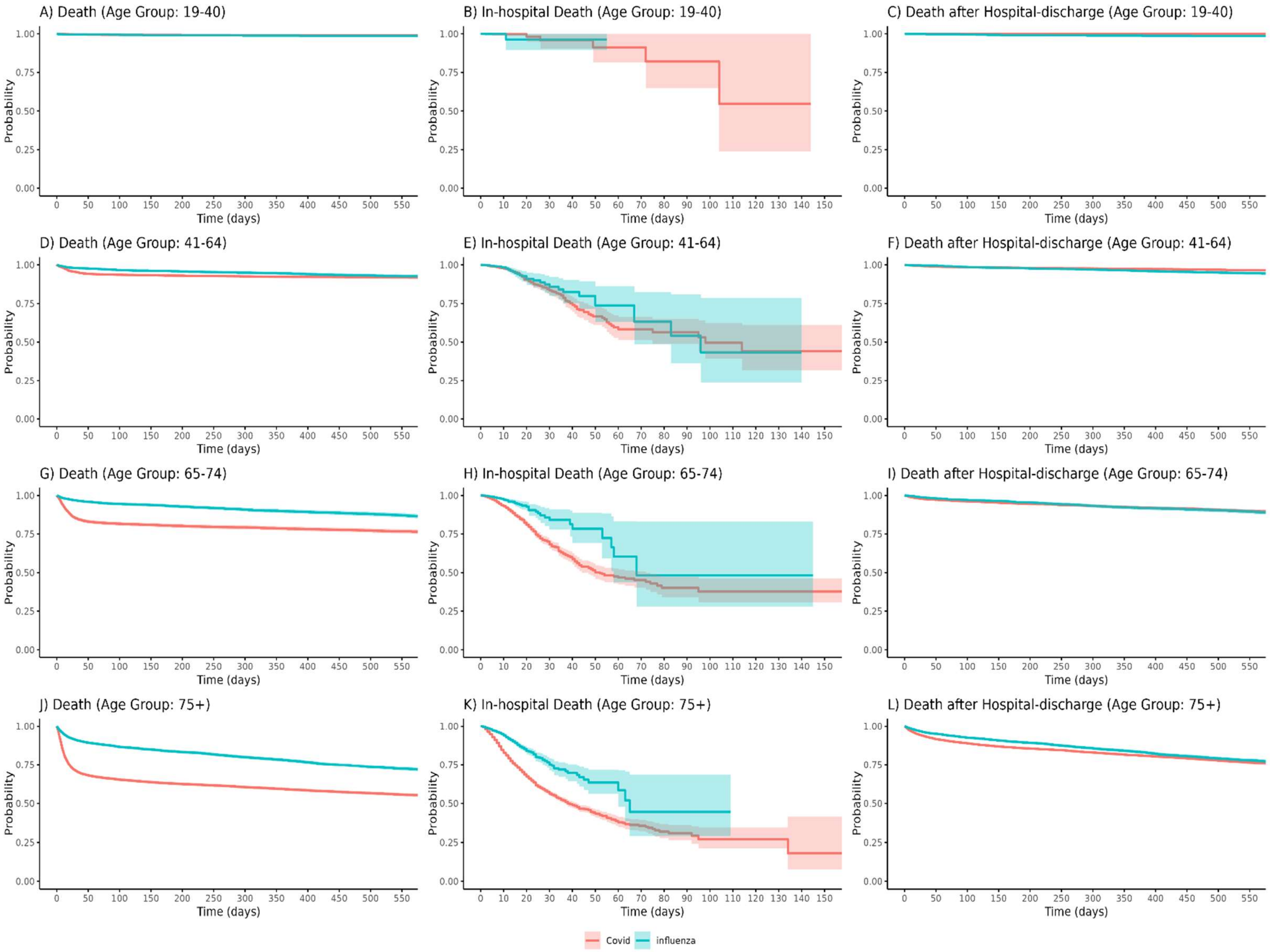

Figure S8: Kaplan-Meier curves for all cause death for the number of prescribed medication groups for COVID-19 (first column) and Influenza patients (second column) separately for age groups (different rows). Note that for the age group 19-40, the groups 6-10 and 11+ were summarized in one group due to the low number of patients with 11+

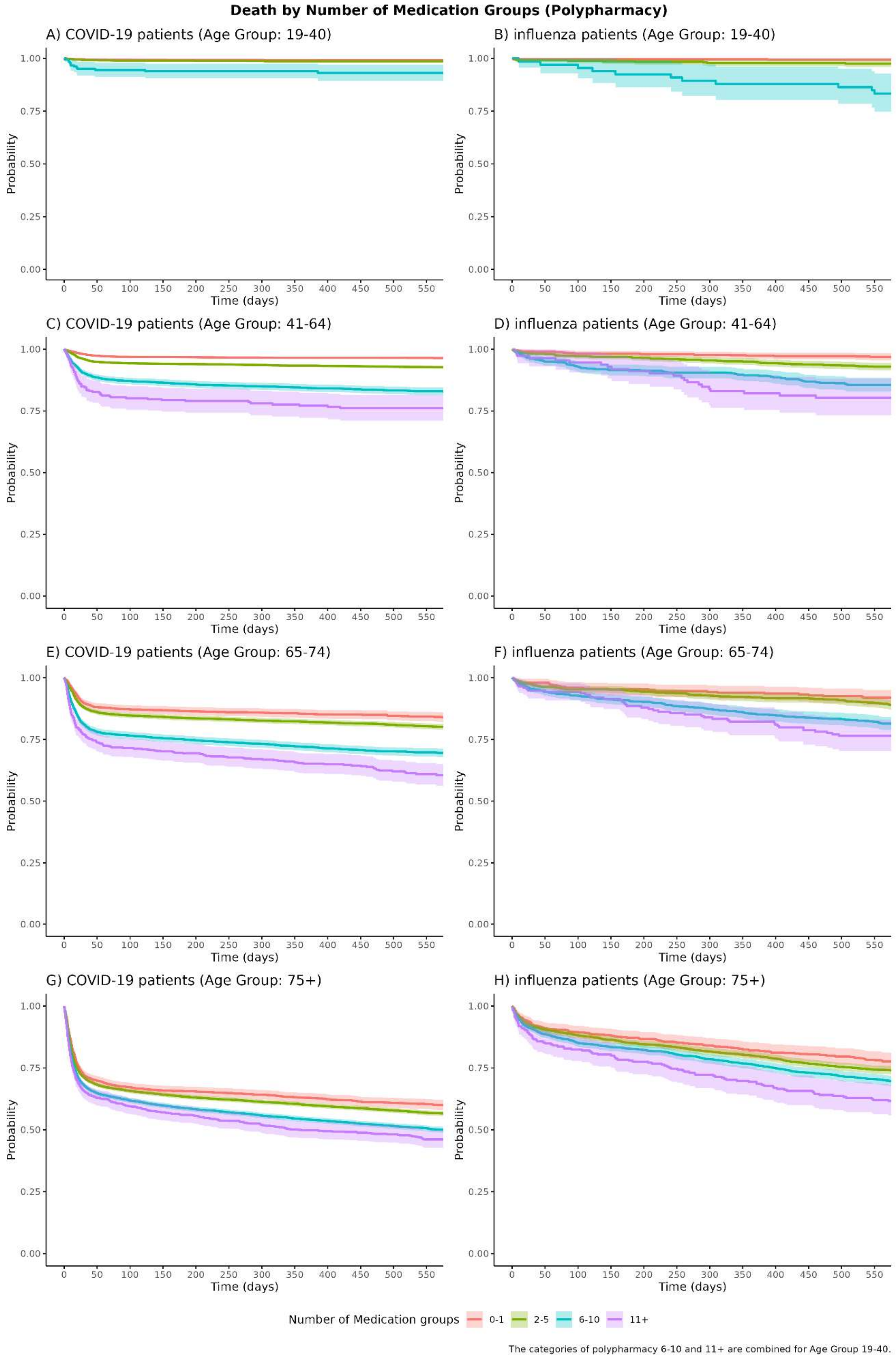

Figure S9: Percentage in-hospital deaths separately for age groups and years of hospital admission of COVID-19 (A) and Influenza (B) hospitalized patients. The number of hospital admissions is given in red and the absolute number of in-hospital deaths in black above the bars.

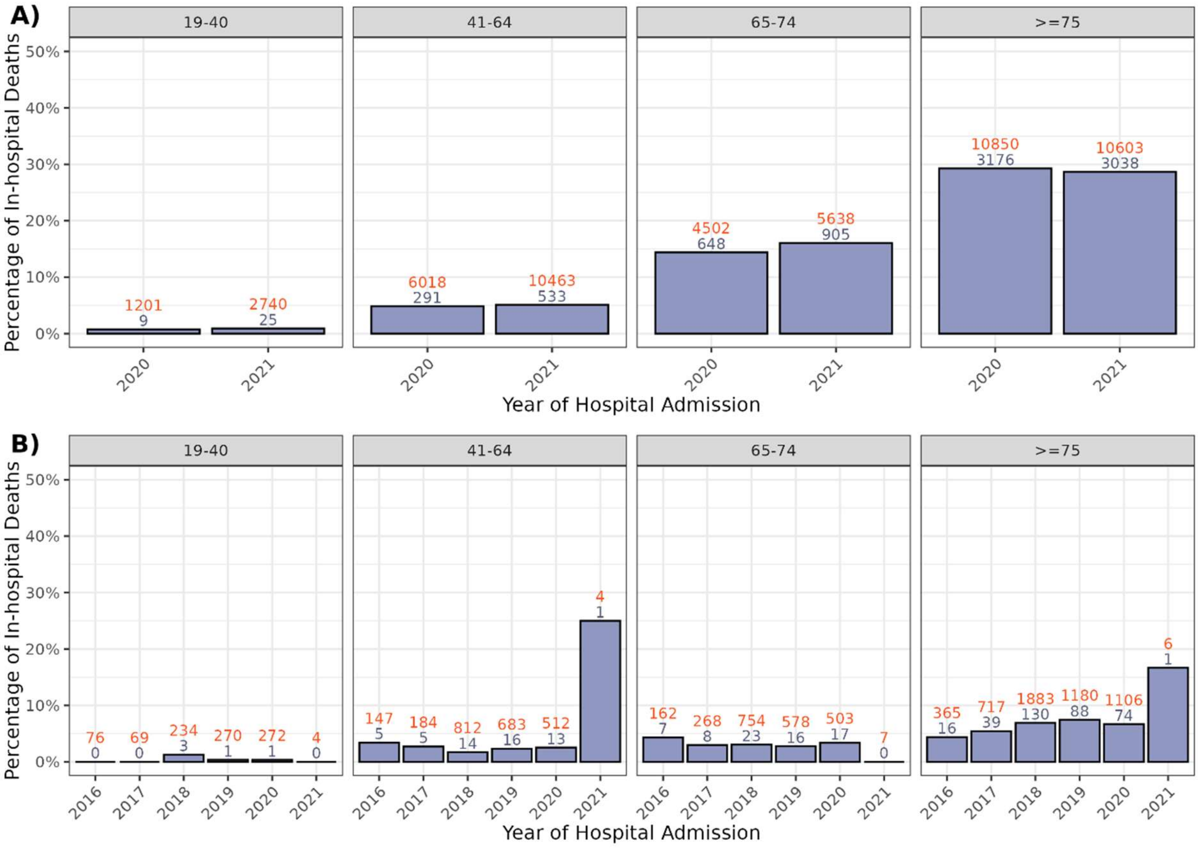

Figure S10: Cumulative incidence curves for hospital discharge (first column) and readmission (second column) separately for age groups (different rows) as well as for PSM-matched COVID-19 (red) and Influenza (blue) cohorts.

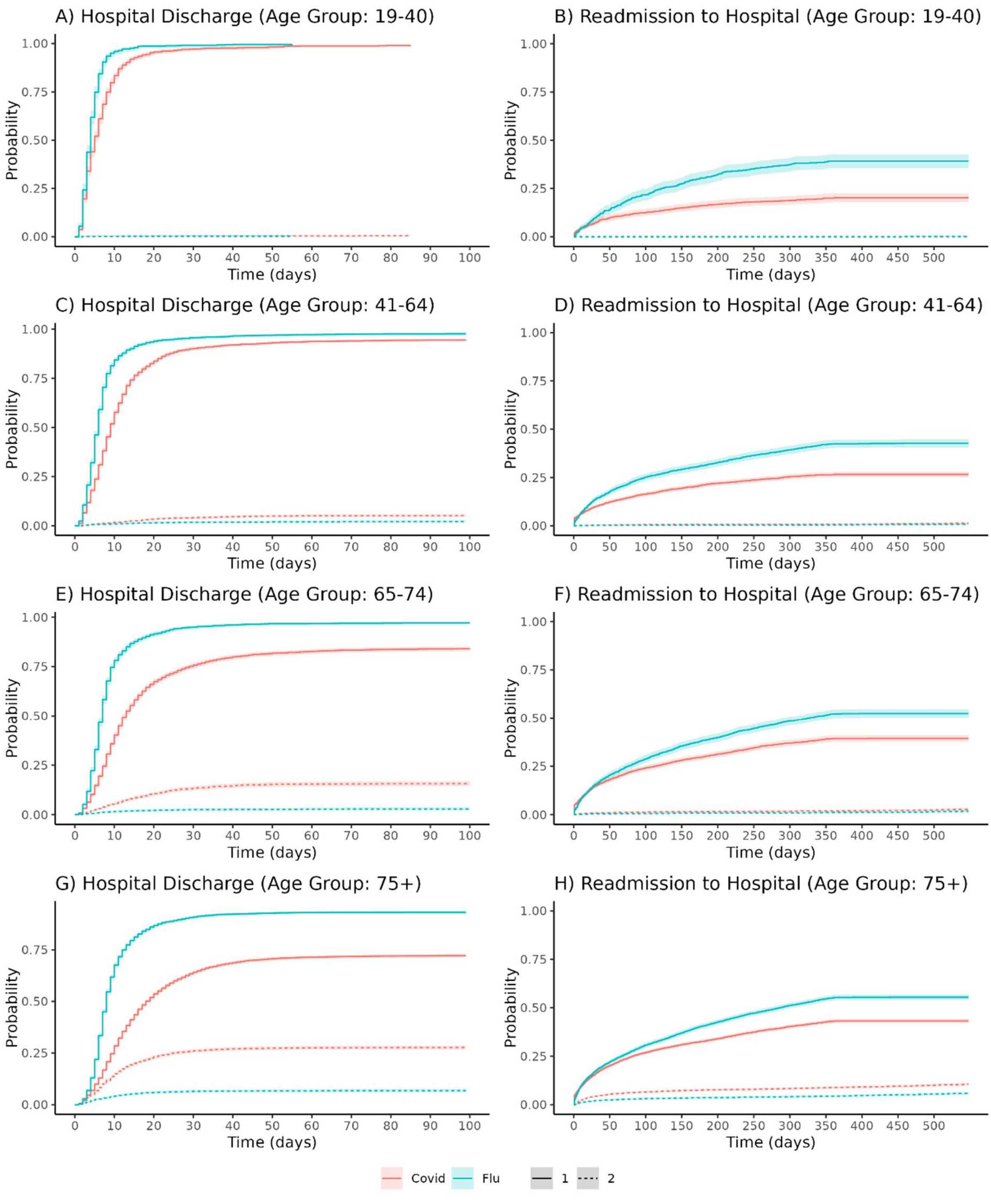

Supplement: Supplementary file 1 [file Data_Sheet_1.pdf]
